# Impact of environmental variables on Dubas bug infestation rate: A case study from the Sultanate of Oman

**DOI:** 10.1371/journal.pone.0178109

**Published:** 2017-05-30

**Authors:** Khalifa M. Al-Kindi, Paul Kwan, Nigel Andrew, Mitchell Welch

**Affiliations:** 1 School of Science and Technology, University of New England, Armidale, New South Wales, Australia; 2 Centre for Behavioural and Physiological Ecology, School of Environmental and Rural Sciences, University of New England, Armidale, New South Wales, Australia; Universidade Federal de Vicosa, BRAZIL

## Abstract

Date palm cultivation is economically important in the Sultanate of Oman, with significant financial investment coming from both the government and from private individuals. However, a global infestation of Dubas bug (*Ommatissus lybicus Bergevin*) has impacted the Middle East region, and infestations of date palms have been widespread. In this study, spatial analysis and geostatistical techniques were used to model the spatial distribution of Dubas bug infestations to (a) identify correlations between Dubas bug densities and different environmental variables, and (b) predict the locations of future Dubas bug infestations in Oman. Firstly, we considered individual environmental variables and their correlations with infestation locations. Then, we applied more complex predictive models and regression analysis techniques to investigate the combinations of environmental factors most conducive to the survival and spread of the Dubas bug. Environmental variables including elevation, geology, and distance to drainage pathways were found to significantly affect Dubas bug infestations. In contrast, aspect and hillshade did not significantly impact on Dubas bug infestations. Understanding their distribution and therefore applying targeted controls on their spread is important for effective mapping, control and management (e.g., resource allocation) of Dubas bug infestations.

## 1. Introduction

Date palm is one of oldest crop fruits in the world and has played a particularly important role in the economic yield of most Arab countries, which are arid and semi-arid [1], including the Sultanate of Oman (hereafter referred to as Oman) [2]. Palm fruits have a high nutritional value and many people in the Middle East depend on them for their livelihood [3].

Oman has a comparatively long growing season that normally extends from early May to November. However, date fruits production in Oman is being threatened by a variety of pests that can cause major damages including plant death, reduced yield, and falling production [4,5]. The Dubas bug (*Ommatissus lybicus* Bergevin, Homoptera: Tropiduchidae) represents one of the major pests responsible for the decline of date fruits production in the Middle East and North Africa [6–11]. Blumberg [7] identified the Tigris-Euphrates River Valley as the primary origin of the Dubas bug, which subsequently spread to other regions.

The Dubas bug (DB) is a sap feeding insect; both adults and nymphs suck the sap from date palms, thereby causing chlorosis [12–17]. There is also an indirect effect whereby honeydew secretions produced by the DB can promote the growth of black sooty mould on the foliage and consequently a reduction in the photosynthetic rates of date palms [7,18,19]. Prolonged high infestation level will result in the flagging and destruction of palm plantations [6,20–23].

Nymphs pass through five growth instars [13,19], with adult female DB reaching 5–6 mm and the males 3–3.5 mm in length [14,24]. Their colour is yellowish-green while the main distinguishing feature between males and females is the presence of spots on females; males have a more tapered abdomen and larger wings relative to the abdomen.

Two populations of DB are generated each year. The summer generation of nymphs hatch in mid-late April. After two months, they mature and lay eggs for the second generation, which lay dormant for approximately three months before hatching in late September. Blumberg [7] showed that eggs can hatch within 18–21 days in the summer, but can take up to 170 days in winter. Each female can produce more than 120 eggs, which are laid by insertion into holes in the tissue of date palm fronds at the end of each season. The eggs of DB are sensitive to temperature. The developmental time of a Dubas bug’s eggs has been studied at three different temperatures, 17.6, 27.5 and 32.4°C in Oman [25]. The results showed that a temperature of 27.5°C appeared to be the optimal temperature for the biological activities of this [25]. At 35°C, the biological processes of the pest are disrupted, thus leading to high mortality, particularly in females [6,26].

In Oman, vector control activities aim to exterminate or reduce DBs infestations have concentrated on the use of insecticides, including both ground and aerial sprays. However, use of the most effective pesticide is restricted owing to its side effects (e.g., irritation) [5]. In Israel, systemic carbamates (e.g., aldicarb and butocarboxim) have been used successful, while in Iraq dichlorvos (DDVP) injected directly into infected palms has been successful in suppressing the pest population [7]. However, these methods are expensive and can have negative environmental impacts on both non-target species, particularly the natural enemies of the DB (e.g., *Aprostocetus sp*. (Hymenoptera: Eulophidae), *Oligosita sp*. (Hymenoptera: Trichogammidae, and *Runcinia sp*. (Aranae: Thomsidae)), and on human health [27]. Research has shown that some pesticide residues can persist on the date fruits for up to sixty days after application [28–30]. Furthermore, chemical control measures have met with limited success in Oman, while Dubas bug continues to pose a major challenge to the agricultural industry.

To gain a thorough understanding of the life cycle and distribution of this pest, new research into the biology and ecology of the species is needed. To date, no published studies have focused on the spatial distribution of DB infestations, or the environmental variables associated with their distribution at the stress level. Furthermore, no previous studies have developed risk maps of DB distribution on a large scale, or have systematically analysed patterns of infestations at either a large-scale or orchard level. Understanding the distribution and affinity of DB to different environmental variables will play a key role in the mapping; control and management of DB infestations, including resource allocation (e.g., spray teams and field personnel).

The main objective of this study is to investigate the environmental variables impacting DB infestations in northern Oman. We considered the contributions of elevation, slope, aspect, soil type, water type, geology, hillshade, distance to the sea, and distance to drainage pathways in our study. Firstly, we investigate the correlations of infestation with each single variable in order to develop a correlation model. Next, based on this correlation model, we construct a more complex predictive model for assessing the relative impacts of the candidate environmental variables. The results of this study show that the presence and spread of DB are most conducive to a combination of variables rather than individual ones.

## 2. Materials and methods

### 2.1. Study area

**Fig 1:**
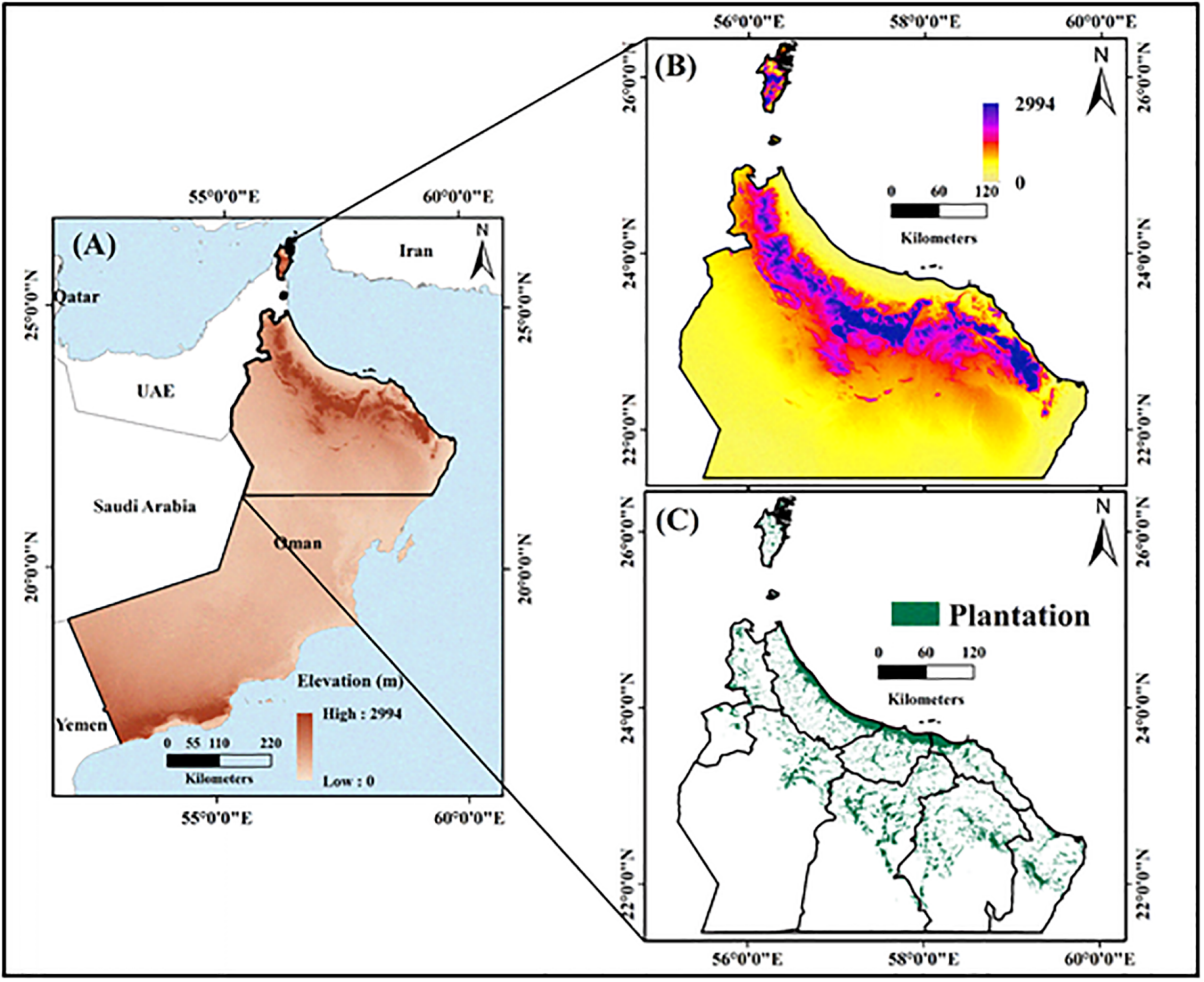
Maps of the study area, including: (A) topography and location of Oman, with the study area outlined by the black rectangle; (B) elevation change within the study area; and (C) distribution of date palm plantations in the study area (Esri ArcGIS 10.3).

The Sultanate of Oman, which covers an area of 309,500 km^2^, extends from 16°40’N to 26°20’N, and 51°50’E to 59°40’E. It occupies the south-eastern corner of the Arabian Peninsula (Fig 1). It has 3,165 km of coastline, extending from the Strait of Hormuz in the north to the border with the Republic of Yemen in the South. The coastline faces onto three different water bodies, namely the Arabian Sea, the Persian Gulf (also known as *Arabian Gulf*), and the Gulf of Oman [31].

To the west, Oman is bordered by the United Arab Emirates and the Kingdom of Saudi Arabia. Mountainous areas account for 15% of the land area, while desert plains and sandy areas cover 74%, agro-biodiversity areas cover 8%, and the coastal zone covers 3%, respectively. The location of Oman provides favourable conditions for agriculture, with land under agricultural use accounting for 8% of the territory and the economic output accounting for 14.6% of the GDP in 2008. According to the 2004–2005 soil survey conducted by the Ministry of Food and Agricultural (MFA), 22,230 km^2^ (equivalent 2.223 million ha) is optimal for agricultural activity, which represents ~7.5% of the country’s land area. Approximately 728.2 km^2^ (~72,820 ha) of the country is irrigated using the falaj irrigation system, where local springs or wadis (streams) underflow areas are cultivated with palm trees, banana, limes, alfalfa, and vegetables [32].

Oman has an arid climate, receiving less than 100 mm of rainfall per year; however, the mountainous parts of the country receive higher precipitation levels. As the dependent variable, DB infestations occur where palm trees are concentrated; therefore, in this study we focused on northern Oman (26°50N to 22°26N, and 55°50’E to 59°50E) which experiences high infestations.

### 2.2 Data collection

We used historical data from the Ministry of Agriculture and Fisheries (MFA) to identify regions or areas that have been infested by the DBs. In total, 840 sites were identified, and for each we used the MFA’s resource to collect data on their geographical locations (see Appendix A, S1 File), time of the survey, infestation levels, and the trapping procedure. The original data were not in a digital format and more than 400 hours were spent cleaning as well as modifying the data to create a geo-database that would be suitable for GIS analysis. Additional data (e.g., DBs population data) were collected from selected sampling points/stations, which were chosen based on distance to the sea and to streams, elevation, slope, hillshade, water type, soil type, and geology.

Insect trapping was performed at 80 sites (i.e., 10% of the total sites considered) in mid-March 2015 in order to collect both adult and nymph DB from the summer generation. Trapping was necessary because population dynamics played an important role and an absolute population estimate was needed. We used emergence traps (sticky yellow traps of 0.2 x 0.3 m in size) placed on palm fronds in maize fields. At each site, four date palms were selected and two traps apiece were attached to 6 fronds per palm. Traps were distributed at different levels in the crop row in order to capture both adult bugs and nymphs as they emerged from the fronds. These emergence traps were used to monitor the DB population throughout the growing season (spring-fall). Based on data from 2–3 sampling sites in each district (wilayat), we classified the DB infestations as zero, low, medium, or high.

A 30-m resolution Digital Elevation Model (DEM) was provided by the National Survey Authority, MOD. Soil and water data were provided by the Ministry of Regional Municipalities, Environment and Water Resources (MORMEWR). All data and GIS layers collected from the Sultanate of Omani Ministries and Departments were projected to WGS 1984 UTM zone 40.

### 2.3 Spatial analysis

#### 2.3.1 Interpolation

We used the ‘interpolate to raster tool’ in ArcGIS software package (version 10.3, ESRI, Redlands, CA) to combine the MFA data with our sampling data to create species population maps. This process was used to estimate DB abundance from geo-referenced infestation locations [33]. We used the Inverse Distance Weighted (IDW) interpolation method to create the abundance or surface maps. The IDW method imposes the condition that estimated values of a point are influenced more by nearby points than they are by points by at a greater distance [34]. Most importantly, all predicted values are within the range of maximum and minimum values of the known points. The general equation for the IDW method is expressed as:

$$Z_{0}=\frac{\sum_{i=1}^{s}z_{i}\frac{1}{d_{i}^{k}}}{\sum_{i=1}^{s}\frac{1}{d_{i}^{k}}}$$ (1)

where Z_0_ is the estimated value at point 0, *z*_*i*_ is the *z* value at known point *i*, *d*_*i*_ is the distance between point 0 and point *i*, *s* is the number of known points used in the estimation, and *k* is the specified point. The IDW technique is particularly appropriate in the case of irregularly distributed DB infestations because it can use an interpolated surface occurring only at the data points. Furthermore, IDW does not include outlier data values (e.g., negative numbers and exceptional values) that do not match the observed data values at each location.

#### 2.3.2 Spatial and surface analyses

A clip function was used to extract the study variables for each region of the study area, including water type, soil type, palm date plantations, and the 30-m resolution (DEM). The DEM was used to analyse elevation, slope, aspect, and hillshade, which represent the basic elements used when analysing and visualising ecological problems (e.g., forest and wildlife habitat suitability site analyses). Euclidean spatial analysis was used to calculate and create a Euclidean raster for each site (e.g., the distance to the sea and to streams). Vector datasets (e.g., soil type, water type, geology) were converted to raster datasets; thus allowing cell values to be extracted for locations specified in point features classes (e.g., DB sampling sites).

### 2.4 Statistical analysis

#### 2.4.1 Nearest neighbour statistics

We used nearest neighbour statistical (NNS) analysis to detect if patterns of DBs absence or where their density were random, regular, or clustered. This method measures the spacing by finding the distance between each point feature and its nearest neighbour [35]. NNS compares the mean spacing with the expected mean spacing calculated assuming a random distribution of point features. This spatial statistic can be computed by:

$$r=\frac{\sum_{i=1}^{n}\delta_{i}}{n}$$ (2)

The average spacing *r* is computed by summing the nearest neighbour distance and divided the total by the number of points *n*. If *r* is less than 1, the points pattern is more clustered than random and greater than 1 if the points pattern is more dispersed than random.

#### 2.4.2 Spatial autocorrelation

We used the Moran’s *I* autocorrelation method to measure the relationships between DB infestations and the environmental variables. Spatial autocorrelation considers the degree to which variables on the Earth’s surface are both spatially and numerically similar to other variables located nearby [36,37]. Moran’s *I* method helped us to determine if values and their associated features were clustered, randomly distributed, or dispersed. This method measures correlation in terms of proximity between adjacent features of the same phenomenon and is commonly used to detect the spatial order feature of points. Moran’s *I* can be expressed as:

$$I=\frac{\sum_{i=1}^{n}\sum_{i=1}^{m}w_{ij}(x_{i}-x^{-})(x_{j}-x^{-})}{s^{2}\sum_{i=1}^{n}\sum_{i=1}^{m}w_{ij}}$$ (3)

where *x*_*i*_ is the value at point *i*, *x* is the value at neighbouring point *j*, *w*_*ij*_ is a coefficient, *n* is the number of points, and *s*^*2*^ is the variance of x values. The values of the Moran’s *I* are anchored at the expected value *E(I)* for a random pattern, with *E(I)* methods 0 when the number of points *n* is large. Moran’s *I* is close to *E(I)* if the pattern is random, but is greater than *E(I)* if adjacent points tend to have similar values. The *z*-score associated with Moran’s *I* shows the likelihood that a point pattern could be the result of random chance. Moran’s *I* can also be applied to polygons and remains the same for computing the index value, but the coefficient is based on the relationships between polygons. Recent developments in spatial statistics have included the Local Indicator of Spatial Association (LISA), which is a local version of Moran’s *I* [38]. For each feature (point or polygon), LISA calculates for each feature an index value and a *z*-score. A high negative *z*-score suggests that the feature is adjacent to features of similar values.

#### 2.4.3 G-statistic for measuring clustering

We used the G-statistic to separate clusters of high and low values for different environmental features. The G-statistic method is based on a stated distance *d*. Weight can be based on some value of *G(d)*, which is used to evaluate statistical significance. This approach is similar to Moran’s *I* and a local version of the G-statistic is also available. The local G-statistic, denoted by *G*_i_*(d), is often described as a tool for “hotspot” analysis [39]. Moreover, a cluster of high positive *z*-scores suggests the presence of a cluster of hotspot values. In contrast, a cluster of low positive *z*-scores suggests the presence of a cluster of cold spot values. G-statistic based on a specified distance *d* is defined as:

$$G(d)=\frac{\sum \sum w_{ij}(d)x_{i}x_{j}}{\sum \sum x_{i}xj},i\neq j$$ (4)

where *x*_*i*_ is the value at location *i*, *x*_*j*_ is the value at location *j*, plus if *j* is within distance *d* of *i* and *w*_*ij*_ *(d)* is the spatial weight based on some weighted distance between 1 and 0. The expected value of *E(G)* is:

$$E(G)=\frac{\sum \sum w_{ij}(d)}{n(n-1)}$$ (5)

where *E(G(d))* is typically a very small value when *n* is huge. A high *G*(*d*) value proposes a clustering of high values, and a low *G*(*d*) value proposes a clustering of low values.

We applied the Local Moran’s *I* statistic to detect the accurate locations of ‘hotspot’ clusters of DB infestations. We used a surface analysis function to create a continuous map from the z-scores to present a generalised view of hotspots and cold spots. This approach allowed us to identify features with high values that might represent a statistically significant hotspot.

To be a statistically significant hotspot, a feature should have a high value and be enclosed or surrounded by other features with similarly high values [6]. We used the output of hotspot values as the input for incremental spatial autocorrelation analysis in order to measure a series of distances and to create a line chart of those distances and their corresponding *z*-scores. This method provides a good conceptualization of the relationships between hotspot infestation and distance (i.e. distance to sea, streams, and water type) [40–42]. Since *z*-scores mirror the intensity of spatial clustering, statistically significant peak *z*-scores indicate distances where spatial processes that promote clustering are most pronounced. These peak distances are often appropriate values for use in tools with a distance band.

#### 2.4.4 Exploratory regression

We used exploratory regression tools to model relationships by using all possible combinations for a given list of candidate explanatory variables to select the appropriate dependent (DB infestation) and independent variables (elevation, slope, aspect, hillshade, soil type, geological features, water type, distance to the sea, and distance to streams). We evaluated all possible combinations of variables in order to construct the most robust model for solving DB problems and to answer questions on DB infestations. The exploratory regression (ER) can be calculated by the following equation:

$$\gamma =\beta_{0}+\beta_{1}\chi_{1}+\beta_{2}\chi_{2}+\beta_{3}\chi_{3}+\ldots .+\beta_{n}\chi_{n}+\varepsilon$$ (6)

where *γ* is the independent variable, *β* are coefficient values, χ are explanatory variables, and ε are residuals. Geographically weighted regression (GWR) was used to produce maps of the coefficients, *r*^*2*^ values, standard residuals, and predictions [43], detailing a good sense of the relationships between DB infestations (dependent) and environmental variables (independent) across the study area.

## 3. Results and discussion

### 3.1. Spatial and surface analysis

**Fig 2:**
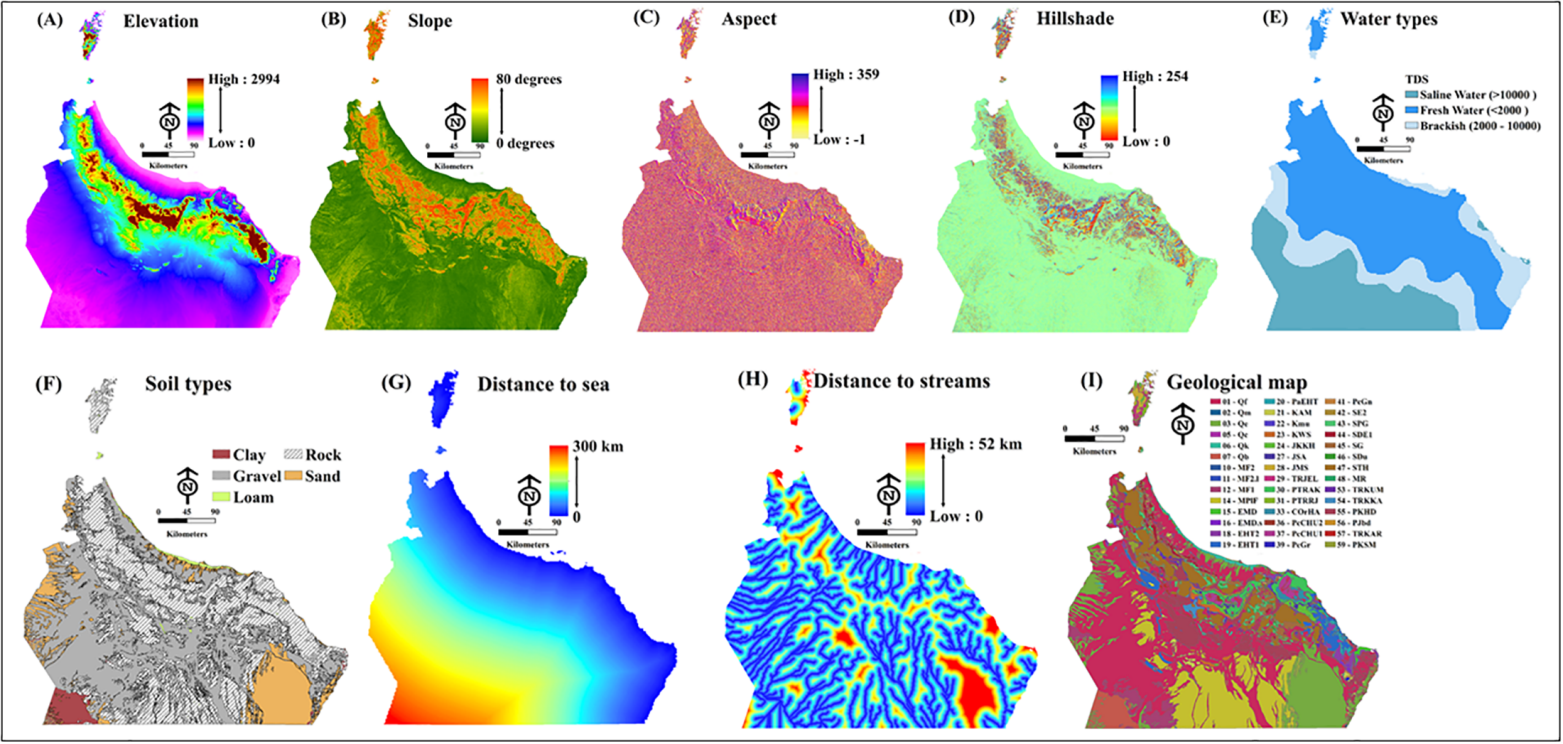
Surface analysis maps of the study area for: (A) elevation, (B) slope, (C) aspect, (D), hillshade, (E) water type, (F) soil type, (G) Euclidean distance to the sea, (H) Euclidean distance to streams, and (I) geology (Esri GISTM 10.3).

The elevation raster map produced in this study contains five elevation classes of equal interval (Fig 2a), which indicate the percentage occupation of the landscape:-112-234 m (40%), 235–484 m (25%), 485–838 m (15%), 839–1408 m (12%), and 1409–2994 m (8%).

The slope raster map contains five equally spaced slope classes between 80° (the steepest slope class) and 0° (Fig 2b). The aspect raster map contains ten aspect classes of equal interval, ranging from 359°N for the highest aspect to -1° for flat zones, or zones with no data (Fig 2c). The water vector map contains three categories of water class, classified based on total dissolved solid (TDS) content (Fig 2e): brackish water (2000–10,000 TDS), fresh water (< 2000 TDS), and saline water (>1000 TDS). The soil vector map contains five soil type categories: clay, gravel, loam, rock, and sandy soils (Fig 2f).

The map of Euclidean raster distance to the sea contains six distance classes, where 0 km is closest to the sea distance, and 300 km is furthest from the sea (Fig 2g). The map of Euclidean raster distance to streams contains six distance classes, where 0 km is closest to a stream and 30 km is furthest from a stream buffer line (Fig 2h). The geological vector map contains 59 geological classes (Fig 2i).

### 3.2 Spatial statistics

#### 3.2.1 Nearest neighbour statistics

The results of NNS analysis, in which the nearest neighbour ratio was 0.328347, showed that the expected mean distance or spacing of DB infestations distribution was greater than the observed mean, and that the difference was less than zero (i.e., a negative number; Table 1). These results indicate clustered distribution of DBs infestations.

**Table 1:** Mean nearest neighbour distribution of Dubas bug infestations.

Observed mean distance	1791.949 meter
Expected mean distance	5457.4916 meter
*p*-value	< 0.01
*z*-score	- 40

#### 3.2.2 Autocorrelation analysis

**Fig 3:**
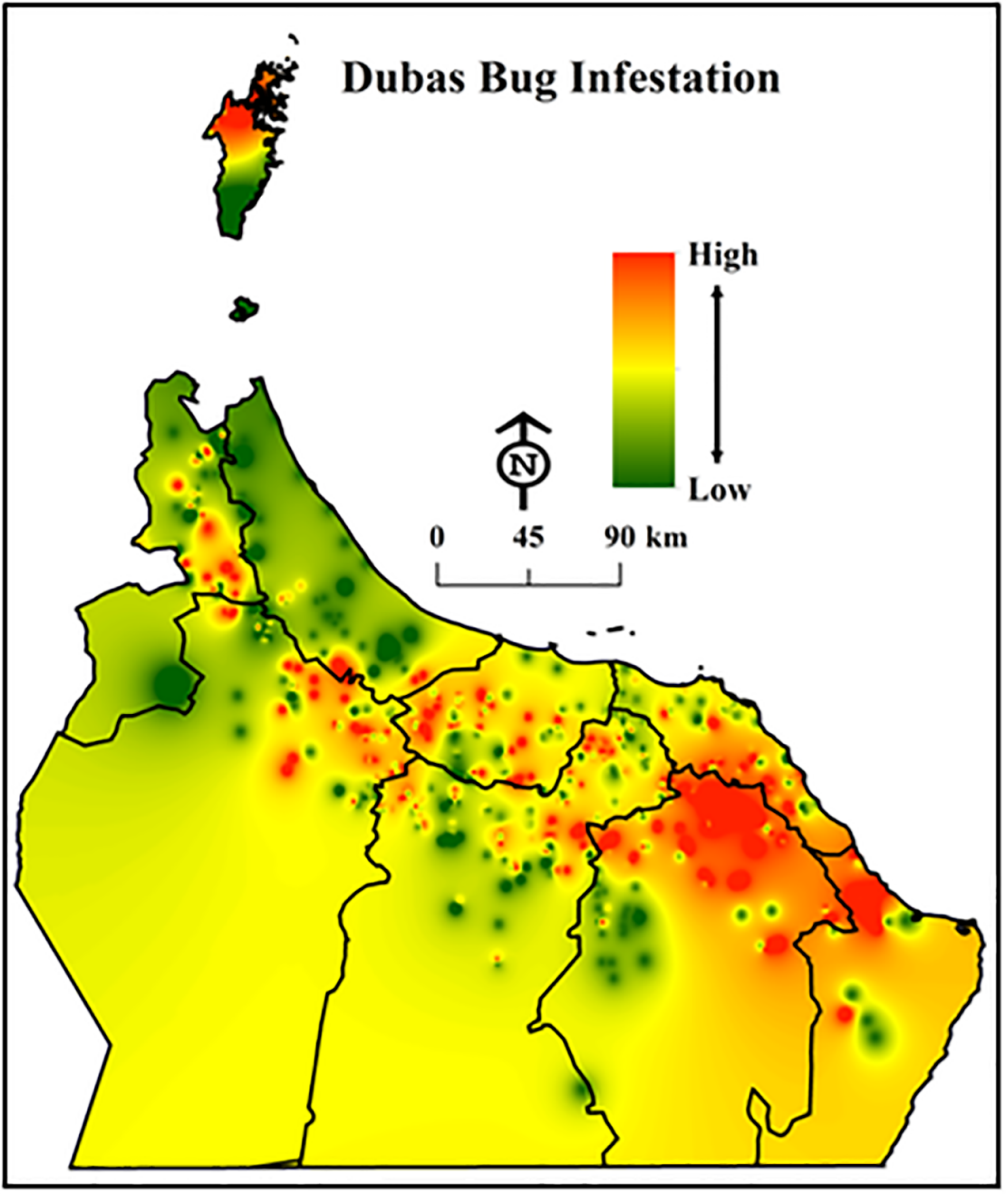
Raster map layer illustrating interpolated values of Dubas bug infestations in northern Oman (Esri ArcGIS 10.3).

We used the interpolated IDW raster map layers to conduct autocorrelation analysis in order to determine cell values, cell statistics, and types of spatial distribution exhibited by the DBs (Fig 3).

Autocorrelation found between DB infestations and some environmental features (i.e., water type, elevation, slope, soil type, geology, distance to the sea and distance to stream) were observed (Table 2). For example, *p*-values (probability) for elevation and geology were (*p* < 0.01), while the *z*-scores (standard deviation) were < 2.58, indicating a confidence level of more than 99%. In contrast, there was a weaker relationship between DB infestations and other variables (e.g., aspect and hillshade). However, autocorrelation cannot identify environmental variables with high or low cluster values, and for this a G-statistic was used.

**Table 2:** Moran’s *I* correlation coefficients between Dubas bugs and environmental features.

Variables	Moran’s *I*	Variance	*p*-value	*z*-score	Sign
TDS (water)	0.1559	0.00007	0.03*	61.061	Cluster
Elevation	0.2946	0.00007	0.01*	114.220	Cluster
Slope	0.0076	0.00007	0.07*	3.388	Cluster
Aspect	- 00003	0.00007	0.79	0.266	Random
Soil Types	0.0342	0.00007	0.01*	13.657	Cluster
Geology	0.0370	0.00007	0.01*	14.740	Cluster
Distance to Sea	0.4241	0.00007	0.01*	164.519	Cluster
Distance to stream	0.1438	0.00007	0.01*	56.552	Cluster
Hillshade	- 0.0103	0.00007	0.33	- 0.972	Random

* An asterisk next to a number indicates a statistically significant p-value (p < 0.01)

#### 3.2.3 G-statistic for measuring clustering

**Fig 4:**
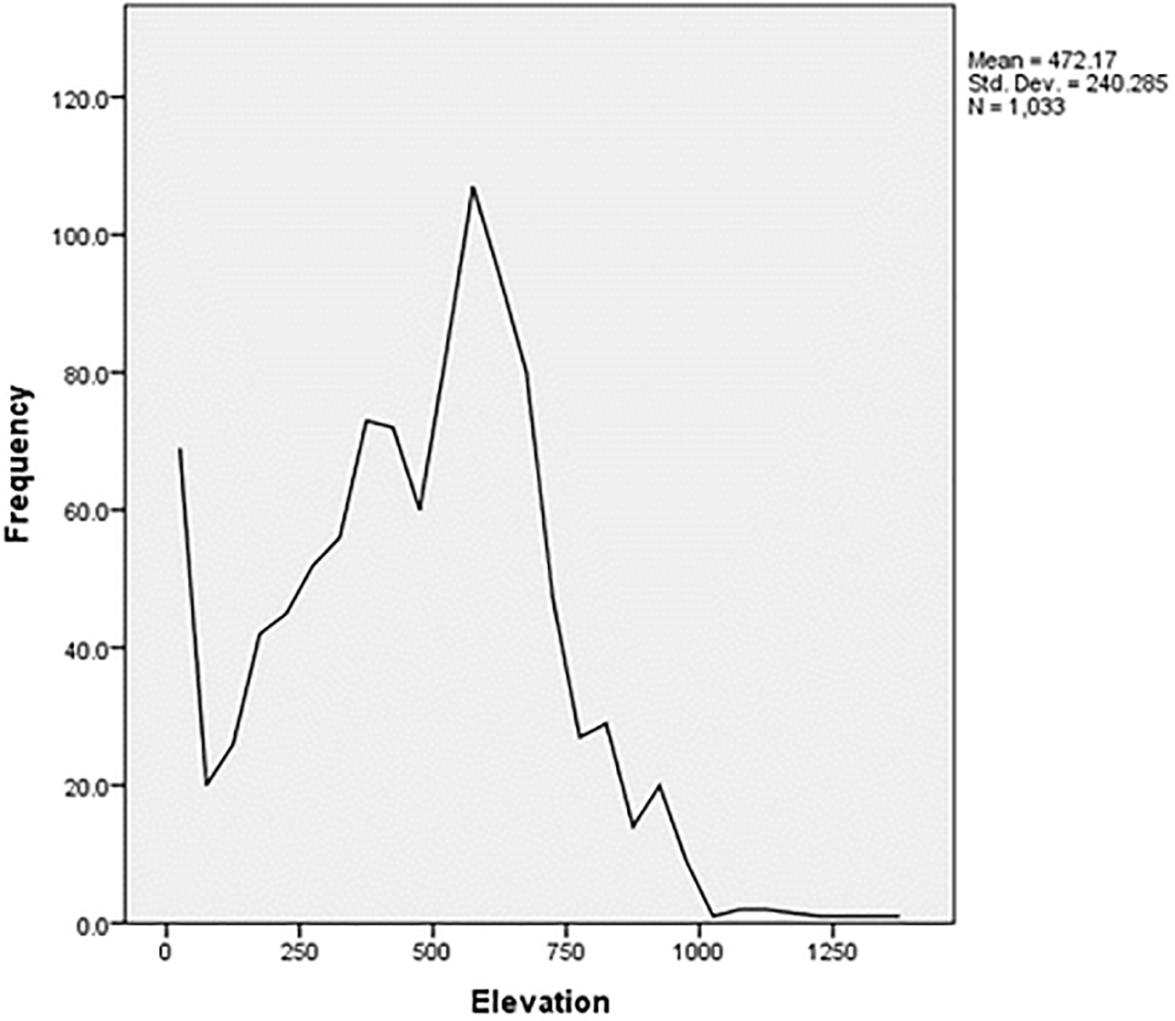
Frequency of Dubas bug infestations and elevation (Esri ArcGIS 10.3).

Elevation, geology (*p* < 0.1) and distance to the sea (*p* < 0.5) were found in high value significant clusters, while water type, slope, soil type and distance to the streams were found in low value significant clusters (Table 3). Dubas bug populations occurred with high frequency in the 251–500 m, and 501–750 m elevation classes, but occurred only occasionally in the 8–250 m (the smallest class), 751–1000 m, and 1001–1250 m (Fig 4). Dubas bug populations were shown to occur most frequently in areas of gravel and loam soils. Dubas bug infestations also occurred in areas of rocky soil, but in smaller numbers.

**Table 3:** G-statistic for Dubas bug infestations.

Variables	OGG	EGG	*p*-value	*z*-score	Sign
Water type	0.194605	0.215308	0.000000	-10.363497	Low-Cluster
Elevation	0.281037	0.215308	0.000000	18.368917	High-Cluster
Slope	0.225077	0.215308	0.186305	1.321590	Low-Cluster
Aspect	0.17328	0.000018	0.731915	-0.342579	Random
Soil type	0.187690	0.215308	0.000549	-3.455668	Low- Cluster
Geology	0.237637	0.215308	0.000007	4.496675	High-Cluster
Distance to Sea	0.292797	0.215308	0.000000	18.954813	High-Cluster
Distance to Streams	0.189344	0.215308	0.023924	-2.258354	Low-Cluster
Hillshade	0.215760	0.215308	0.586149	0.544425	Random

Of the 59 geological categories, we found that DB infestations occur most frequently on the Samail Ophiolites and the East Oman Ophiolite Complex (47-STH), which are unique to the Sultanate of Oman [44]. Infestations on shelf facies, volcanic rocks (Al-Akhdar mountain group; PTRAK) and alluvial deposits were also observed, but were less numerous.

Although the Moran’s *I* and G-statistic identified strong spatial patterns in the relationships between environmental variables and DB infestations, they only considered the distribution of a single variable in a single layer at a time. It is difficult to determine whether strong or weak relationships are becoming more or less spatially segregated. To fully model the environmental controls on DB infestations, multivariate statistical techniques were needed to integrate the correlations.

#### 3.2.4 Local Moran’s I statistics

**Fig 5:**
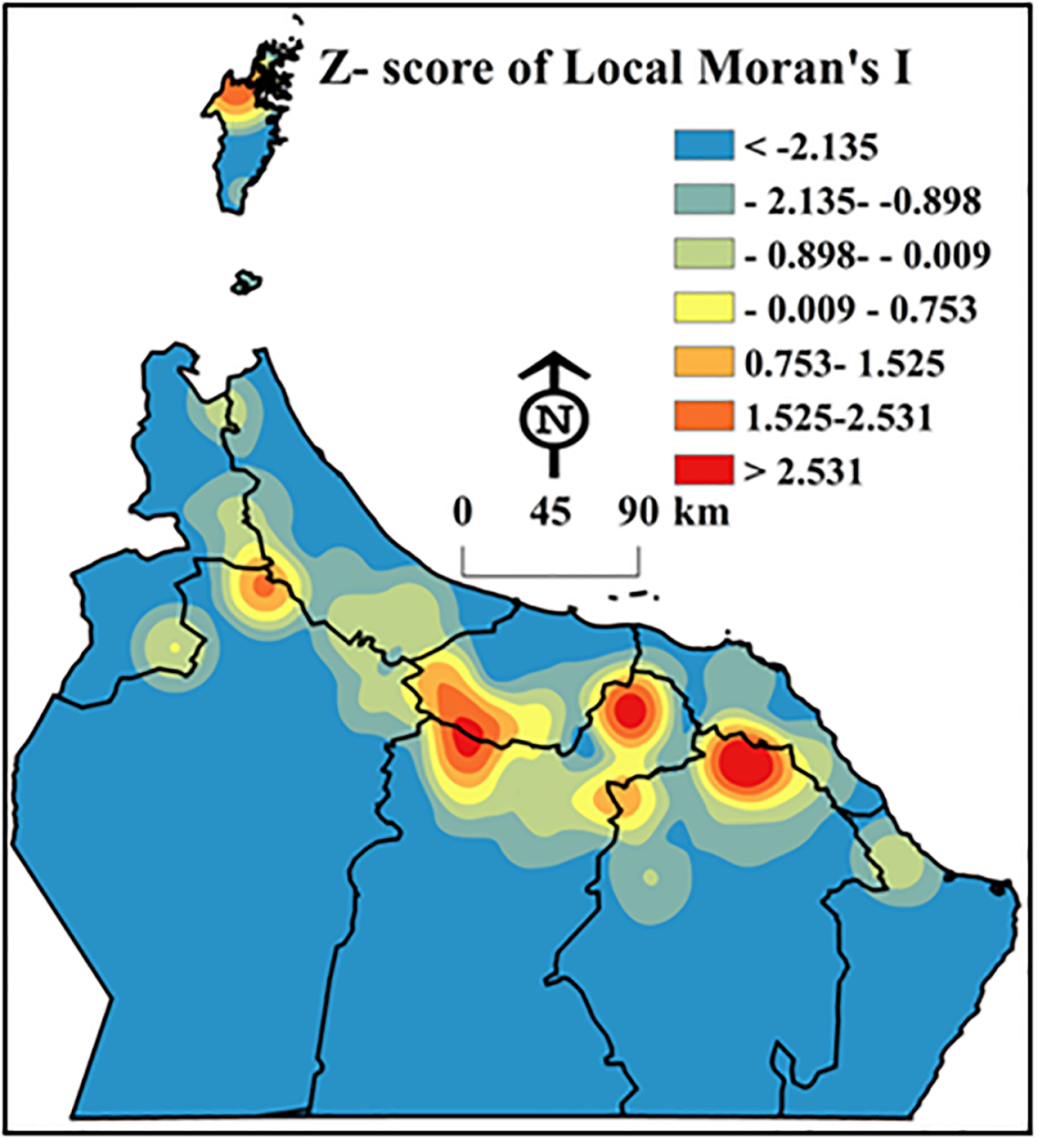
Surface map created from *z*-scores to present a generalized view of Dubas bug infestation hot and cold spots. Dark red areas denote a high infestation of the bugs that is significantly similar to its neighbours (at a confidence level of 0.01) (Esri ArcGIS 10.3).

By identifying hotspot and cold spot clusters for each variable, we were able to compare cluster locations in order to gain a better understanding of cluster control. Accurate “hotspot and cold spot” locations of DB infestations calculated from the statistic Local Moran’s I were mapped based on their *z*-score. The *z*-score values (> 2.58) indicated that the DB hotspots observed were statistically significant (Fig 5). Thus, these data were suitable for use in multivariate regression analysis to investigate the dependence of DB infestations on environmental variables (see Appendix A, S2 and S3 Files).

### 3.3 Modelling spatial relationships

#### 3.3.1 Exploratory regression

A global linear regression was used to build a model relationships between the independent variable (DB hotspot and cold spot infestations) and candidate environmental variables (Table 4). Positive significant relationships were found between DB infestations and elevation, geology, distance to streams (Table 5). In contrast, negative significant relationships were found between the hillshade and aspect factors of the DB infestations.

**Table 4:** Summary of residual normality (JB) and residual spatial autocorrelation (SA).

Variables	AdjR2 [a]	SA [b]	[c] AICc	JB [d]	VIF [e]	Model [f]
Elevation	0.012286	0.00	7501.396	0.00	1.98	+***
Slope	0.000426	0.00	7512.998	0.00	1.29	+*
Aspect	-0.000244	0.00	7513.649	0.00	1.13	_
Hillshade	0.001550	0.00	7511.904	0.00	1.17	_
Soil	0.008685	0.00	7504.934	0.00	1.19	+*
Geology	0.005026	0.00	7508.514	0.00	1.13	+**
Distance to streams	0.040466	0.00	7473.261	0.00	1.07	+***
Water	-0.000615	0.00	7514.010	0.00	1.25	+*
Distance to sea	-0.001012	0.00	7514.395	0.00	1.80	+*

^a.^ Min Adjusted *r*^2^ > 0.50

^b.^ Min spatial autocorrelation *p*-value > 0.10

^c.^ Akaike’s Information Criterion

^d.^ Min Jarque-Bera *p*-value 0.10

^e.^ Max VIF Value < 7.50

^f.^ Model variable signs are denoted by + and _; model variable significance is denoted by * = 0.10, ** = 0.05, *** = 0.01.

**Table 5:** Summary of variables significance.

Variables	Significant^a^	Positive^b^	Negative^c^
Elevation	100.00	100.00	0.00
Slope	0.00	76.07	23.93
Aspect	0.00	0.00	100.00
Hillshade	0.61	0.00	100.00
Soil	86	100.00	0.00
Geology	51.53	100.00	0.00
Distance to streams	100.00	100.00	0.00
Water	0.00	39.26	60.74
Distance to sea	31.90	45.40	31.90

^a.^ Percentage with a significant correlation

^b.^ Percentage with a positive correlation

^c.^ Percentage with a negative correlation

**Fig 6:**
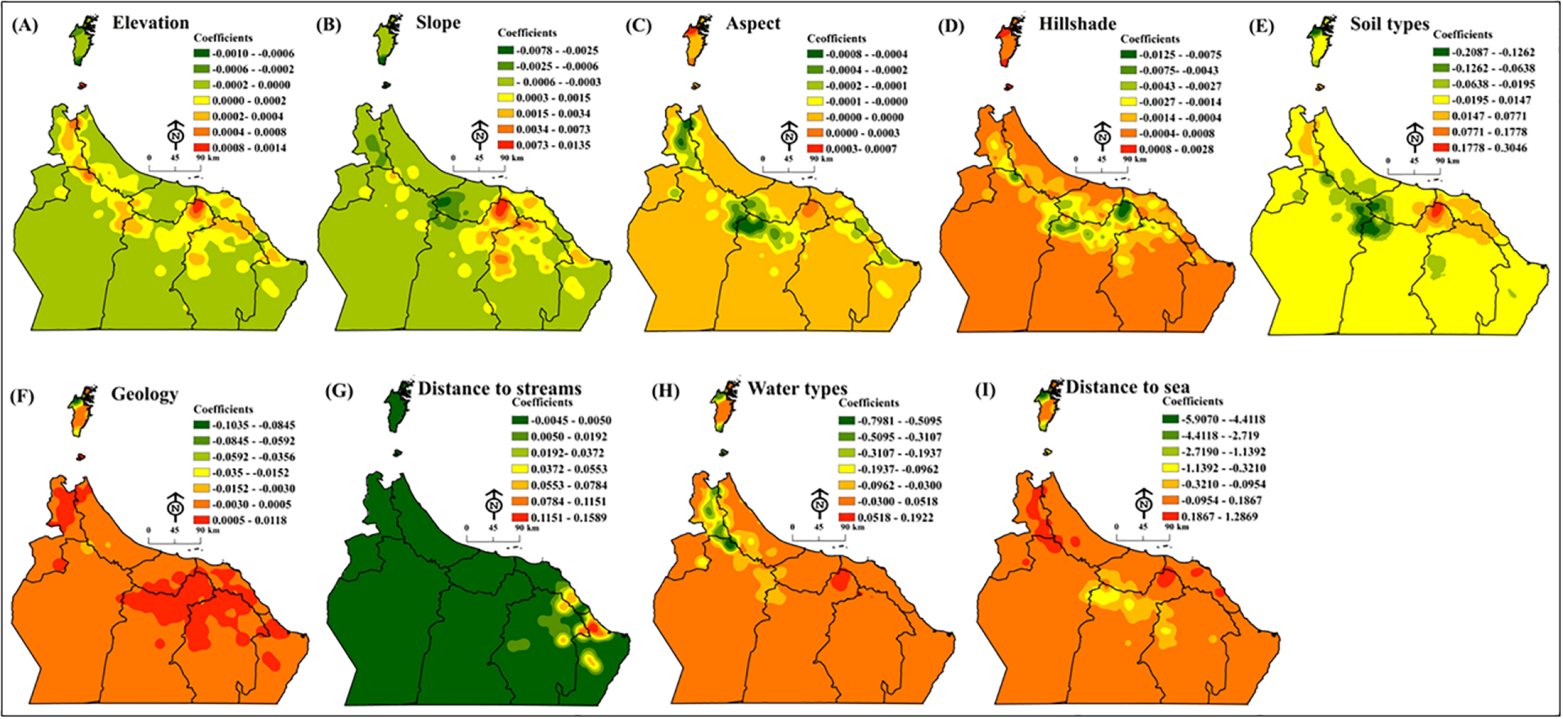
Geographically weighted regression (GWR) GWR optionally creates coefficient surface maps for each model explanatory variable reflecting variations in the modelled correlation, including: (A) elevation, (B) slope, (C) aspect, (D) hillshade, (E) soil types, (F) geology, (G) distance to streams, (H) water type, and (I) distance to the sea (Esri ArcGIS 10.3).

We created surface analysis maps of predictive values, *r*^2^ values, and standard residuals from GWR. These allowed us to investigate the combination of factors most conducive to the survival and spread of DBs. The coefficient surface maps created through GWR showed where the variable had the biggest impact of the regression across the study area, with strong relationship mapped with warm colours (orange to dark red) and relationship mapped weak with cold colours (yellow to dark green) (Fig 6).

**Fig 7:**
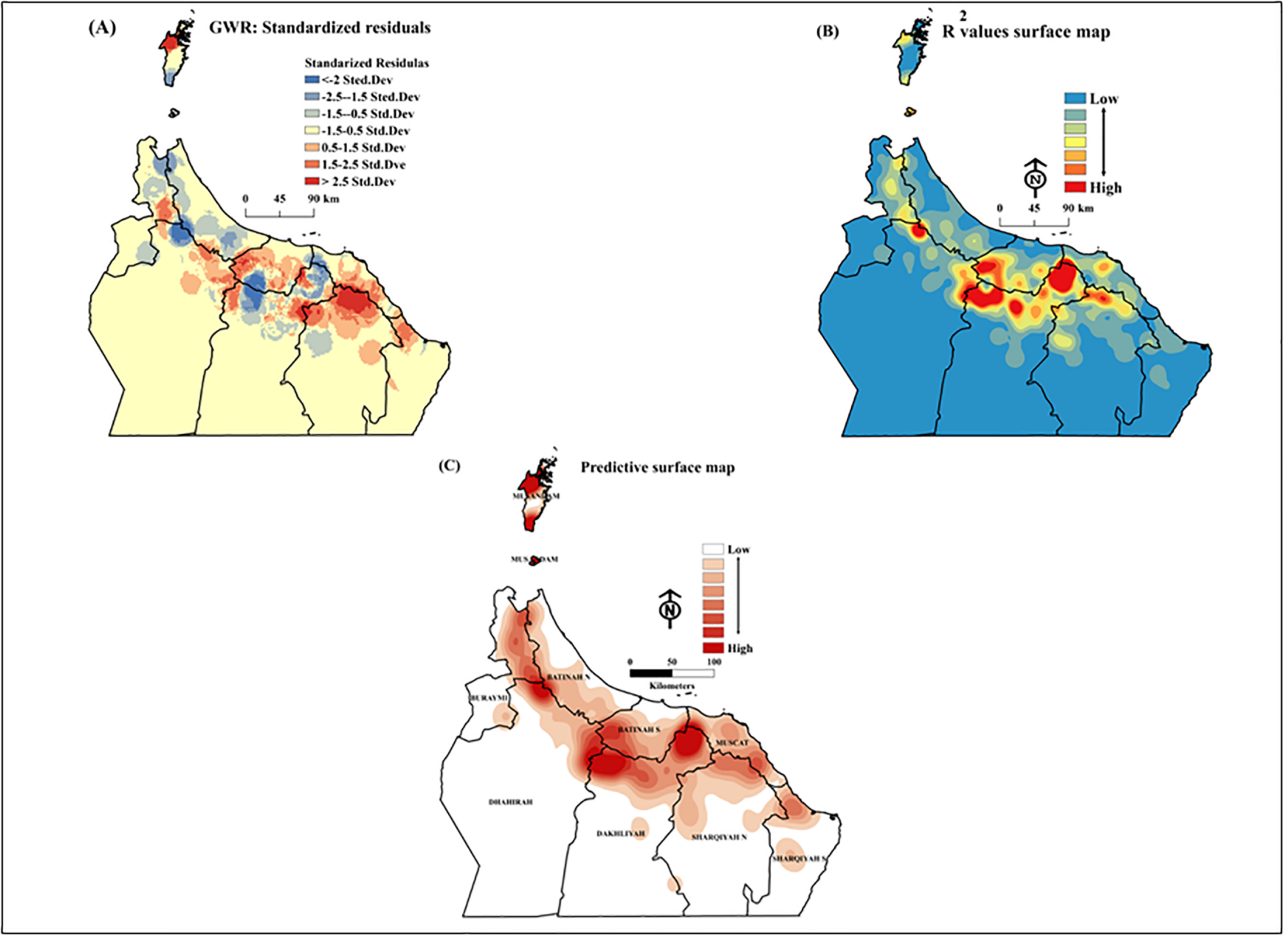
Geographically weighted regression (GWR) surface maps of: (A) standardized residuals, (B) locally weighed coefficients of determination (*r*^2^) between observed and fitted values, and (C) predictive data on possible future infestation areas (Esri ArcGIS 10.3).

The standardized residuals and distribution of locally weighed coefficients of determination (*r*^2^) between observed and fitted values showed which model had a higher proportion of dependent variable variance accounted for by the regression model (Fig 7).

The results of our analyses showed that certain variables impact more on DB survival (e.g., elevation, geology, and distance to streams). The infestations seem more severe in the mountains wadi biomes and less in the open plains (e.g. desert), and coastal areas away from mountains [33]. Furthermore, we observed that heavy infestations occur mostly along wadi where there is intensive and fresh water in Oman [45]. Wadi plains might also produce the quantity and quality of juices favoured by nymphs and adults of Dubas bug. Soil type and water type have had less impact of DB infestations. This is possible because soil permeability is related to the degree of water holding of soil and assuming that DB may have relationship with water in soil.

Hillshade and aspect are linked because both factors impact on the level of direct sunlight, which in turn impact on DB infestation rate. In particular, Dubas bugs avoid extreme temperature (high and low) and direct sunlight [16,46] as well as dry areas with disturbing wind [47,48]. Observations by the Sultan Qaboos University (SQU) in 2008 and 2009 indicated a mass migration of Dubas bugs from low elevation like date fruit gardens around Al-Jabal Al-Khader to high elevation [49]. Migration from wadies (dry rivers) to mountains (or vice versa), could reflect migration towards more suitable temperature, and the mechanism of transportation may be provided by the daytime rising convectional air currents [33]. The optimal temperature for the biological activities of DB is 30°C, while temperature below 0°C could affect their ability to survive [50]. We believe that other factors play a more significant role in determining the distribution and survival on DBs, and future studies will continue to focus on identifying these variables.

Based on our results, we applied GWR to construct predictive surface maps to identify potential areas for future Dubas bug infestations (Fig 7c). The areas of highest risk included Al-Dakhliyah North, Al-Sharqiyah North, Al-Batinah South, Al-Dhahirah to the north-east and north-west, respectively, and a small island in Musandam Governorate.

## 4. Summary and conclusions

Environmental variables are important for determining the distribution and survival of any species, whether plants or animals, and this includes the DB. Understanding the distribution and affinity of DBs to different environmental variables can play a significant role in the mapping, control, and management of infestations that will involve resource allocation (e.g., spray teams, field personnel).

Novel spatial analysis and geostatistical technologies have the ability to replace the use of insecticides in controlling DB population and maintaining the required environmental balance. Elevation, slope, geology, soil type, water type and distance to streams were all found to be associated with increased DB infestations in northern Oman. By incorporating the spatial influences into the analysis of our results, we were able to better reflect real-world relationships and to build predictive models of future infestations. Furthermore, we believe that additional variables such as climatological conditions and human practices might contribute to influencing the distribution and survival of the DB in the north of Oman. These variables will be the subjects of our study in the immediate future.

Moreover, we believe the results of our study will provide guidance and ideas to policy makers on how to adopt more appropriate and more specific control and prevention strategies of DBs in specific areas. We propose a simple and inexpensive technique, based on case notification data, for early warning, categorization and identifications of at-risk area that can be incorporated into routine monitoring by the agriculture authorities. The method prevents DB prevalence, helps mentoring and identifying DB adults and nymphs shelters for the elimination of DB breeding sites such as farms close to mountains and streams. In addition, reducing the cultivation of palm crop nearby mountains zone and finding open plains exposed the sunlight and air between palm trees. Dubas bug detected in relatively unpopulated area that should also be occasionally monitored for the presence or absence of the bugs.

## Supporting information

S1 File Name of locations and geographic coordinates for the data set. (PDF)

S2 File Local Moran’s *I* statictic, A spatial analyset results. (PDF)

S3 File Hotspot analysis (Getis-Ord Gi*). (PDF)
