# Supplementary material for: Impact of environmental variables on Dubas bug infestation rate: A case study from the Sultanate of Oman
Source: PLoS One. 2017 May 30;12(5):e0178109. doi: 10.1371/journal.pone.0178109 (PMC5448759; doi:10.1371/journal.pone.0178109)
Supplement: S1 File — (PDF) [file pone.0178109.s001.pdf]

ID	Long_X	Lat_Y	Name of Location	DB-DENSITY	DB-PRESENCE
1	57.75036	22.86826	IZKI	High	1
2	57.76747	22.91831	IZKI	High	1
3	57.78356	22.93436	IZKI	High	1
4	57.29136	23.10534	AL HAMRA	No	0
5	58.12739	23.44414	BID BID	No	0
6	57.13364	23.03436	BAHLA	High	1
7	57.77762	22.99806	IZKI	High	1
8	57.26240	23.06587	AL HAMRA	No	0
9	58.14393	23.39373	BID BID	No	0
10	58.15710	23.37734	BID BID	No	0
11	56.26197	25.66752	DABA	Low	1
12	57.30123	23.09629	AL HAMRA	No	0
13	57.65398	22.79913	MANAH	No	0
14	57.63386	23.18367	AL RUSTAQ	High	1
15	57.54900	22.82616	MANAH	Low	1
16	57.61801	23.18699	AL AWABI	High	1
17	57.85097	23.15150	SAMIL	Mid	1
18	57.85025	23.15011	SAMIL	No	0
19	58.07483	23.23188	SAMIL	Mid	1
20	57.55275	23.22192	AL AWABI	High	1
21	57.52727	23.30828	AL RUSTAQ	No	0
22	57.62736	22.74464	MANAH	No	0
23	57.55794	22.71519	MANAH	No	0
24	56.41709	23.88434	YANQAL	High	1
25	56.40970	23.87365	YANQAL	No	0
26	57.58478	22.78910	MANAH	Mid	1
27	56.37529	23.86103	YANQAL	No	0
28	57.53168	22.94384	NIZWA	No	0
29	57.98446	23.30126	SAMIL	High	1
30	58.01809	23.31695	SAMIL	High	1
31	57.96226	22.85302	IZKI	No	0
32	57.67315	22.98990	NIZWA	Mid	1
33	58.07358	23.31431	SAMIL	High	1
34	57.92186	23.28880	SAMIL	No	0
35	58.04045	23.28436	SAMIL	High	1
36	58.05973	23.29807	SAMIL	High	1
37	57.11821	23.41274	AL RUSTAQ	No	0
38	57.15238	23.42275	AL RUSTAQ	High	1
39	56.34522	23.93233	YANQAL	No	0
40	57.75117	22.93336	IZKI	High	1
41	57.76711	22.93386	IZKI	High	1
42	57.76686	22.91828	IZKI	No	0
43	58.05189	23.22750	SAMIL	Mid	1
44	57.12775	23.06975	BAHLA	High	1
45	57.31183	23.09935	AL HAMRA	High	1
46	57.76764	22.93483	IZKI	High	1
47	57.78406	22.98450	IZKI	High	1
48	57.53598	22.94912	NIZWA	High	1
49	57.54336	22.95762	NIZWA	High	1
50	57.31240	23.13933	AL HAMRA	High	1
51	57.20078	23.15153	AL HAMRA	High	1
52	56.36473	23.83814	YANQAL	No	0
53	58.01719	23.35061	SAMIL	High	1
54	58.02094	23.35364	SAMIL	High	1
55	58.34249	23.08861	BID BID	High	1
56	58.35201	23.09860	BID BID	High	1
57	57.46589	22.85540	NIZWA	Low	1
58	56.17116	24.03167	AL BURAYMI	No	0
59	58.81460	23.17324	QURAYYAT	High	1
60	58.81778	23.13450	QURAYYAT	High	1
61	57.46690	22.85090	NIZWA	Low	1

62	58.97456	23.20562	QURAYYAT	High	1
63	58.03692	23.35679	SAMIL	High	1
64	57.45808	23.05409	NIZWA	High	1
65	57.53471	23.02132	NIZWA	Mid	1
66	58.21781	23.31825	BID BID	No	0
67	57.90092	23.16786	SAMIL	High	1
68	57.98431	23.01722	SAMIL	Mid	1
69	58.01743	23.00163	SAMIL	Mid	1
70	58.01670	23.01781	SAMIL	Mid	1
71	57.96817	23.01794	SAMIL	Mid	1
72	57.91210	22.96455	IZKI	High	1
73	57.91814	22.95111	IZKI	High	1
74	57.89637	22.94952	IZKI	High	1
75	57.92952	22.89239	IZKI	High	1
76	56.11761	24.30025	MAHADAH	High	1
77	56.11739	24.30106	MAHADAH	High	1
78	57.60293	22.79370	MANAH	High	1
79	56.81948	23.08230	IBRI	High	1
80	56.11728	24.30164	MAHADAH	High	1
81	56.03478	24.36683	MAHADAH	No	0
82	57.52365	22.93433	NIZWA	High	1
83	56.90863	23.16862	IBRI	Low	1
84	57.53344	22.90028	NIZWA	High	1
85	56.96160	23.21078	IBRI	High	1
86	57.01128	23.21538	IBRI	High	1
87	57.85108	23.08497	SAMIL	High	1
88	57.81750	23.10083	SAMIL	High	1
89	57.97347	23.37204	SAMIL	High	1
90	57.95021	23.32584	SAMIL	High	1
91	57.28070	23.11893	AL HAMRA	Low	1
92	56.35822	24.19927	AL BURAYMI	No	0
93	57.85011	23.15142	SAMIL	High	1
94	56.42017	23.93425	YANQAL	Low	1
95	56.35055	23.93678	YANQAL	Mid	1
96	58.10579	23.36156	BID BID	Low	1
97	58.10162	23.41022	BID BID	High	1
98	58.12394	23.33438	BID BID	Low	1
99	57.77345	23.39994	IZKI	Low	1
100	57.04418	23.23055	IBRI	High	1
101	56.40494	23.85879	YANQAL	Mid	1
102	58.08279	23.20872	BID BID	Low	1
103	57.02454	23.21350	IBRI	Low	1
104	58.08254	23.20983	BID BID	Low	1
105	57.04491	23.25285	IBRI	High	1
106	58.06825	23.23442	BID BID	Low	1
107	58.10387	23.25126	BID BID	High	1
108	58.13254	23.22160	BID BID	High	1
109	56.89599	23.13874	IBRI	Low	1
110	56.25608	25.63267	MADHA	Low	1
111	56.31971	25.27300	MADHA	No	0
112	56.31058	25.28550	MADHA	No	0
114	57.07033	23.10676	IBRI	High	1
115	57.80100	22.78383	IZKI	High	1
116	58.01392	22.88423	IZKI	High	1
117	56.10064	26.06731	BUKHA	High	1
118	56.13055	26.10875	BUKHA	High	1
119	56.13079	26.11087	BUKHA	High	1
121	56.17297	26.15904	BUKHA	High	1
122	56.15829	26.13988	BUKHA	High	1
123	58.10599	23.43614	BID BID	Low	1
124	58.11127	23.43492	BID BID	High	1
125	58.09668	23.42508	BID BID	High	1

126	56.24076	25.62110	DABA	No	0
128	56.23166	24.20608	AL BURAYMI	Mid	1
129	55.96778	23.61703	AS SUNAYNAH	No	0
130	55.96081	23.59090	AS SUNAYNAH	No	0
131	56.06720	24.46428	MAHADAH	Mid	1
132	55.97164	24.49859	MAHADAH	High	1
133	57.00410	23.05992	IBRI	High	1
134	56.16043	24.03723	AL BURAYMI	High	1
135	56.21308	24.03287	AL BURAYMI	Mid	1
136	56.96689	23.07136	IBRI	High	1
137	58.10150	23.47356	BID BID	High	1
138	58.09237	23.41076	BID BID	High	1
139	56.25272	26.19172	KHASAB	High	1
140	56.24468	26.18887	KHASAB	High	1
141	56.24851	26.19016	KHASAB	High	1
142	56.22248	26.18815	KHASAB	High	1
143	56.24585	26.19499	KHASAB	High	1
144	57.08079	23.13294	IBRI	High	1
145	56.05706	24.45629	MAHADAH	Mid	1
146	57.08786	23.14765	IBRI	High	1
147	58.13492	23.41733	BID BID	Low	1
148	57.40164	23.18364	AL RUSTAQ	Low	1
149	56.16753	24.75111	MAHADAH	Low	1
150	56.18271	24.71655	MAHADAH	High	1
151	56.16569	24.71322	MAHADAH	No	0
152	56.16548	24.69254	MAHADAH	No	0
153	56.06327	24.61444	MAHADAH	High	1
154	57.30877	23.36437	AL RUSTAQ	Low	1
155	57.32959	23.29050	AL RUSTAQ	Low	1
156	57.31768	23.24864	AL RUSTAQ	Low	1
157	57.33705	23.25723	AL RUSTAQ	Low	1
158	57.31769	23.22164	AL RUSTAQ	Low	1
159	57.33276	23.22289	AL RUSTAQ	Low	1
160	57.31592	23.38781	AL RUSTAQ	Low	1
161	56.29384	25.26773	MADHA	No	0
162	56.21451	24.13659	AL BURAYMI	High	1
163	58.55417	23.43320	QURAYYAT	High	1
164	58.66697	23.50122	AS SEEB	Mid	1
165	58.51177	22.71734	IBRA	Mid	1
166	57.08600	23.45810	AL RUSTAQ	Mid	1
167	57.83953	23.39152	NAKHAL	Mid	1
168	57.74036	23.15662	NAKHAL	Mid	1
169	57.81029	23.45453	WADI AL MAAWIL	High	1
170	57.44244	23.21791	AL RUSTAQ	High	1
171	58.31250	23.15601	BID BID	High	1
172	58.31877	23.18125	DAMA WAAT TAIYIN	High	1
173	58.47609	23.11566	DAMA WAAT TAIYIN	High	1
174	58.46162	23.12349	DAMA WAAT TAIYIN	High	1
175	58.60153	23.08581	DAMA WAAT TAIYIN	High	1
176	58.40092	23.13749	DAMA WAAT TAIYIN	High	1
177	58.56733	23.08417	DAMA WAAT TAIYIN	High	1
178	58.58419	23.11667	DAMA WAAT TAIYIN	High	1
179	58.54179	23.11756	DAMA WAAT TAIYIN	High	1
180	58.53433	23.11697	DAMA WAAT TAIYIN	High	1
181	58.56920	23.10206	DAMA WAAT TAIYIN	High	1
182	58.51843	23.11393	IBRA	High	1
183	58.39167	22.96822	IBRA	High	1
184	58.55075	22.96722	DAMA WAAT TAIYIN	High	1
185	58.83052	22.94658	DAMA WAAT TAIYIN	High	1
186	58.88255	22.91493	DAMA WAAT TAIYIN	High	1
187	58.51468	23.04545	DAMA WAAT TAIYIN	High	1
188	58.68676	23.04317	DAMA WAAT TAIYIN	High	1

189	58.66244	23.04614	DAMA WAAT TAIYIN	High	1
190	58.64263	23.04811	DAMA WAAT TAIYIN	High	1
191	58.79211	22.98144	DAMA WAAT TAIYIN	High	1
192	58.78068	22.99159	DAMA WAAT TAIYIN	High	1
193	58.74039	23.02034	DAMA WAAT TAIYIN	High	1
194	58.35611	23.10890	BID BID	High	1
195	58.02599	22.89784	AL MUDAYBI	High	1
196	58.05579	22.92321	AL MUDAYBI	High	1
197	58.07752	22.93237	AL MUDAYBI	High	1
198	58.28478	22.98375	AL MUDAYBI	High	1
199	58.28486	22.96719	AL MUDAYBI	High	1
200	58.21769	22.88383	AL MUDAYBI	High	1
201	58.01047	23.31248	SAMIL	High	1
202	58.00111	23.31030	SAMIL	High	1
203	58.02548	23.30944	SAMIL	High	1
204	57.63840	23.35602	NAKHAL	High	1
205	57.57332	23.18781	AL AWABI	High	1
206	57.56552	23.18781	AL AWABI	High	1
207	57.12853	23.42015	AL RUSTAQ	High	1
208	57.11678	23.41167	AL RUSTAQ	High	1
209	57.12146	23.41574	AL RUSTAQ	High	1
210	57.13149	23.42066	AL RUSTAQ	High	1
211	57.16529	23.42364	AL RUSTAQ	High	1
212	57.17187	23.40847	AL RUSTAQ	High	1
213	57.29790	23.42721	AL RUSTAQ	High	1
214	57.12802	23.05079	BAHLA	High	1
215	57.13174	23.04141	BAHLA	High	1
216	57.16192	22.98820	BAHLA	High	1
217	56.11267	24.65334	MAHADAH	High	1
218	59.26968	22.64668	SUR	High	1
219	59.24756	22.64718	SUR	High	1
220	59.22029	22.65713	SUR	High	1
221	58.79885	22.42884	BIDIYAH	High	1
222	58.32814	23.48049	BAWSHAR	High	1
223	58.91080	23.06001	QURAYYAT	High	1
224	58.92720	23.04924	QURAYYAT	High	1
225	58.93493	23.04062	QURAYYAT	High	1
226	58.76998	22.88552	AL QABIL	High	1
227	57.06254	23.22870	IBRI	High	1
228	57.05028	23.23356	IBRI	High	1
229	56.89397	23.28446	IBRI	High	1
230	56.92149	23.28232	IBRI	High	1
231	56.99304	23.40228	IBRI	High	1
232	56.63472	23.56822	YANQAL	High	1
233	56.63486	23.63492	YANQAL	High	1
234	56.92150	23.38962	IBRI	High	1
235	56.88854	23.40856	IBRI	High	1
236	56.88266	23.37291	IBRI	High	1
237	56.82932	23.42600	IBRI	High	1
238	56.78358	23.55025	IBRI	High	1
239	56.80622	23.63552	IBRI	High	1
240	56.75441	23.68888	SAHAM	High	1
241	56.74983	23.70482	SAHAM	High	1
242	56.48422	23.50108	DANK	High	1
243	56.11686	24.08942	AL BURAYMI	High	1
244	56.20106	24.12876	AL BURAYMI	High	1
245	56.25476	24.09261	AL BURAYMI	High	1
246	56.26367	24.19535	AL BURAYMI	High	1
247	57.29232	23.09029	AL HAMRA	No	0
248	58.13369	23.43392	BID BID	High	1
249	58.13417	23.43736	BID BID	High	1
250	57.51814	22.38378	ADAM	High	1

251	57.77905	23.00615	IZKI	High	1
252	58.14856	23.41749	BID BID	High	1
253	58.15081	23.38002	BID BID	No	0
254	56.41913	25.94139	DABA	Low	1
255	57.28838	23.11092	AL HAMRA	No	0
256	57.58555	22.79513	MANAH	Low	1
257	57.52688	22.93693	NIZWA	Mid	1
258	57.60835	23.18209	AL AWABI	High	1
259	58.07989	23.22803	SAMIL	Mid	1
260	57.55516	23.21634	AL AWABI	High	1
261	57.53345	23.31102	AL RUSTAQ	No	0
262	55.95033	23.61703	AS SUNAYNAH	Low	1
263	56.41472	23.88379	YANQAL	High	1
264	56.40737	23.85904	YANQAL	No	0
265	56.36772	23.86817	YANQAL	High	0
266	57.52751	22.92748	NIZWA	No	0
267	57.67053	22.98969	NIZWA	Mid	1
268	58.08175	23.31881	SAMIL	High	1
269	57.97486	23.29008	SAMIL	High	1
270	58.03367	23.27793	SAMIL	High	1
271	58.03365	23.30452	SAMIL	High	1
272	57.12356	23.42431	AL RUSTAQ	No	0
273	57.15113	23.42239	AL RUSTAQ	High	1
274	56.35012	23.94280	YANQAL	No	0
275	59.27752	22.09864	L KAMIL WA AL WAF	Low	1
276	57.12657	23.07188	BAHLA	High	1
277	57.28988	23.10809	AL HAMRA	High	1
278	57.55231	22.96536	NIZWA	High	1
279	57.53414	22.93472	NIZWA	High	1
280	57.31714	23.15011	AL HAMRA	High	1
281	56.40554	23.85492	YANQAL	High	0
282	58.01940	23.35233	SAMIL	High	1
283	58.31900	23.11828	BID BID	High	1
284	58.35470	23.10686	BID BID	High	1
285	57.52693	22.86318	NIZWA	Mid	1
286	57.60078	22.80125	MANAH	High	1
287	56.17622	24.04630	AL BURAYMI	No	0
288	58.83122	23.14806	QURAYYAT	High	1
289	58.92145	23.17384	QURAYYAT	High	1
290	58.04815	23.35707	SAMIL	High	1
291	57.46037	23.05447	NIZWA	High	1
292	57.53703	23.01979	NIZWA	Mid	1
293	58.21767	23.30097	BID BID	High	1
294	57.91733	22.97644	IZKI	High	1
295	57.88494	22.95047	IZKI	High	1
296	56.82719	23.09118	IBRI	High	1
297	57.53138	22.95039	NIZWA	High	1
298	56.91219	23.17059	IBRI	Low	1
299	56.95150	23.20128	IBRI	High	1
300	57.01728	23.21780	IBRI	High	1
301	57.86733	23.21686	SAMIL	High	1
302	57.96747	23.36728	SAMIL	High	1
303	57.95233	23.32546	SAMIL	High	1
304	57.28442	23.11507	AL HAMRA	Low	1
306	56.48299	23.99014	YANQAL	High	1
307	56.34841	23.93335	YANQAL	High	1
308	58.10000	23.38176	BID BID	High	1
309	58.10781	23.36763	BID BID	High	1
310	58.12296	23.33176	BID BID	High	1
311	57.82488	23.38215	IZKI	Low	1
312	57.85019	22.78353	IZKI	High	1
313	57.01444	23.21419	IBRI	High	1

314	56.41430	23.84665	YANQAL	Mid	1
315	58.06808	23.22849	BID BID	Low	1
316	58.07747	23.21761	BID BID	Low	1
317	57.04562	23.24934	IBRI	High	1
318	58.10216	23.24989	BID BID	High	1
319	58.13640	23.22153	BID BID	High	1
320	56.89536	23.13435	IBRI	High	1
321	56.26814	25.63353	MADHA	Low	1
322	56.31852	25.27746	MADHA	No	0
323	56.31514	25.28119	MADHA	No	0
324	56.32579	25.28196	MADHA	No	0
325	57.05098	23.10255	IBRI	High	1
327	56.15142	26.13497	BUKHA	High	1
328	58.11766	23.43000	BID BID	Low	1
329	58.09593	23.44182	BID BID	High	1
330	58.09721	23.44269	BID BID	High	1
331	56.25418	25.61932	DABA	No	0
333	55.95981	24.22177	AL BURAYMI	Mid	1
334	55.94639	23.61332	AS SUNAYNAH	No	0
335	56.99232	23.05718	IBRI	High	1
336	56.15101	24.03473	AL BURAYMI	High	1
337	56.21660	24.03210	AL BURAYMI	High	1
338	56.96951	23.06434	IBRI	High	1
339	58.10440	23.46414	BID BID	High	1
340	58.12479	23.40749	BID BID	High	1
341	56.25658	26.18526	KHASAB	High	1
342	56.25073	26.18766	KHASAB	High	1
343	57.08960	23.13290	IBRI	High	1
344	56.05708	24.45006	MAHADAH	High	1
345	57.08697	23.14001	IBRI	High	1
346	56.08361	24.36731	MAHADAH	High	1
347	58.13378	23.41689	BID BID	High	1
348	56.18488	24.71885	MAHADAH	High	1
349	56.18234	24.70287	MAHADAH	No	0
350	56.16988	24.69236	MAHADAH	No	0
351	57.31027	23.36312	AL RUSTAQ	Low	1
352	57.32987	23.28843	AL RUSTAQ	Low	1
353	57.32048	23.22268	AL RUSTAQ	Low	1
354	57.33478	23.22135	AL RUSTAQ	Low	1
355	57.30952	23.38540	AL RUSTAQ	Low	1
357	56.21689	24.13391	AL BURAYMI	High	1
358	58.74817	23.26978	QURAYYAT	High	1
359	57.00643	23.49253	AL RUSTAQ	Mid	1
360	57.84164	23.39631	NAKHAL	Mid	1
361	57.80927	23.44684	WADI AL MAAWIL	High	1
362	57.40125	23.21739	AL RUSTAQ	High	1
363	57.44249	23.21648	AL RUSTAQ	High	1
364	58.31434	23.15292	BID BID	High	1
365	58.35042	23.18381	DAMA WAAT TAIYIN	High	1
366	58.38478	23.13372	DAMA WAAT TAIYIN	High	1
367	58.45474	23.10216	DAMA WAAT TAIYIN	High	1
368	58.42390	23.14312	DAMA WAAT TAIYIN	High	1
369	58.41463	23.13992	DAMA WAAT TAIYIN	High	1
370	58.56035	23.10657	DAMA WAAT TAIYIN	High	1
371	58.50924	23.11505	DAMA WAAT TAIYIN	High	1
372	58.57038	23.09787	IBRA	High	1
373	58.51758	22.96803	DAMA WAAT TAIYIN	High	1
374	58.56692	22.98400	DAMA WAAT TAIYIN	High	1
375	58.82686	22.94586	DAMA WAAT TAIYIN	High	1
376	58.87703	22.90122	DAMA WAAT TAIYIN	High	1
377	58.60156	23.06711	DAMA WAAT TAIYIN	High	1
378	58.57022	23.07486	DAMA WAAT TAIYIN	High	1

379	58.69019	23.04546	DAMA WAAT TAIYIN	High	1
380	58.61690	23.05285	DAMA WAAT TAIYIN	High	1
381	58.62536	23.04711	DAMA WAAT TAIYIN	High	1
382	58.78925	22.99153	DAMA WAAT TAIYIN	High	1
383	58.79099	22.98513	DAMA WAAT TAIYIN	High	1
384	58.74410	23.01901	DAMA WAAT TAIYIN	High	1
385	58.36706	23.11758	BID BID	High	1
386	58.05008	22.90156	AL MUDAYBI	High	1
387	58.05103	22.91797	AL MUDAYBI	High	1
388	58.06797	22.91828	AL MUDAYBI	High	1
389	58.06753	22.91667	AL MUDAYBI	High	1
390	58.17332	22.84717	AL MUDAYBI	High	1
391	58.16549	22.82771	AL MUDAYBI	High	1
392	58.13367	22.80014	AL MUDAYBI	High	1
393	58.04188	23.31362	SAMIL	High	1
394	57.91781	23.16683	SAMIL	High	1
395	58.03609	23.31015	SAMIL	High	1
396	58.04254	23.30180	SAMIL	High	1
397	57.66764	23.46711	NAKHAL	High	1
398	57.63278	23.35409	NAKHAL	High	1
399	57.63087	23.18602	AL AWABI	High	1
400	57.67869	23.20164	AL AWABI	High	1
401	57.11461	23.44877	AL RUSTAQ	High	1
402	57.12035	23.41488	AL RUSTAQ	High	1
403	57.11961	23.41383	AL RUSTAQ	High	1
404	57.13973	23.42190	AL RUSTAQ	High	1
405	57.18677	23.37594	AL RUSTAQ	High	1
406	57.17705	23.40404	AL RUSTAQ	High	1
407	57.43244	23.42321	AL RUSTAQ	High	1
408	57.13512	23.04190	BAHLA	High	1
409	57.14095	22.99109	BAHLA	High	1
410	57.16010	22.98763	BAHLA	High	1
411	56.11148	24.64780	MAHADAH	High	1
412	59.32155	22.55330	SUR	High	1
413	59.26179	22.64692	SUR	High	1
414	59.28732	22.64256	SUR	High	1
415	59.08393	22.60402	WADI BANI KHALID	High	1
416	59.10139	22.56700	WADI BANI KHALID	High	1
417	59.11797	22.55033	WADI BANI KHALID	High	1
418	59.17469	22.12970	ALAN BANI BU HAS	High	1
419	58.86222	22.44769	BIDIYAH	High	1
420	58.81708	22.44212	BIDIYAH	High	1
421	58.32523	23.47623	BAWSHAR	High	1
422	58.55056	23.25017	AL AMRAT	High	1
423	58.70114	23.23414	QURAYYAT	High	1
424	58.68956	22.73316	AL QABIL	High	1
425	58.75679	22.87849	AL QABIL	High	1
426	58.74060	22.89265	AL QABIL	High	1
427	58.65496	22.72359	AL QABIL	High	1
428	57.06583	23.22942	IBRI	High	1
429	56.89423	23.27864	IBRI	High	1
430	56.93120	23.27890	IBRI	High	1
431	56.98638	23.39704	IBRI	High	1
432	56.91585	23.39468	IBRI	High	1
433	56.88818	23.40250	IBRI	High	1
434	56.86123	23.40025	IBRI	High	1
435	56.83830	23.42112	IBRI	High	1
436	56.80217	23.65684	IBRI	High	1
437	56.77672	23.66732	SAHAM	High	1
438	56.74768	23.70565	SAHAM	High	1
439	56.01064	24.14938	AL BURAYMI	High	1
440	56.24591	24.09378	AL BURAYMI	High	1

441	56.26333	24.20111	AL BURAYMI	High	1
442	56.03347	24.40017	MAHADAH	High	1
443	59.28762	22.58981	SUR	High	1
444	59.33669	22.51280	SUR	High	1
445	59.37001	22.69924	SUR	High	1
446	59.25136	22.81744	SUR	High	1
447	59.23456	22.83397	SUR	High	1
448	57.12042	23.63446	SAHAM	High	1
449	57.16111	23.65533	SAHAM	High	1
450	57.44464	23.48631	AL RUSTAQ	High	1
451	57.33531	23.56409	AL RUSTAQ	High	1
452	57.33092	23.52408	AL RUSTAQ	High	1
453	57.22486	23.52001	AL RUSTAQ	High	1
454	57.19328	23.47782	AL RUSTAQ	High	1
455	57.18100	23.48690	AL RUSTAQ	High	1
456	57.30301	23.42767	AL RUSTAQ	High	1
457	57.44561	23.55238	AL RUSTAQ	High	1
458	57.18852	23.50374	AL RUSTAQ	High	1
459	57.21767	23.54501	AL RUSTAQ	High	1
460	57.58597	23.31763	AL AWABI	High	1
461	57.58278	23.31806	AL AWABI	High	1
462	56.74022	23.43369	IBRI	High	1
463	56.53988	23.60128	YANQAL	High	1
464	56.50884	23.69692	YANQAL	High	1
465	56.55744	23.68541	YANQAL	High	1
466	56.19395	23.93446	DANK	High	1
467	56.23199	23.92548	DANK	High	1
468	56.24772	23.93626	DANK	High	1
469	58.51764	23.40058	AL AMRAT	High	1
470	58.55164	23.43394	AL AMRAT	High	1
471	58.56349	23.43419	AL AMRAT	High	1
472	58.43333	22.86681	IBRA	High	1
473	56.16744	24.01825	AL BURAYMI	No	0
474	58.82781	23.14487	QURAYYAT	Mid	1
475	58.18431	23.62887	AS SEEB	Mid	1
476	58.41979	23.58053	AS SEEB	Mid	1
477	58.98598	23.19555	QURAYYAT	Mid	1
478	58.61890	23.50888	QURAYYAT	Mid	1
479	58.48505	23.45016	QURAYYAT	No	0
480	58.68461	23.21717	QURAYYAT	Mid	1
481	58.78172	23.25562	QURAYYAT	No	0
482	58.98118	23.05549	QURAYYAT	No	0
483	58.98089	23.04478	QURAYYAT	Mid	1
484	58.91800	23.16781	QURAYYAT	Low	1
485	56.31796	25.27206	MADHA	No	0
486	56.31843	25.28041	MADHA	No	0
487	56.29120	24.70144	SHINAS	Low	1
488	57.10047	23.83453	AL KHABURAH	No	0
489	57.11722	23.81728	AL KHABURAH	Low	1
490	56.28347	24.62879	SHINAS	No	0
491	57.07208	23.68141	AL KHABURAH	Low	1
492	57.04629	23.73941	AL KHABURAH	Low	1
493	57.03709	23.72268	AL KHABURAH	Low	1
494	57.01659	23.76979	AL KHABURAH	No	0
495	56.97374	23.75045	AL KHABURAH	No	0
496	56.69510	23.92013	AL KHABURAH	Low	1
497	56.63100	23.83436	AL KHABURAH	Low	1
498	56.78298	24.04768	SAHAM	No	0
499	56.68393	23.99937	SAHAM	Low	1
500	56.64807	23.98599	SAHAM	Low	1
501	56.78254	23.84408	AL KHABURAH	Low	1
502	56.68371	23.75378	AL KHABURAH	Low	1

503	56.73353	23.88939	AL KHABURAH	Low	1
504	56.71557	23.91817	AL KHABURAH	Low	1
505	56.75577	23.91934	AL KHABURAH	Low	1
506	57.33453	22.65053	BAHLA	Mid	1
507	57.78381	23.00139	IZKI	High	1
508	57.68222	22.92572	NIZWA	Mid	1
509	57.53269	23.02179	NIZWA	No	0
510	57.99274	23.30581	SAMIL	No	0
511	57.83416	23.11744	SAMIL	No	0
512	57.83412	23.11699	SAMIL	No	0
513	57.45875	23.05142	NIZWA	Low	1
514	57.54773	22.96355	NIZWA	Low	1
515	58.10255	23.25173	DID DID	No	0
516	58.12209	23.40451	DID DID	Low	1
517	58.11314	23.34406	DID DID	Low	1
518	58.13411	23.43486	DID DID	Low	1
519	58.09999	23.45786	DID DID	Low	1
520	58.08960	23.41140	DID DID	Low	1
521	58.21758	23.45042	DID DID	Low	1
522	58.02759	23.30436	SAMIL	Low	1
523	58.10207	23.46839	DID DID	Low	1
524	58.14225	23.21667	DID DID	Mid	1
525	58.07741	23.21658	DID DID	Low	1
526	57.85086	23.15064	SAMIL	Low	1
527	57.98483	23.01821	SAMIL	Mid	1
528	58.00032	23.01760	SAMIL	Low	1
529	58.01737	23.00144	SAMIL	Low	1
530	58.08362	23.08417	SAMIL	Low	1
531	58.10314	23.38893	DID DID	Mid	1
532	57.31717	22.61778	BAHLA	No	0
533	57.16085	23.10171	BAHLA	Mid	1
534	57.13132	23.08310	BAHLA	Low	1
535	58.23351	23.24162	DID DID	Low	1
536	58.10662	23.36513	DID DID	Low	1
537	57.25072	22.91722	BAHLA	No	0
538	57.29732	23.08512	AL HAMRA	Low	1
539	57.29298	23.10125	AL HAMRA	Low	1
540	58.53422	22.98342	DAMA WAATTAIYIN	Mid	1
541	58.00031	22.55047	AL MUDAYBI	Low	1
542	58.88208	22.90360	DAMA WAATTAIYIN	Mid	1
543	58.82305	22.94863	DAMA WAATTAIYIN	Low	1
544	58.05294	22.86096	AL MUDAYBI	Mid	1
545	59.38350	22.53735	SUR	Low	1
546	59.25177	22.64524	SUR	Mid	1
547	58.52992	22.72722	IBRA	High	1
548	58.79777	22.97750	DAMA WAATTAIYIN	Low	1
549	58.02521	22.68792	AL MUDAYBI	Low	1
550	58.04168	22.61454	AL MUDAYBI	Mid	1
551	58.01812	22.57750	AL MUDAYBI	Low	1
552	58.01781	22.55142	AL MUDAYBI	High	1
553	56.50605	23.23055	IBRI	High	1
554	56.53596	23.60456	YNQAL	Low	1
555	56.53483	23.28367	IBRI	High	1
556	56.95766	23.07200	IBRI	Low	0
557	56.98263	23.05935	IBRI	No	0
558	56.93560	23.11521	IBRI	No	0
559	56.84430	23.07639	IBRI	Low	1
560	56.95953	23.20904	IBRI	No	0
561	56.56963	24.05293	SOHR	Mid	1
562	57.54608	23.29972	AL RUSTAQ	High	1
563	58.50357	22.75843	IBRA	High	1
564	58.41814	22.90022	IBRA	High	1

565	58.41806	22.88442	IBRA	High	1
566	58.55828	23.43568	AL AMRAT	High	1
567	58.82996	23.15936	QURAYYAT	High	1
568	58.98838	23.17523	QURAYYAT	High	1
569	58.75329	23.27584	QURAYYAT	High	1
570	56.25069	24.01814	AL BURAYMI	High	1
571	56.18074	24.71918	MAHADAH	High	1
572	56.25519	26.18992	KHASAB	High	1
573	57.48896	23.52144	AL RUSTAQ	High	1
574	57.44401	23.57437	AL RUSTAQ	High	1
575	57.33863	23.54144	AL RUSTAQ	High	1
576	57.22600	23.51133	AL RUSTAQ	High	1
577	57.18275	23.48419	AL RUSTAQ	High	1
578	57.32324	23.43291	AL RUSTAQ	High	1
579	57.44251	23.56513	AL RUSTAQ	High	1
580	57.18986	23.50627	AL RUSTAQ	High	1
581	57.22927	23.55525	AL RUSTAQ	High	1
582	56.30136	24.38422	MAHADAH	No	0
583	56.26574	24.20176	AL BURAYMI	No	0
584	55.96794	24.40047	MAHADAH	Low	1
585	56.03450	24.36808	MAHADAH	No	0
586	56.03444	24.38358	MAHADAH	No	0
587	56.06090	24.43788	MAHADAH	No	0
588	56.06736	24.48383	MAHADAH	Low	1
589	56.07401	24.62861	MAHADAH	No	0
590	56.16717	24.68442	MAHADAH	No	0
591	56.18209	24.72126	MAHADAH	No	0
592	56.25376	24.09510	AL BURAYMI	Mid	1
593	56.11806	24.30139	MAHADAH	Low	1
594	56.11794	24.31694	MAHADAH	High	1
595	58.58615	23.46476	QURAYYAT	Mid	1
596	58.61420	23.49127	QURAYYAT	Low	1
597	58.51764	23.40059	QURAYYAT	Low	1
598	58.55166	23.23452	QURAYYAT	Low	1
599	58.94110	23.04507	QURAYYAT	Mid	1
600	58.47894	23.46301	QURAYYAT	Low	1
601	58.83272	23.14944	QURAYYAT	Mid	1
602	58.86509	23.09078	QURAYYAT	Mid	1
603	58.85741	23.08895	QURAYYAT	Mid	1
604	58.58478	23.26683	QURAYYAT	Mid	1
605	58.74044	23.32189	QURAYYAT	Mid	1
606	58.78389	23.25089	QURAYYAT	Mid	1
607	57.52211	22.54404	DABA	Low	1
608	57.12735	23.06406	DABA	Low	1
609	57.38795	23.19047	DABA	No	0
610	56.70161	23.91744	AL KHABURAH	Low	1
611	57.41763	23.39177	AL RUSTAQ	Low	1
612	56.98372	23.80122	AL KHABURAH	No	0
613	56.92316	23.67148	AL KHABURAH	No	0
614	57.54904	23.30233	AL RUSTAQ	Mid	1
615	57.52900	23.31026	AL RUSTAQ	Low	1
616	57.64961	23.18082	AL RUSTAQ	Mid	1
617	57.31124	23.46058	AL RUSTAQ	Low	1
618	57.31110	23.46366	AL RUSTAQ	Low	1
619	57.33815	23.54770	AL RUSTAQ	Low	1
620	57.33689	23.55517	AL RUSTAQ	Low	1
621	57.32960	23.52888	AL RUSTAQ	Low	1
622	56.28484	24.45020	LIWA	Low	1
623	57.21259	23.57584	AL RUSTAQ	Low	1
624	57.03696	23.45799	AL RUSTAQ	Low	1
625	56.36767	24.35103	LIWA	Low	1
626	57.11234	23.49013	AL RUSTAQ	Low	1

627	56.35219	24.23003	SUR	Low	1
628	56.31706	24.19065	SUR	No	0
629	57.31855	23.38738	AL RUSTAQ	Low	1
630	57.30551	23.36961	AL RUSTAQ	Low	1
631	57.31967	23.31256	AL RUSTAQ	Mid	1
632	57.32253	23.22372	AL RUSTAQ	Low	1
633	57.33362	23.22467	AL RUSTAQ	Low	1
634	57.21524	23.54483	AL RUSTAQ	Low	1
635	57.19045	23.50824	AL RUSTAQ	Low	1
636	56.86775	23.78442	SAHAM	Low	1
637	56.51147	24.00424	SUR	Mid	1
638	58.14018	23.21489	BID BID	Mid	1
639	58.16172	23.40195	BID BID	High	1
640	57.12632	23.03852	BAHLA	Low	1
641	57.13823	22.97857	BAHLA	No	0
642	58.14159	23.40749	BID BID	High	1
643	58.09419	23.41095	BID BID	Low	1
644	58.09555	23.42365	BID BID	Low	1
645	58.10348	23.45752	BID BID	Low	1
646	58.11706	23.43232	BID BID	Low	1
647	58.10154	23.46182	BID BID	Low	1
648	57.32288	23.08504	AL HAMRA	No	0
649	58.13365	23.42076	BID BID	Low	1
650	57.26764	23.06700	AL HAMRA	Low	1
651	57.32826	23.08423	AL HAMRA	Low	1
652	58.04636	23.30681	SAMIL	Mid	1
653	58.03906	23.28214	SAMIL	Low	1
654	58.03262	23.30276	SAMIL	Low	1
655	57.83379	23.10094	SAMIL	Low	1
656	57.12096	23.04205	BAHLA	Low	1
657	57.85022	23.15156	SAMIL	Mid	1
658	57.86690	23.18352	SAMIL	Low	1
659	57.86786	23.21703	SAMIL	Mid	1
660	57.90111	23.16800	SAMIL	Low	1
661	57.83385	23.49694	IZKI	Low	1
662	57.98855	23.30590	SAMIL	Mid	1
663	57.86721	23.16804	SAMIL	Mid	1
664	58.01822	23.35083	SAMIL	Low	1
665	58.10900	23.36977	BID BID	High	1
666	58.08336	23.20108	BID BID	Low	1
667	58.06741	23.22768	BID BID	Low	1
668	58.07876	23.23538	BID BID	Low	1
669	58.23993	23.24647	BID BID	High	1
670	57.24638	23.09884	AL HAMRA	Low	1
671	58.11761	23.33378	BID BID	High	1
672	57.12525	23.05890	BAHLA	Low	1
673	57.12477	23.05660	BAHLA	Low	1
674	58.07956	23.31680	SAMIL	Low	1
675	57.29982	23.10943	AL HAMRA	High	1
676	57.30064	23.08475	AL HAMRA	High	1
677	58.01365	23.34770	SAMIL	Mid	1
678	57.46541	23.05238	NIZWA	Low	1
679	57.53509	23.02059	NIZWA	Low	1
680	57.67309	22.98806	NIZWA	Low	1
681	57.14275	23.09146	BAHLA	Mid	1
682	57.78392	22.98358	IZKI	Low	1
683	57.55755	22.96801	NIZWA	Mid	1
684	57.51806	22.36753	ADAM	Low	1
685	57.15027	22.98374	BAHLA	Mid	1
686	57.13964	22.99851	BAHLA	Low	1
687	58.34633	23.09388	AL MUDAYBI	No	0
688	57.99738	22.72076	AL MUDAYBI	Low	1

689	58.00017	22.78392	AL MUDAYBI	Low	1
690	57.99864	22.61357	AL MUDAYBI	Low	1
691	58.01761	22.50131	AL MUDAYBI	Low	1
692	58.11700	22.50011	AL MUDAYBI	Low	1
693	58.06937	22.30063	AL MUDAYBI	Low	1
694	58.12477	22.52247	AL MUDAYBI	Low	1
695	58.11703	22.53378	AL MUDAYBI	Low	1
696	58.12829	22.58308	AL MUDAYBI	Low	1
697	58.13579	22.61078	AL MUDAYBI	Low	1
698	58.12204	22.56432	AL MUDAYBI	Low	1
699	58.17757	22.54503	AL MUDAYBI	Low	1
700	58.19386	22.57139	AL MUDAYBI	No	0
701	58.18361	22.60033	AL MUDAYBI	Low	1
702	58.21683	22.38344	AL MUDAYBI	Low	1
703	58.01514	22.37300	AL MUDAYBI	Low	1
704	58.21692	22.37634	AL MUDAYBI	Low	1
705	57.99344	22.67319	AL MUDAYBI	Low	1
706	58.00162	22.61325	AL MUDAYBI	Low	1
707	58.13347	22.80014	AL MUDAYBI	Low	1
708	58.17651	22.70616	AL MUDAYBI	Low	1
709	56.96684	23.07024	IBRI	Low	1
710	56.87783	23.11580	IBRI	No	0
711	57.07340	23.10807	IBRI	Low	1
712	57.08746	23.12641	IBRI	Low	1
713	56.99660	23.05789	IBRI	Low	1
714	56.82978	23.49272	IBRI	No	0
715	56.40493	23.86139	YANQL	No	0
716	56.39995	23.93132	YANQL	No	0
717	56.34155	23.94477	YANQL	No	0
718	57.00209	23.40133	IBRI	Low	1
719	56.90982	23.16985	IBRI	High	1
720	56.41027	23.83645	YANQL	No	0
721	57.03722	23.22377	IBRI	Low	1
722	57.02558	23.24793	IBRI	Low	1
723	56.83961	23.07538	IBRI	Low	1
724	56.89874	23.28092	IBRI	Low	1
725	56.92994	23.28632	IBRI	Mid	1
726	56.41828	23.88424	YANQL	No	0
727	56.87896	23.37989	IBRI	Low	1
728	56.91933	23.38319	IBRI	Low	1
729	56.92031	23.38516	IBRI	Low	1
730	56.96644	23.37572	IBRI	Low	1
731	57.00106	23.48436	IBRI	Low	1
732	56.89121	23.50209	IBRI	Low	1
733	56.79309	23.46499	IBRI	Mid	1
734	56.75464	23.43015	IBRI	Low	1
735	56.18469	23.93372	DANK	Mid	1
736	58.51719	23.23475	AL AMRAT	High	1
737	56.23897	26.19016	KHASAB	High	1
738	58.15498	23.37774	BID BID	Low	1
739	58.07340	23.22946	BID BID	No	0
740	58.13359	23.42778	BID BID	Mid	1
741	58.04511	23.49266	BID BID	Low	1
742	58.09764	23.44192	BID BID	Low	1
743	58.11998	23.45175	BID BID	Low	1
744	58.12452	23.41009	BID BID	Low	1
745	56.41585	23.88452	YANQAL	No	0
746	56.40909	23.82169	YANQAL	No	0
747	57.26491	23.07381	AL HAMRA	No	0
748	57.31193	23.10270	AL HAMRA	High	1
749	56.23481	25.63353	DABA	No	0
750	56.39195	23.92831	IBRI	Low	1

751	56.33760	23.94306	YANQAL	No	0
752	56.36803	23.85161	YANQAL	No	0
753	56.92596	23.13934	IBRI	Low	1
754	56.95575	23.07176	IBRI	High	1
755	56.95282	23.05111	IBRI	Low	1
756	58.13206	23.41789	BID BID	Low	1
757	58.14193	23.40561	BID BID	Low	1
758	56.52949	23.56040	YANQAL	No	0
759	57.12254	23.04534	BAHLA	No	0
760	57.11968	23.04559	BAHLA	low	1
761	57.15142	22.98397	BAHLA	Mid	1
762	57.33378	22.93478	BAHLA	No	0
763	57.53577	23.02145	NIZWA	Low	1
764	57.30139	22.91756	BAHLA	No	0
765	57.28453	22.93392	BAHLA	No	0
766	57.55555	22.96860	NIZWA	Mid	1
767	56.27835	25.26917	MADHA	No	0
768	56.75863	23.14015	IBRI	No	0
769	56.85527	23.08567	IBRI	Low	1
770	58.23441	23.23995	BID BID	Low	1
771	57.03450	23.25131	IBRI	Low	1
772	57.03889	23.23043	IBRI	Low	1
773	57.27924	23.08339	AL HAMRA	Low	1
774	57.75075	22.81731	IZKI	Low	1
775	57.74161	23.37667	IZKI	Low	1
776	56.30993	25.26577	MADHA	No	0
777	57.67374	22.99115	NIZWA	Low	1
778	57.76683	22.90108	IZKI	Low	1
780	56.25139	25.63469	DABA	No	0
781	56.26397	25.64310	DABA	No	0
782	57.47784	23.04934	NIZWA	Low	1
783	57.13369	23.05125	BAHLA	No	0
784	56.28205	25.26863	MADHA	No	0
785	56.29534	25.26440	MADHA	No	0
786	56.39691	23.81766	YANQAL	High	1
787	56.41280	23.87748	YANQAL	No	0
788	56.49095	23.98597	YANQAL	Low	1
789	56.33870	23.94415	YANQAL	Mid	1
790	57.45879	23.04191	NIZWA	Low	1
791	55.79237	24.23822	AL BURAYMI	Mid	1
792	56.25108	25.63403	DABA	No	0
793	56.24147	26.18938	KHASAB	High	1
794	56.25043	25.66646	DABA	No	0
795	58.13779	23.40008	BID BID	No	0
796	57.33367	22.93461	BAHLA	Low	1
797	58.23025	23.26243	BID BID	No	0
798	58.10532	23.48820	BID BID	Low	1
799	57.28381	23.08392	AL HAMRA	Low	1
800	58.11155	23.45256	BID BID	No	0
801	58.11736	23.45061	BID BID	No	0
802	57.97556	23.29614	SAMIL	No	0
803	58.14727	23.38162	BID BID	Low	1
804	57.53566	23.02076	NIZWA	Low	1
805	57.13863	22.98461	BAHLA	Low	1
806	57.12929	23.07154	BAHLA	Low	1
807	58.07768	23.22504	BID BID	No	0
808	58.13891	23.40298	BID BID	No	0
809	56.06808	24.36711	MAHADAH	Low	1
810	57.29630	23.08405	AL HAMRA	Mid	1
811	57.30747	23.10482	AL HAMRA	High	1
812	57.76733	22.91783	IZKI	Low	1
813	56.30100	24.36828	MAHADAH	Low	1

814	56.31658	25.26660	MADHA	No	0
815	57.54886	22.97042	NIZWA	Mid	1
816	57.53406	22.93403	NIZWA	High	1
817	57.53823	23.02019	NIZWA	Low	1
818	57.61560	22.73650	MANAH	Low	1
819	57.76803	22.76803	IZKI	Low	1
820	56.26367	25.65725	DABA	No	0
821	56.25552	25.61469	DABA	No	0
822	57.30044	22.95139	BAHLA	Mid	1
823	57.30133	23.00017	BAHLA	Mid	1
824	56.15500	24.55137	MAHADAH	Low	1
825	57.83381	23.10147	SAMIL	Low	1
826	57.82942	23.39014	IZKI	Low	1
827	58.36229	23.12222	AL MDAYBI	No	0
828	58.31136	23.16167	BID BID	Low	1
829	57.53155	23.30447	AL RUSTAQ	Low	1
830	57.45483	23.49341	AL RUSTAQ	Low	1
831	56.40910	23.86895	YANQAL	Mid	1
832	57.31413	23.39036	AL RUSTAQ	No	0
833	57.30734	23.36798	AL RUSTAQ	Low	1
834	57.33719	23.21073	AL RUSTAQ	No	0
835	57.31668	23.21908	AL RUSTAQ	No	0
836	57.32780	23.28415	AL RUSTAQ	Low	1
837	56.96018	23.07053	IBRI	High	1
838	56.94782	23.05325	IBRI	Low	1
839	57.08104	23.14729	IBRI	Mid	1
840	57.06797	23.10119	IBRI	Mid	1
841	56.25006	25.61686	DABA	No	0
842	57.76728	22.88461	IZKI	No	0
843	57.75100	22.81817	IZKI	No	0
844	57.75033	22.81700	IZKI	No	0
845	57.28208	23.08250	AL HAMRA	No	0
846	58.13994	23.39790	BID BID	No	0
847	58.15881	23.37671	BID BID	No	0
848	57.62625	23.18610	AL AWABI	High	1
849	57.55471	23.21845	AL AWABI	No	0
850	57.53024	23.30894	AL RUSTAQ	No	0
851	56.41938	23.88444	YANQAL	High	1
852	56.38962	23.86313	YANQAL	No	0
853	58.06831	23.31703	SAMIL	High	1
854	58.03634	23.28004	SAMIL	No	0
855	57.12727	23.43369	AL RUSTAQ	No	0
856	56.35036	23.94119	YANQAL	No	0
857	57.28446	23.11966	AL HAMRA	High	1
858	57.55395	22.97354	NIZWA	Mid	1
859	58.01808	23.35050	SAMIL	Mid	1
860	58.30604	23.10755	BID BID	Low	1
861	58.30912	23.11211	AL MDAYBI	No	0
862	57.52885	22.88179	NIZWA	Mid	1
863	58.98217	23.18929	QURAYYAT	High	1
864	58.05072	23.36050	SAMIL	Mid	1
865	57.45174	23.03829	NIZWA	Mid	1
866	57.53748	23.02042	NIZWA	Mid	1
867	58.01725	22.98497	SAMIL	High	1
868	56.81719	23.08344	IBRI	Low	1
869	58.10056	23.08419	SAMIL	Mid	1
870	56.90164	23.16728	IBRI	Low	1
871	57.99949	23.30823	SAMIL	Mid	1
872	55.97160	24.21216	AL BURAYMI	No	0
873	56.39288	23.94016	YANQAL	Low	1
874	56.34955	23.93530	YANQAL	Mid	1
875	57.81114	23.42499	IZKI	Low	1

876	57.04537	23.23519	IBRI	Low	1
877	56.36761	23.85136	YANQAL	Mid	1
878	58.13397	23.21753	BID BID	Low	1
879	56.90039	23.13464	IBRI	Low	1
880	56.32339	25.28169	MADHA	No	0
881	56.32140	25.27984	MADHA	No	0
882	56.30745	25.26439	MADHA	No	0
883	58.11973	23.44781	BID BID	Low	1
884	58.12529	23.44541	BID BID	Low	1
885	56.25011	25.61689	DABA	No	0
886	56.23756	24.20655	AL BURAYMI	Mid	1
887	56.94413	23.04683	IBRI	Low	1
888	56.21689	24.03439	AL BURAYMI	Mid	1
889	56.94324	23.06515	IBRI	High	1
890	58.08637	23.48013	BID BID	Low	1
891	56.21940	26.19610	KHASAB	High	1
892	57.08689	23.14350	IBRI	Mid	1
893	57.08797	23.14558	IBRI	Mid	1
894	56.10094	24.55064	AL BURAYMI	Low	1
895	56.16747	24.70144	MAHADAH	No	0
896	57.31341	23.30054	AL RUSTAQ	Low	1
897	57.33003	23.22895	AL RUSTAQ	Low	1
898	57.33344	23.21781	AL RUSTAQ	Low	1
899	57.47745	23.55288	AL RUSTAQ	Low	1
900	57.06256	23.45291	AL RUSTAQ	Mid	1
901	57.83319	23.38461	NAKHAL	Mid	1
902	57.81598	23.42685	WADI AL MAAWIL	High	1
903	58.13694	23.67897	AlMaabailah	Low	1
904	58.12154	23.56999	Old AlKhoudh	Low	1
905	58.18989	23.54225	AlJafnain	Low	1
906	58.39925	23.53911	AlHammam	Low	1
907	58.34746	23.48700	Old Bosher	Low	1
908	58.50102	23.52885	Madenat AlNahdhah	Low	1
909	58.51533	23.40708	AlHajir	Low	1
910	58.59108	23.26019	Bii	High	1
911	58.66122	23.50692	Yiti	Low	1
912	58.67122	23.46853	AlHilo/Muscat	Low	1
913	58.76942	23.43614	AlSeefah/Muscat	Low	1
914	58.91337	23.27050	Daghmar/Qurayat	No	0
915	58.99178	23.03425	/adi AlArbiyeen/Quray	Low	1
916	59.05028	23.07550	Dhabab/Qurayat	Low	1
917	59.24750	22.80764	Tiwi/Sur	Low	1
918	59.48400	22.54858	Bilad Sur	Low	1
919	58.46286	23.12272	yadah/Dama wa Attay	Low	1
920	58.81017	22.96125	Bani Said/Dama wa A	Low	1
921	58.10386	23.46967	Fanja	Low	1
922	58.12932	23.41565	faa/Interior of Bidbid (	Low	1
923	58.10689	23.37264	Suroor/Samail	Low	1
924	57.84561	23.14006	/adi Bani Rawaha/AlE	High	1
925	58.08106	23.23356	AlFatih	Low	1
926	59.31360	22.02429	an Bani Bo Ali/Hadra B	Low	1
927	59.46614	22.54058	n Bani Bo Hassan/Rav	Low	1
928	59.20647	22.21553	AlDreez/AlKamil	Low	1
929	59.12470	22.50761	Sabit	Low	1
930	59.08558	22.59669	Wadi Bani Khalid/Muqa	Low	1
931	58.82853	22.59404	/adi Bani Khalid/AlHay	Low	1
932	58.21719	23.02169	AlJarda/AlMudhaibi	Low	1
933	58.22347	22.88525	Samad/AlRawdah	No	0
934	58.05928	22.37053	Barzaman	Low	1
935	58.39250	22.84189	Ibra/AlQaa	Low	1
936	58.68451	22.57866	AlQabil	Low	1
937	58.78045	22.43644	Bidiyeh	Low	1

938	57.73595	23.13976	Wakan/Nakhal	No	0
939	57.91903	23.51061	Wadi AlMaawal/AlAjal	High	1
940	57.80946	23.68274	AlHafri/Barka Coast	Low	1
941	57.00875	23.49336	AlHayal/Ibri	Low	1
942	56.61753	23.31200	AlDiriez	Low	1
943	56.28156	23.40242	Ibri/AlMazim	Low	1
944	57.30645	23.48626	AlMadinah/Rustaq	Low	1
945	57.56389	23.56572	Gama	Low	1
946	57.09944	23.84183	Khaborah/Bani Rabiaa	Low	1
947	56.36104	23.83638	Wadi AlHareem	Low	1
948	56.48832	23.63230	Yanqul/AlBuweedrah	Low	1
949	56.88522	24.15581	AlHaweel	Low	1
950	56.25627	25.60620	Daba/Oman AlArabiya	Low	1
951	56.10739	26.07364	Tibaat	Low	1
952	56.13964	26.12186	Bakha/Fadhgha	Low	1
953	56.17503	26.16395	AlJadii	Low	1
954	56.24144	26.19319	Khasab/AlHajer	Mid	1
955	56.22054	26.19287	Khasab/Kada	Low	1
956	57.66125	23.73714	Abu Abali	Low	1
957	57.55005	22.71776	Manah/Aizz	Low	1
958	57.52144	22.38153	Adam/Gamaa Albusaid	Low	1
959	57.91725	21.80692	Adam/Brimah	Low	1
960	57.46031	23.05228	Tanuf	Low	1
961	57.67472	23.04598	Masserit AlRawajh	Low	1
962	57.75317	22.82019	Qalaat AlAwamir	Low	1
964	56.70775	24.39872	Sohar/AlKhashabah	Low	1
965	56.78261	24.24831	Sohar/AlAweenat	Low	1
966	56.56514	24.55086	Liwa/AlZahiyah	Low	1
967	55.94840	24.73451	Mahadhah AlRawdhah	Low	1
968	56.10608	24.54719	Mahadhah AlJuweef	Low	1
969	55.96711	23.61858	AlNasinah AlRihani	Low	1
970	57.31007	23.13810	Misfaat' AlAbrieen	Mid	1
971	56.54022	23.51683	Dhank/Fida	Low	1
972	56.26650	23.56227	Dhank/Wadi Dhank	Low	1
