# Supplementary material for: Impact of environmental variables on Dubas bug infestation rate: A case study from the Sultanate of Oman
Source: PLoS One. 2017 May 30;12(5):e0178109. doi: 10.1371/journal.pone.0178109 (PMC5448759; doi:10.1371/journal.pone.0178109)
Supplement: S2 File — (PDF) [file pone.0178109.s002.pdf]

| ID | DB DENSITY | LMiIndex | LMiZScore | LMiPValue | COType |
|---|---|---|---|---|---|
| 1 | 30 | -2.355442 | -1.665384 | 0.095836 | |
| 2 | 30 | -0.061631 | -0.018950 | 0.984881 | |
| 3 | 30 | 1.584098 | 0.603482 | 0.546188 | |
| 4 | 0 | 9.218532 | 1.872637 | 0.061119 | |
| 5 | 0 | 8.708588 | 1.429553 | 0.152845 | |
| 6 | 30 | -1.995289 | -0.498700 | 0.617990 | |
| 7 | 30 | 2.777226 | 1.393190 | 0.163562 | |
| 8 | 0 | 10.048895 | 3.196681 | 0.001390 | LL |
| 9 | 0 | 8.300905 | 1.497731 | 0.134203 | |
| 10 | 0 | 10.136158 | 2.415356 | 0.015720 | LL |
| 11 | 10 | 7.900936 | 2.648360 | 0.008088 | LL |
| 12 | 0 | 8.213642 | 1.702379 | 0.088684 | |
| 14 | 30 | 4.875554 | 2.187798 | 0.028684 | HH |
| 15 | 10 | -0.138180 | -0.137185 | 0.890884 | |
| 16 | 30 | 6.037867 | 2.474497 | 0.013342 | HH |
| 17 | 20 | -0.696898 | -0.230220 | 0.817921 | |
| 18 | 0 | 2.417461 | 0.812476 | 0.416518 | |
| 19 | 20 | -1.802335 | -0.436611 | 0.662394 | |
| 20 | 30 | -0.966829 | -0.482226 | 0.629645 | |
| 21 | 0 | 4.704028 | 1.928409 | 0.053804 | |
| 22 | 0 | 1.004889 | 1.006181 | 0.314328 | |
| 23 | 0 | 1.004889 | 1.006181 | 0.314328 | |
| 24 | 30 | -8.999985 | -2.418371 | 0.015590 | HL |
| 25 | 0 | 11.461467 | 2.730589 | 0.006322 | LL |
| 26 | 20 | 0.314420 | 0.183551 | 0.854366 | |
| 27 | 0 | 14.112084 | 3.361054 | 0.000776 | LL |
| 28 | 0 | -11.432833 | -2.970225 | 0.002976 | LH |
| 29 | 30 | 3.877909 | 1.086269 | 0.277360 | |
| 30 | 30 | 10.790160 | 2.110929 | 0.034778 | HH |
| 32 | 20 | -0.232299 | -0.132505 | 0.894585 | |
| 33 | 30 | 9.720292 | 2.619995 | 0.008793 | HH |
| 35 | 30 | 10.625491 | 2.531747 | 0.011350 | HH |
| 36 | 30 | 8.527163 | 2.090228 | 0.036597 | HH |
| 37 | 0 | -13.442612 | -3.748815 | 0.000178 | LH |
| 38 | 30 | 9.010578 | 2.348115 | 0.018869 | HH |
| 39 | 0 | 8.723586 | 2.775515 | 0.005511 | LL |
| 40 | 30 | 1.584098 | 0.603482 | 0.546188 | |
| 41 | 30 | 1.584098 | 0.603482 | 0.546188 | |
| 42 | 0 | -3.888663 | -1.377270 | 0.168429 | |
| 43 | 20 | -1.934515 | -0.516774 | 0.605314 | |
| 44 | 30 | -2.705003 | -0.657425 | 0.510908 | |
| 45 | 30 | -6.253574 | -1.343396 | 0.179144 | |
| 46 | 30 | 1.584098 | 0.603482 | 0.546188 | |
| 47 | 30 | 2.777226 | 1.393190 | 0.163562 | |
| 48 | 30 | 4.104209 | 1.108485 | 0.267652 | |
| 49 | 30 | 4.104209 | 1.108485 | 0.267652 | |
| 50 | 30 | 1.296167 | 0.352730 | 0.724291 | |
| 52 | 0 | 11.738254 | 3.280512 | 0.001036 | LL |
| 53 | 30 | 11.592320 | 3.123832 | 0.001785 | HH |
| 54 | 30 | 9.267693 | 2.694934 | 0.007040 | HH |
| 55 | 30 | 0.164669 | 0.058235 | 0.953562 | |
| 56 | 30 | 0.164669 | 0.058235 | 0.953562 | |
| 57 | 10 | 0.433355 | 0.434498 | 0.663927 | |
| 58 | 0 | 0.407682 | 0.157342 | 0.874975 | |
| 59 | 30 | 3.939540 | 1.768226 | 0.077023 | |
| 60 | 30 | 3.939540 | 1.768226 | 0.077023 | |
| 61 | 10 | 0.433355 | 0.434498 | 0.663927 | |
| 62 | 30 | 1.614913 | 0.935362 | 0.349602 | |
| 63 | 30 | 6.943066 | 2.209689 | 0.027127 | HH |
| 64 | 30 | -2.159958 | -0.816402 | 0.414270 | |
| 65 | 20 | -0.785018 | -0.294974 | 0.768014 | |

| | | | | | |
|---|---|---|---|---|---|
| 66 | 0 | -1.645728 | -1.645126 | 0.099944 | |
| 67 | 30 | -0.030815 | -0.013372 | 0.989331 | |
| 68 | 20 | 0.126180 | 0.050586 | 0.959655 | |
| 69 | 20 | 0.082120 | 0.036151 | 0.971162 | |
| 70 | 20 | 0.126180 | 0.050586 | 0.959655 | |
| 71 | 20 | -0.006000 | -0.000942 | 0.999249 | |
| 72 | 30 | 4.649254 | 2.330898 | 0.019759 | HH |
| 73 | 30 | 4.649254 | 2.330898 | 0.019759 | HH |
| 74 | 30 | 4.649254 | 2.330898 | 0.019759 | HH |
| 76 | 30 | 2.777226 | 1.393190 | 0.163562 | |
| 77 | 30 | 2.777226 | 1.393190 | 0.163562 | |
| 78 | 30 | 0.678899 | 0.394255 | 0.693393 | |
| 79 | 30 | -1.676543 | -0.749210 | 0.453730 | |
| 80 | 30 | 2.777226 | 1.393190 | 0.163562 | |
| 81 | 0 | 2.373830 | 1.066389 | 0.286248 | |
| 82 | 30 | 4.587624 | 1.335838 | 0.181602 | |
| 83 | 10 | 0.747346 | 0.286155 | 0.774759 | |
| 84 | 30 | 0.874383 | 0.313267 | 0.754078 | |
| 85 | 30 | -0.483415 | -0.340633 | 0.733380 | |
| 86 | 30 | 0.390969 | 0.127518 | 0.898531 | |
| 87 | 30 | -3.548571 | -1.588356 | 0.112206 | |
| 88 | 30 | -3.548571 | -1.588356 | 0.112206 | |
| 89 | 30 | 1.388613 | 0.984117 | 0.325058 | |
| 90 | 30 | 1.584098 | 0.603482 | 0.546188 | |
| 91 | 10 | 1.670504 | 0.373215 | 0.708988 | |
| 92 | 0 | 3.335088 | 2.361551 | 0.018199 | LL |
| 93 | 30 | -4.515400 | -1.508666 | 0.131384 | |
| 94 | 10 | 1.871599 | 1.083751 | 0.278475 | |
| 95 | 20 | -1.564036 | -0.493758 | 0.621477 | |
| 96 | 10 | -0.376907 | -0.110827 | 0.911753 | |
| 97 | 30 | -8.279678 | -1.298973 | 0.193953 | |
| 98 | 10 | -0.238727 | -0.072592 | 0.942131 | |
| 99 | 10 | 0.866710 | 0.614790 | 0.538694 | |
| 100 | 30 | 5.040223 | 1.360403 | 0.173702 | |
| 101 | 20 | -2.355054 | -0.514659 | 0.606792 | |
| 102 | 10 | 4.785720 | 1.249021 | 0.211658 | |
| 103 | 10 | -4.220653 | -1.132064 | 0.257608 | |
| 104 | 10 | 4.785720 | 1.249021 | 0.211658 | |
| 105 | 30 | 2.715596 | 0.792200 | 0.428244 | |
| 106 | 10 | 5.080895 | 1.247190 | 0.212328 | |
| 107 | 30 | -6.675358 | -1.678173 | 0.093313 | |
| 108 | 30 | 1.584098 | 0.603482 | 0.546188 | |
| 109 | 10 | 2.757125 | 0.926197 | 0.354344 | |
| 110 | 10 | 14.363628 | 3.624078 | 0.000290 | LL |
| 111 | 0 | 34.952974 | 9.097011 | 0.000000 | LL |
| 112 | 0 | 34.952974 | 9.097011 | 0.000000 | LL |
| 113 | 0 | 11.650991 | 5.224920 | 0.000000 | LL |
| 114 | 30 | 5.297338 | 1.609353 | 0.107539 | |
| 115 | 30 | -0.709714 | -0.708869 | 0.478406 | |
| 116 | 30 | 2.550927 | 1.476468 | 0.139818 | |
| 117 | 30 | -0.709714 | -0.708869 | 0.478406 | |
| 118 | 30 | 2.067512 | 0.929080 | 0.352847 | |
| 119 | 30 | 2.067512 | 0.929080 | 0.352847 | |
| 120 | 30 | 1.614913 | 0.935362 | 0.349602 | |
| 121 | 30 | 2.777226 | 1.393190 | 0.163562 | |
| 122 | 30 | 4.392139 | 1.668396 | 0.095237 | |
| 123 | 10 | 3.479187 | 0.561795 | 0.574256 | |
| 124 | 30 | -9.215692 | -1.446593 | 0.148011 | |
| 125 | 30 | -7.796264 | -1.251916 | 0.210601 | |
| 126 | 0 | 28.646849 | 7.713879 | 0.000000 | LL |
| 127 | 0 | 26.316651 | 7.350123 | 0.000000 | LL |
| 128 | 20 | 0.176240 | 0.090343 | 0.928015 | |

| | | | | | |
|---|---|---|---|---|---|
| 129 | 0 | 6.670175 | 3.343188 | 0.000828 | LL |
| 130 | 0 | 6.670175 | 3.343188 | 0.000828 | LL |
| 131 | 20 | -0.188239 | -0.092227 | 0.926518 | |
| 133 | 30 | 0.874383 | 0.313267 | 0.754078 | |
| 134 | 30 | -3.774871 | -1.888788 | 0.058920 | |
| 135 | 20 | -0.646838 | -0.262293 | 0.793096 | |
| 136 | 30 | 1.069868 | 0.302395 | 0.762351 | |
| 137 | 30 | -9.514215 | -2.144486 | 0.031994 | HL |
| 138 | 30 | -1.861435 | -0.345219 | 0.729930 | |
| 139 | 30 | 11.139721 | 3.238572 | 0.001201 | HH |
| 140 | 30 | 11.139721 | 3.238572 | 0.001201 | HH |
| 141 | 30 | 11.139721 | 3.238572 | 0.001201 | HH |
| 142 | 30 | 12.302034 | 3.437893 | 0.000586 | HH |
| 143 | 30 | 11.139721 | 3.238572 | 0.001201 | HH |
| 144 | 30 | 5.297338 | 1.609353 | 0.107539 | |
| 145 | 20 | -0.188239 | -0.092227 | 0.926518 | |
| 146 | 30 | 3.908725 | 1.311756 | 0.189603 | |
| 147 | 10 | 7.775104 | 1.219434 | 0.222679 | |
| 148 | 10 | 0.295175 | 0.210340 | 0.833402 | |
| 149 | 10 | -0.119364 | -0.051197 | 0.959168 | |
| 150 | 30 | -9.905184 | -3.144466 | 0.001664 | HL |
| 151 | 0 | 10.048895 | 3.196681 | 0.001390 | LL |
| 152 | 0 | 9.044005 | 3.031062 | 0.002437 | LL |
| 153 | 30 | -1.645728 | -1.645126 | 0.099944 | |
| 154 | 10 | 3.605019 | 1.369891 | 0.170721 | |
| 155 | 10 | 2.028595 | 0.833058 | 0.404812 | |
| 156 | 10 | 7.210038 | 1.944386 | 0.051849 | |
| 157 | 10 | 6.205148 | 1.735926 | 0.082577 | |
| 158 | 10 | 5.909973 | 1.795077 | 0.072641 | |
| 159 | 10 | 5.909973 | 1.795077 | 0.072641 | |
| 160 | 10 | 2.185591 | 0.734845 | 0.462434 | |
| 161 | 0 | 39.613370 | 9.694649 | 0.000000 | LL |
| 162 | 30 | 2.324627 | 1.646493 | 0.099662 | |
| 163 | 30 | 3.003526 | 1.348653 | 0.177448 | |
| 164 | 20 | -0.232299 | -0.132505 | 0.894585 | |
| 165 | 20 | 0.452599 | 0.321742 | 0.747648 | |
| 166 | 20 | 0.132180 | 0.078199 | 0.937670 | |
| 167 | 20 | -0.100119 | -0.038460 | 0.969321 | |
| 168 | 20 | -0.320419 | -0.319473 | 0.749368 | |
| 169 | 30 | 1.614913 | 0.935362 | 0.349602 | |
| 170 | 30 | 2.324627 | 1.646493 | 0.099662 | |
| 171 | 30 | 2.293812 | 0.941641 | 0.346377 | |
| 172 | 30 | 2.777226 | 1.393190 | 0.163562 | |
| 173 | 30 | 3.939540 | 1.768226 | 0.077023 | |
| 174 | 30 | 3.939540 | 1.768226 | 0.077023 | |
| 175 | 30 | 10.460822 | 3.505417 | 0.000456 | HH |
| 176 | 30 | 3.003526 | 1.348653 | 0.177448 | |
| 177 | 30 | 10.460822 | 3.505417 | 0.000456 | HH |
| 178 | 30 | 8.136195 | 3.088280 | 0.002013 | HH |
| 179 | 30 | 9.298508 | 3.303223 | 0.000956 | HH |
| 180 | 30 | 8.136195 | 3.088280 | 0.002013 | HH |
| 181 | 30 | 9.298508 | 3.303223 | 0.000956 | HH |
| 182 | 30 | 5.811568 | 2.607371 | 0.009124 | HH |
| 184 | 30 | 2.550927 | 1.476468 | 0.139818 | |
| 185 | 30 | -0.966829 | -0.482226 | 0.629645 | |
| 186 | 30 | 1.388613 | 0.984117 | 0.325058 | |
| 188 | 30 | 3.486941 | 2.017574 | 0.043636 | HH |
| 189 | 30 | 5.811568 | 2.607371 | 0.009124 | HH |
| 190 | 30 | 6.973881 | 2.857711 | 0.004267 | HH |
| 191 | 30 | 1.357798 | 0.558427 | 0.576553 | |
| 192 | 30 | 3.229826 | 1.324855 | 0.185219 | |
| 193 | 30 | 1.162314 | 1.163646 | 0.244567 | |

| | | | | | |
|---|---|---|---|---|---|
| 194 | 30 | 2.036697 | 0.684995 | 0.493347 | |
| 195 | 30 | 7.200181 | 2.733309 | 0.006270 | HH |
| 196 | 30 | 6.973881 | 2.857711 | 0.004267 | HH |
| 197 | 30 | 5.811568 | 2.607371 | 0.009124 | HH |
| 198 | 30 | 1.162314 | 1.163646 | 0.244567 | |
| 199 | 30 | 1.162314 | 1.163646 | 0.244567 | |
| 200 | 30 | -1.645728 | -1.645126 | 0.099944 | |
| 201 | 30 | 9.627846 | 1.918795 | 0.055010 | |
| 202 | 30 | 7.303219 | 1.514249 | 0.129963 | |
| 203 | 30 | 13.598201 | 2.658867 | 0.007840 | HH |
| 204 | 30 | 1.162314 | 1.163646 | 0.244567 | |
| 205 | 30 | 1.357798 | 0.558427 | 0.576553 | |
| 206 | 30 | 0.195484 | 0.089935 | 0.928339 | |
| 207 | 30 | 7.848264 | 2.116158 | 0.034331 | HH |
| 208 | 30 | 5.523637 | 1.607657 | 0.107910 | |
| 209 | 30 | 6.685951 | 1.870144 | 0.061464 | |
| 210 | 30 | 7.848264 | 2.116158 | 0.034331 | HH |
| 211 | 30 | 6.685951 | 1.870144 | 0.061464 | |
| 212 | 30 | 9.298508 | 3.303223 | 0.000956 | HH |
| 213 | 30 | -2.869672 | -1.015602 | 0.309819 | |
| 214 | 30 | -2.931303 | -0.734595 | 0.462586 | |
| 215 | 30 | -3.867317 | -0.970489 | 0.331803 | |
| 216 | 30 | -0.287930 | -0.106460 | 0.915218 | |
| 217 | 30 | -0.483415 | -0.340633 | 0.733380 | |
| 218 | 30 | 3.713240 | 1.862044 | 0.062597 | |
| 219 | 30 | 4.875554 | 2.187798 | 0.028684 | HH |
| 220 | 30 | 2.550927 | 1.476468 | 0.139818 | |
| 221 | 30 | 0.452599 | 0.321742 | 0.747648 | |
| 222 | 30 | 0.452599 | 0.321742 | 0.747648 | |
| 223 | 30 | 2.550927 | 1.476468 | 0.139818 | |
| 224 | 30 | 2.550927 | 1.476468 | 0.139818 | |
| 225 | 30 | 2.777226 | 1.393190 | 0.163562 | |
| 226 | 30 | 2.324627 | 1.646493 | 0.099662 | |
| 227 | 30 | 2.715596 | 0.792200 | 0.428244 | |
| 228 | 30 | 5.040223 | 1.360403 | 0.173702 | |
| 229 | 30 | 3.003526 | 1.348653 | 0.177448 | |
| 230 | 30 | 3.003526 | 1.348653 | 0.177448 | |
| 231 | 30 | -0.257115 | -0.146851 | 0.883249 | |
| 234 | 30 | 1.810397 | 0.645484 | 0.518613 | |
| 235 | 30 | 3.682425 | 1.309919 | 0.190223 | |
| 236 | 30 | 3.682425 | 1.309919 | 0.190223 | |
| 237 | 30 | 2.324627 | 1.646493 | 0.099662 | |
| 239 | 30 | 2.324627 | 1.646493 | 0.099662 | |
| 240 | 30 | 3.486941 | 2.017574 | 0.043636 | HH |
| 241 | 30 | 3.486941 | 2.017574 | 0.043636 | HH |
| 244 | 30 | 2.324627 | 1.646493 | 0.099662 | |
| 245 | 30 | 1.388613 | 0.984117 | 0.325058 | |
| 246 | 30 | -0.030815 | -0.013372 | 0.989331 | |
| 247 | 0 | 13.514877 | 2.797829 | 0.005145 | LL |
| 248 | 30 | -11.087720 | -1.741832 | 0.081538 | |
| 249 | 30 | -12.250033 | -1.947594 | 0.051464 | |
| 250 | 30 | -1.419428 | -1.003009 | 0.315857 | |
| 251 | 30 | 2.777226 | 1.393190 | 0.163562 | |
| 252 | 30 | -16.642172 | -2.898551 | 0.003749 | HL |
| 253 | 0 | 13.194458 | 2.678050 | 0.007405 | LL |
| 255 | 0 | 7.208753 | 1.525854 | 0.127046 | |
| 256 | 10 | -1.557608 | -0.898662 | 0.368833 | |
| 257 | 20 | 1.207619 | 0.318180 | 0.750348 | |
| 258 | 30 | 7.200181 | 2.733309 | 0.006270 | HH |
| 259 | 20 | -1.802335 | -0.436611 | 0.662394 | |
| 260 | 0 | -2.606986 | -1.303789 | 0.192306 | |
| 261 | 0 | 4.704028 | 1.928409 | 0.053804 | |

| | | | | | |
|---|---|---|---|---|---|
| 262 | 10 | 3.448023 | 1.729196 | 0.083774 | |
| 263 | 30 | -8.999985 | -2.418371 | 0.015590 | HL |
| 264 | 0 | 14.476135 | 3.197614 | 0.001386 | LL |
| 265 | 30 | -12.034326 | -3.355687 | 0.000792 | HL |
| 266 | 0 | -10.151156 | -3.073947 | 0.002112 | LH |
| 267 | 20 | -0.232299 | -0.132505 | 0.894585 | |
| 268 | 30 | 5.523637 | 1.607657 | 0.107910 | |
| 269 | 30 | 2.972711 | 0.998375 | 0.318097 | |
| 270 | 30 | 8.300864 | 2.096139 | 0.036070 | HH |
| 271 | 30 | 8.496348 | 1.878718 | 0.060283 | |
| 272 | 0 | -13.442612 | -3.748815 | 0.000178 | LH |
| 273 | 30 | 9.010578 | 2.348115 | 0.018869 | HH |
| 274 | 0 | 8.723586 | 2.775515 | 0.005511 | LL |
| 276 | 30 | -2.705003 | -0.657425 | 0.510908 | |
| 277 | 30 | -8.609016 | -1.773865 | 0.076086 | |
| 278 | 30 | 4.104209 | 1.108485 | 0.267652 | |
| 279 | 30 | 5.266522 | 1.374101 | 0.169410 | |
| 280 | 30 | 3.713240 | 1.862044 | 0.062597 | |
| 281 | 30 | -13.741685 | -2.957844 | 0.003098 | HL |
| 282 | 30 | 10.430006 | 2.915310 | 0.003553 | HH |
| 283 | 30 | 1.779582 | 0.520381 | 0.602798 | |
| 284 | 30 | 1.326983 | 0.424971 | 0.670858 | |
| 285 | 20 | 0.132180 | 0.078199 | 0.937670 | |
| 286 | 30 | 0.678899 | 0.394255 | 0.693393 | |
| 287 | 0 | 0.407682 | 0.157342 | 0.874975 | |
| 288 | 30 | 3.939540 | 1.768226 | 0.077023 | |
| 289 | 30 | -1.645728 | -1.645126 | 0.099944 | |
| 290 | 30 | 6.943066 | 2.209689 | 0.027127 | HH |
| 291 | 30 | -2.159958 | -0.816402 | 0.414270 | |
| 292 | 20 | -0.785018 | -0.294974 | 0.768014 | |
| 293 | 30 | -3.291456 | -2.327760 | 0.019925 | HL |
| 294 | 30 | 4.649254 | 2.330898 | 0.019759 | HH |
| 295 | 30 | 4.649254 | 2.330898 | 0.019759 | HH |
| 296 | 30 | -1.676543 | -0.749210 | 0.453730 | |
| 297 | 30 | 4.104209 | 1.108485 | 0.267652 | |
| 298 | 10 | 0.747346 | 0.286155 | 0.774759 | |
| 299 | 30 | -0.483415 | -0.340633 | 0.733380 | |
| 300 | 30 | 3.877909 | 1.086269 | 0.277360 | |
| 301 | 30 | -0.483415 | -0.340633 | 0.733380 | |
| 302 | 30 | 3.486941 | 2.017574 | 0.043636 | HH |
| 303 | 30 | 1.584098 | 0.603482 | 0.546188 | |
| 304 | 10 | 2.675393 | 0.581704 | 0.560766 | |
| 306 | 30 | -0.483415 | -0.340633 | 0.733380 | |
| 307 | 30 | -8.969170 | -2.847013 | 0.004413 | HL |
| 308 | 30 | -7.189588 | -1.545202 | 0.122297 | |
| 309 | 30 | -7.642187 | -1.721619 | 0.085139 | |
| 310 | 10 | -0.238727 | -0.072592 | 0.942131 | |
| 311 | 10 | 0.452171 | 0.204996 | 0.837575 | |
| 313 | 30 | 0.390969 | 0.127518 | 0.898531 | |
| 314 | 20 | -2.260934 | -0.519176 | 0.603638 | |
| 315 | 10 | 5.080895 | 1.247190 | 0.212328 | |
| 316 | 10 | 5.080895 | 1.247190 | 0.212328 | |
| 317 | 30 | 2.715596 | 0.792200 | 0.428244 | |
| 318 | 30 | -8.094787 | -1.920985 | 0.054734 | |
| 319 | 30 | 1.584098 | 0.603482 | 0.546188 | |
| 320 | 30 | -6.387428 | -2.135426 | 0.032726 | HL |
| 321 | 10 | 14.363628 | 3.624078 | 0.000290 | LL |
| 322 | 0 | 34.952974 | 9.097011 | 0.000000 | LL |
| 323 | 0 | 34.952974 | 9.097011 | 0.000000 | LL |
| 324 | 0 | 34.952974 | 9.097011 | 0.000000 | LL |
| 325 | 30 | 1.131498 | 0.509508 | 0.610396 | |
| 326 | 30 | 2.067512 | 0.929080 | 0.352847 | |

| | | | | | |
|---|---|---|---|---|---|
| 327 | 30 | 4.392139 | 1.668396 | 0.095237 | |
| 328 | 10 | 7.065390 | 1.096832 | 0.272715 | |
| 329 | 30 | -8.248863 | -1.358284 | 0.174374 | |
| 330 | 30 | -8.248863 | -1.358284 | 0.174374 | |
| 331 | 0 | 28.646849 | 7.713879 | 0.000000 | LL |
| 332 | 0 | 26.316651 | 7.350123 | 0.000000 | LL |
| 333 | 20 | -0.320419 | -0.319473 | 0.749368 | |
| 334 | 0 | 6.670175 | 3.343188 | 0.000828 | LL |
| 335 | 30 | 0.617268 | 0.190563 | 0.848868 | |
| 336 | 30 | -3.774871 | -1.888788 | 0.058920 | |
| 337 | 20 | -0.326419 | -0.144011 | 0.885492 | |
| 338 | 30 | 1.069868 | 0.302395 | 0.762351 | |
| 339 | 30 | -8.156417 | -1.615715 | 0.106156 | |
| 340 | 30 | -11.828249 | -1.760954 | 0.078246 | |
| 341 | 30 | 11.139721 | 3.238572 | 0.001201 | HH |
| 342 | 30 | 11.139721 | 3.238572 | 0.001201 | HH |
| 343 | 30 | 4.135024 | 1.317330 | 0.187728 | |
| 344 | 30 | -1.902843 | -0.951080 | 0.341564 | |
| 345 | 30 | 4.135024 | 1.317330 | 0.187728 | |
| 346 | 30 | -4.001170 | -2.311277 | 0.020818 | HL |
| 347 | 30 | -14.605476 | -2.270819 | 0.023158 | HL |
| 348 | 30 | -9.905184 | -3.144466 | 0.001664 | HL |
| 349 | 0 | 9.044005 | 3.031062 | 0.002437 | LL |
| 350 | 0 | 9.044005 | 3.031062 | 0.002437 | LL |
| 351 | 10 | 3.605019 | 1.369891 | 0.170721 | |
| 352 | 10 | 2.028595 | 0.833058 | 0.404812 | |
| 353 | 10 | 5.909973 | 1.795077 | 0.072641 | |
| 354 | 10 | 5.909973 | 1.795077 | 0.072641 | |
| 355 | 10 | 2.185591 | 0.734845 | 0.462434 | |
| 356 | 0 | 11.650991 | 5.224920 | 0.000000 | LL |
| 357 | 30 | 2.324627 | 1.646493 | 0.099662 | |
| 358 | 30 | -0.257115 | -0.146851 | 0.883249 | |
| 359 | 20 | -0.414539 | -0.237857 | 0.811992 | |
| 360 | 20 | -0.100119 | -0.038460 | 0.969321 | |
| 361 | 30 | 1.614913 | 0.935362 | 0.349602 | |
| 362 | 30 | -0.030815 | -0.013372 | 0.989331 | |
| 363 | 30 | 2.324627 | 1.646493 | 0.099662 | |
| 364 | 30 | 1.131498 | 0.509508 | 0.610396 | |
| 365 | 30 | 1.614913 | 0.935362 | 0.349602 | |
| 366 | 30 | 5.328153 | 2.023367 | 0.043035 | HH |
| 367 | 30 | 1.614913 | 0.935362 | 0.349602 | |
| 368 | 30 | 3.939540 | 1.768226 | 0.077023 | |
| 369 | 30 | 3.486941 | 2.017574 | 0.043636 | HH |
| 370 | 30 | 10.460822 | 3.505417 | 0.000456 | HH |
| 371 | 30 | 5.101853 | 2.091283 | 0.036503 | HH |
| 372 | 30 | 10.460822 | 3.505417 | 0.000456 | HH |
| 373 | 30 | 1.388613 | 0.984117 | 0.325058 | |
| 374 | 30 | 1.388613 | 0.984117 | 0.325058 | |
| 375 | 30 | -0.966829 | -0.482226 | 0.629645 | |
| 376 | 30 | 1.388613 | 0.984117 | 0.325058 | |
| 377 | 30 | 8.136195 | 3.088280 | 0.002013 | HH |
| 378 | 30 | 8.136195 | 3.088280 | 0.002013 | HH |
| 379 | 30 | 3.486941 | 2.017574 | 0.043636 | HH |
| 380 | 30 | 5.811568 | 2.607371 | 0.009124 | HH |
| 381 | 30 | 5.811568 | 2.607371 | 0.009124 | HH |
| 382 | 30 | 2.067512 | 0.929080 | 0.352847 | |
| 383 | 30 | 2.067512 | 0.929080 | 0.352847 | |
| 384 | 30 | 2.324627 | 1.646493 | 0.099662 | |
| 385 | 30 | 4.844738 | 1.625136 | 0.104134 | |
| 386 | 30 | 8.362494 | 2.971006 | 0.002968 | HH |
| 387 | 30 | 6.973881 | 2.857711 | 0.004267 | HH |
| 388 | 30 | 6.973881 | 2.857711 | 0.004267 | HH |

| | | | | | |
|---|---|---|---|---|---|
| 389 | 30 | 6.973881 | 2.857711 | 0.004267 | HH |
| 390 | 30 | 1.162314 | 1.163646 | 0.244567 | |
| 391 | 30 | 1.614913 | 0.935362 | 0.349602 | |
| 392 | 30 | 0.452599 | 0.321742 | 0.747648 | |
| 393 | 30 | 16.632543 | 3.194271 | 0.001402 | HH |
| 394 | 30 | 0.452599 | 0.321742 | 0.747648 | |
| 395 | 30 | 11.726174 | 2.293575 | 0.021815 | HH |
| 396 | 30 | 11.078090 | 2.507050 | 0.012174 | HH |
| 398 | 30 | 1.162314 | 1.163646 | 0.244567 | |
| 399 | 30 | 4.875554 | 2.187798 | 0.028684 | HH |
| 400 | 30 | 0.226300 | 0.227389 | 0.820121 | |
| 401 | 30 | 5.040223 | 1.360403 | 0.173702 | |
| 402 | 30 | 6.685951 | 1.870144 | 0.061464 | |
| 403 | 30 | 6.685951 | 1.870144 | 0.061464 | |
| 404 | 30 | 9.010578 | 2.348115 | 0.018869 | HH |
| 405 | 30 | 2.324627 | 1.646493 | 0.099662 | |
| 406 | 30 | 6.973881 | 2.857711 | 0.004267 | HH |
| 407 | 30 | -0.709714 | -0.708869 | 0.478406 | |
| 408 | 30 | -3.867317 | -0.970489 | 0.331803 | |
| 409 | 30 | 0.874383 | 0.313267 | 0.754078 | |
| 410 | 30 | -0.287930 | -0.106460 | 0.915218 | |
| 411 | 30 | -0.483415 | -0.340633 | 0.733380 | |
| 412 | 30 | 1.162314 | 1.163646 | 0.244567 | |
| 413 | 30 | 4.875554 | 2.187798 | 0.028684 | HH |
| 414 | 30 | 3.713240 | 1.862044 | 0.062597 | |
| 415 | 30 | 0.452599 | 0.321742 | 0.747648 | |
| 416 | 30 | 1.614913 | 0.935362 | 0.349602 | |
| 417 | 30 | 0.452599 | 0.321742 | 0.747648 | |
| 419 | 30 | 1.162314 | 1.163646 | 0.244567 | |
| 420 | 30 | 1.614913 | 0.935362 | 0.349602 | |
| 421 | 30 | 0.452599 | 0.321742 | 0.747648 | |
| 422 | 30 | 1.841212 | 0.924336 | 0.355311 | |
| 423 | 30 | 0.226300 | 0.227389 | 0.820121 | |
| 424 | 30 | 1.162314 | 1.163646 | 0.244567 | |
| 425 | 30 | 2.324627 | 1.646493 | 0.099662 | |
| 426 | 30 | 2.324627 | 1.646493 | 0.099662 | |
| 427 | 30 | 1.162314 | 1.163646 | 0.244567 | |
| 428 | 30 | 1.553282 | 0.474321 | 0.635271 | |
| 429 | 30 | 3.003526 | 1.348653 | 0.177448 | |
| 430 | 30 | 3.003526 | 1.348653 | 0.177448 | |
| 431 | 30 | -0.257115 | -0.146851 | 0.883249 | |
| 432 | 30 | 2.520111 | 0.958453 | 0.337834 | |
| 433 | 30 | 3.682425 | 1.309919 | 0.190223 | |
| 434 | 30 | 5.101853 | 2.091283 | 0.036503 | HH |
| 435 | 30 | 2.324627 | 1.646493 | 0.099662 | |
| 436 | 30 | 2.324627 | 1.646493 | 0.099662 | |
| 437 | 30 | 4.649254 | 2.330898 | 0.019759 | HH |
| 438 | 30 | 2.324627 | 1.646493 | 0.099662 | |
| 440 | 30 | 1.388613 | 0.984117 | 0.325058 | |
| 441 | 30 | -0.030815 | -0.013372 | 0.989331 | |
| 442 | 30 | -4.937184 | -2.852383 | 0.004339 | HL |
| 444 | 30 | 1.162314 | 1.163646 | 0.244567 | |
| 446 | 30 | 0.452599 | 0.321742 | 0.747648 | |
| 447 | 30 | 0.452599 | 0.321742 | 0.747648 | |
| 448 | 30 | 1.162314 | 1.163646 | 0.244567 | |
| 449 | 30 | 1.162314 | 1.163646 | 0.244567 | |
| 450 | 30 | -0.709714 | -0.708869 | 0.478406 | |
| 451 | 30 | 0.195484 | 0.089935 | 0.928339 | |
| 452 | 30 | -0.514230 | -0.208001 | 0.835228 | |
| 453 | 30 | 4.392139 | 1.668396 | 0.095237 | |
| 454 | 30 | 5.101853 | 2.091283 | 0.036503 | HH |
| 455 | 30 | 3.939540 | 1.768226 | 0.077023 | |

| | | | | | |
|---|---|---|---|---|---|
| 456 | 30 | -2.869672 | -1.015602 | 0.309819 | |
| 457 | 30 | 1.614913 | 0.935362 | 0.349602 | |
| 458 | 30 | 6.264167 | 2.378338 | 0.017391 | HH |
| 459 | 30 | 1.357798 | 0.558427 | 0.576553 | |
| 460 | 30 | 2.550927 | 1.476468 | 0.139818 | |
| 461 | 30 | 2.550927 | 1.476468 | 0.139818 | |
| 462 | 30 | -0.709714 | -0.708869 | 0.478406 | |
| 463 | 30 | -2.355442 | -1.665384 | 0.095836 | |
| 466 | 30 | 1.388613 | 0.984117 | 0.325058 | |
| 467 | 30 | 2.550927 | 1.476468 | 0.139818 | |
| 468 | 30 | 1.162314 | 1.163646 | 0.244567 | |
| 469 | 30 | -1.419428 | -1.003009 | 0.315857 | |
| 470 | 30 | 3.003526 | 1.348653 | 0.177448 | |
| 471 | 30 | 3.713240 | 1.862044 | 0.062597 | |
| 472 | 30 | 2.324627 | 1.646493 | 0.099662 | |
| 473 | 0 | 1.048521 | 0.472313 | 0.636704 | |
| 474 | 20 | 0.949259 | 0.427818 | 0.668784 | |
| 477 | 20 | 0.496659 | 0.288903 | 0.772656 | |
| 478 | 20 | -0.232299 | -0.132505 | 0.894585 | |
| 479 | 0 | 1.004889 | 1.006181 | 0.314328 | |
| 480 | 20 | 0.226300 | 0.227389 | 0.820121 | |
| 481 | 0 | -3.611875 | -2.086227 | 0.036958 | LH |
| 482 | 0 | 0.364051 | 0.212243 | 0.831918 | |
| 483 | 20 | -0.188239 | -0.092227 | 0.926518 | |
| 484 | 0 | -1.645728 | -1.645126 | 0.099944 | |
| 485 | 0 | 34.952974 | 9.097011 | 0.000000 | LL |
| 486 | 0 | 34.952974 | 9.097011 | 0.000000 | LL |
| 488 | 0 | 2.009779 | 1.423689 | 0.154537 | |
| 489 | 10 | 1.438244 | 1.019239 | 0.308089 | |
| 492 | 10 | 1.438244 | 1.019239 | 0.308089 | |
| 493 | 10 | 0.433355 | 0.434498 | 0.663927 | |
| 494 | 0 | 5.665286 | 3.276869 | 0.001050 | LL |
| 495 | 0 | 2.330198 | 2.331835 | 0.019709 | LL |
| 496 | 10 | 0.866710 | 0.614790 | 0.538694 | |
| 499 | 10 | 0.433355 | 0.434498 | 0.663927 | |
| 500 | 10 | 0.433355 | 0.434498 | 0.663927 | |
| 503 | 10 | 1.300065 | 0.753349 | 0.451240 | |
| 504 | 10 | 1.733420 | 0.870342 | 0.384114 | |
| 505 | 10 | 0.866710 | 0.614790 | 0.538694 | |
| 506 | 20 | -0.320419 | -0.319473 | 0.749368 | |
| 507 | 30 | 2.777226 | 1.393190 | 0.163562 | |
| 509 | 0 | 3.058300 | 1.162554 | 0.245010 | |
| 510 | 0 | -12.758142 | -3.315003 | 0.000916 | LH |
| 511 | 0 | 0.451314 | 0.140253 | 0.888460 | |
| 512 | 0 | 0.451314 | 0.140253 | 0.888460 | |
| 513 | 10 | 0.175812 | 0.069408 | 0.944665 | |
| 514 | 10 | -3.649119 | -0.978241 | 0.327955 | |
| 515 | 0 | 5.475761 | 1.384156 | 0.166311 | |
| 516 | 10 | 5.074427 | 0.773511 | 0.439220 | |
| 517 | 10 | -0.948442 | -0.284091 | 0.776340 | |
| 518 | 10 | 6.770215 | 1.074558 | 0.282573 | |
| 519 | 10 | 3.699099 | 0.727238 | 0.467080 | |
| 520 | 10 | -1.030173 | -0.207768 | 0.835410 | |
| 522 | 10 | -7.474049 | -1.643700 | 0.100238 | |
| 523 | 10 | 4.546993 | 0.926311 | 0.354284 | |
| 524 | 20 | 0.358480 | 0.181628 | 0.855875 | |
| 525 | 10 | 5.080895 | 1.247190 | 0.212328 | |
| 526 | 10 | 1.614056 | 0.543494 | 0.586790 | |
| 527 | 20 | 0.126180 | 0.050586 | 0.959655 | |
| 528 | 10 | -0.967258 | -0.364086 | 0.715794 | |
| 529 | 10 | -0.829078 | -0.336903 | 0.736190 | |
| 530 | 10 | -0.138180 | -0.137185 | 0.890884 | |

| | | | | | |
|---|---|---|---|---|---|
| 531 | 20 | -1.638094 | -0.303123 | 0.761796 | |
| 532 | 0 | -0.320419 | -0.319473 | 0.749368 | |
| 533 | 20 | 0.220300 | 0.101059 | 0.919504 | |
| 534 | 10 | 1.218333 | 0.311197 | 0.755651 | |
| 535 | 10 | 0.728530 | 0.422947 | 0.672334 | |
| 536 | 10 | 3.071116 | 0.802968 | 0.421993 | |
| 537 | 0 | 2.330198 | 2.331835 | 0.019709 | LL |
| 538 | 10 | 5.966421 | 1.263755 | 0.206318 | |
| 539 | 10 | 4.546993 | 0.926311 | 0.354284 | |
| 540 | 20 | 0.678899 | 0.394255 | 0.693393 | |
| 541 | 10 | -0.276359 | -0.194110 | 0.846090 | |
| 542 | 20 | 0.452599 | 0.321742 | 0.747648 | |
| 543 | 10 | -1.262433 | -0.563583 | 0.573038 | |
| 544 | 20 | 0.678899 | 0.394255 | 0.693393 | |
| 546 | 20 | 1.131498 | 0.509508 | 0.610396 | |
| 547 | 30 | 1.388613 | 0.984117 | 0.325058 | |
| 548 | 10 | -3.391575 | -1.200840 | 0.229813 | |
| 549 | 10 | 0.866710 | 0.614790 | 0.538694 | |
| 550 | 20 | -0.414539 | -0.237857 | 0.811992 | |
| 551 | 10 | 0.452171 | 0.204996 | 0.837575 | |
| 552 | 30 | -1.419428 | -1.003009 | 0.315857 | |
| 554 | 10 | 0.295175 | 0.210340 | 0.833402 | |
| 556 | 10 | -1.796335 | -0.497716 | 0.618684 | |
| 557 | 0 | -5.490760 | -1.529027 | 0.126258 | |
| 558 | 0 | 1.368940 | 0.687773 | 0.491596 | |
| 559 | 10 | -0.119364 | -0.051197 | 0.959168 | |
| 560 | 0 | -3.291456 | -2.327760 | 0.019925 | LH |
| 562 | 30 | -3.805686 | -1.347819 | 0.177716 | |
| 563 | 30 | 1.388613 | 0.984117 | 0.325058 | |
| 564 | 30 | 2.324627 | 1.646493 | 0.099662 | |
| 565 | 30 | 2.324627 | 1.646493 | 0.099662 | |
| 566 | 30 | 3.713240 | 1.862044 | 0.062597 | |
| 567 | 30 | 3.939540 | 1.768226 | 0.077023 | |
| 568 | 30 | 1.614913 | 0.935362 | 0.349602 | |
| 569 | 30 | -0.257115 | -0.146851 | 0.883249 | |
| 570 | 30 | 0.678899 | 0.394255 | 0.693393 | |
| 571 | 30 | -9.905184 | -3.144466 | 0.001664 | HL |
| 572 | 30 | 11.139721 | 3.238572 | 0.001201 | HH |
| 573 | 30 | -1.419428 | -1.003009 | 0.315857 | |
| 574 | 30 | 1.614913 | 0.935362 | 0.349602 | |
| 575 | 30 | 0.195484 | 0.089935 | 0.928339 | |
| 576 | 30 | 5.554453 | 1.974354 | 0.048342 | HH |
| 577 | 30 | 3.939540 | 1.768226 | 0.077023 | |
| 578 | 30 | -0.740529 | -0.329638 | 0.741674 | |
| 579 | 30 | 1.614913 | 0.935362 | 0.349602 | |
| 580 | 30 | 5.554453 | 1.974354 | 0.048342 | HH |
| 581 | 30 | 2.067512 | 0.929080 | 0.352847 | |
| 582 | 0 | 1.004889 | 1.006181 | 0.314328 | |
| 583 | 0 | -3.932295 | -1.967643 | 0.049109 | LH |
| 585 | 0 | 2.373830 | 1.066389 | 0.286248 | |
| 586 | 0 | 4.019558 | 2.015480 | 0.043854 | LL |
| 587 | 0 | -2.286567 | -1.320070 | 0.186812 | |
| 588 | 10 | -0.986074 | -0.568260 | 0.569859 | |
| 589 | 0 | -4.937184 | -2.852383 | 0.004339 | LH |
| 590 | 0 | 9.044005 | 3.031062 | 0.002437 | LL |
| 591 | 0 | 10.048895 | 3.196681 | 0.001390 | LL |
| 592 | 20 | 0.452599 | 0.321742 | 0.747648 | |
| 593 | 10 | -2.838857 | -1.419934 | 0.155627 | |
| 594 | 30 | 2.777226 | 1.393190 | 0.163562 | |
| 595 | 20 | 0.767019 | 0.346128 | 0.729246 | |
| 596 | 10 | -0.276359 | -0.194110 | 0.846090 | |
| 597 | 10 | -0.276359 | -0.194110 | 0.846090 | |

| | | | | | |
|---|---|---|---|---|---|
| 598 | 10 | -2.267322 | -1.133650 | 0.256942 | |
| 599 | 20 | 0.402540 | 0.182749 | 0.854995 | |
| 600 | 10 | 1.004889 | 1.006181 | 0.314328 | |
| 601 | 20 | 0.949259 | 0.427818 | 0.668784 | |
| 602 | 20 | 0.044060 | 0.045102 | 0.964026 | |
| 603 | 20 | 0.044060 | 0.045102 | 0.964026 | |
| 604 | 20 | 0.314420 | 0.183551 | 0.854366 | |
| 606 | 20 | 0.132180 | 0.078199 | 0.937670 | |
| 608 | 10 | 0.646798 | 0.167159 | 0.867245 | |
| 609 | 0 | -0.640839 | -0.452035 | 0.651243 | |
| 610 | 10 | 1.300065 | 0.753349 | 0.451240 | |
| 611 | 10 | -0.709714 | -0.708869 | 0.478406 | |
| 612 | 0 | 2.330198 | 2.331835 | 0.019709 | LL |
| 614 | 20 | -0.558718 | -0.195380 | 0.845095 | |
| 615 | 10 | 2.600129 | 1.067050 | 0.285949 | |
| 616 | 20 | 1.357798 | 0.558427 | 0.576553 | |
| 617 | 10 | -1.262433 | -0.563583 | 0.573038 | |
| 618 | 10 | -1.262433 | -0.563583 | 0.573038 | |
| 619 | 10 | -1.262433 | -0.563583 | 0.573038 | |
| 620 | 10 | -1.262433 | -0.563583 | 0.573038 | |
| 621 | 10 | -1.262433 | -0.563583 | 0.573038 | |
| 623 | 10 | -0.986074 | -0.568260 | 0.569859 | |
| 624 | 10 | 0.590351 | 0.297773 | 0.765876 | |
| 626 | 10 | -0.847894 | -0.598559 | 0.549467 | |
| 627 | 10 | 1.004889 | 1.006181 | 0.314328 | |
| 628 | 0 | 2.330198 | 2.331835 | 0.019709 | LL |
| 629 | 10 | 2.185591 | 0.734845 | 0.462434 | |
| 630 | 10 | 3.605019 | 1.369891 | 0.170721 | |
| 631 | 20 | -0.552719 | -0.274796 | 0.783473 | |
| 632 | 10 | 5.909973 | 1.795077 | 0.072641 | |
| 633 | 10 | 5.909973 | 1.795077 | 0.072641 | |
| 634 | 10 | -2.681861 | -1.014327 | 0.310427 | |
| 635 | 10 | -5.244359 | -1.752723 | 0.079650 | |
| 637 | 20 | 0.088120 | 0.063816 | 0.949117 | |
| 638 | 20 | 0.358480 | 0.181628 | 0.855875 | |
| 639 | 30 | -11.417058 | -2.354120 | 0.018567 | HL |
| 640 | 10 | 1.218333 | 0.311197 | 0.755651 | |
| 641 | 0 | -3.568244 | -1.350476 | 0.176863 | |
| 642 | 30 | -13.607831 | -2.335930 | 0.019495 | HL |
| 643 | 10 | 2.888837 | 0.490495 | 0.623784 | |
| 644 | 10 | 3.893726 | 0.650684 | 0.515250 | |
| 645 | 10 | 3.699099 | 0.727238 | 0.467080 | |
| 646 | 10 | 5.488966 | 0.862911 | 0.388186 | |
| 647 | 10 | 3.699099 | 0.727238 | 0.467080 | |
| 648 | 0 | 6.480651 | 1.589603 | 0.111924 | |
| 649 | 10 | 6.336860 | 1.017698 | 0.308821 | |
| 650 | 10 | 7.348218 | 2.055018 | 0.039877 | LL |
| 651 | 10 | 1.928047 | 0.505599 | 0.613138 | |
| 652 | 20 | 2.339117 | 0.533029 | 0.594013 | |
| 653 | 10 | -6.921331 | -1.688838 | 0.091250 | |
| 654 | 10 | -7.474049 | -1.643700 | 0.100238 | |
| 655 | 10 | 0.313991 | 0.131081 | 0.895711 | |
| 656 | 10 | 0.784978 | 0.208230 | 0.835049 | |
| 657 | 20 | -0.696898 | -0.230220 | 0.817921 | |
| 658 | 10 | -0.810262 | -0.254218 | 0.799327 | |
| 659 | 20 | 0.088120 | 0.063816 | 0.949117 | |
| 660 | 10 | -1.124253 | -0.561081 | 0.574743 | |
| 662 | 20 | 1.389859 | 0.365590 | 0.714671 | |
| 663 | 20 | 0.032061 | 0.013837 | 0.988960 | |
| 664 | 10 | -8.221395 | -2.208822 | 0.027187 | LH |
| 665 | 30 | -10.481044 | -2.160701 | 0.030718 | HL |
| 666 | 10 | 5.495434 | 1.482919 | 0.138096 | |

| | | | | | |
|---|---|---|---|---|---|
| 667 | 10 | 5.080895 | 1.247190 | 0.212328 | |
| 668 | 10 | 5.080895 | 1.247190 | 0.212328 | |
| 669 | 30 | -3.065157 | -1.770170 | 0.076699 | |
| 670 | 10 | 7.210038 | 1.944386 | 0.051849 | |
| 671 | 30 | -0.318745 | -0.093195 | 0.925748 | |
| 672 | 10 | 0.646798 | 0.167159 | 0.867245 | |
| 673 | 10 | 0.646798 | 0.167159 | 0.867245 | |
| 674 | 10 | -5.225543 | -1.454991 | 0.145672 | |
| 675 | 30 | -5.091261 | -1.069125 | 0.285014 | |
| 676 | 30 | -11.643358 | -2.451444 | 0.014228 | HL |
| 677 | 20 | 2.709596 | 0.687026 | 0.492066 | |
| 678 | 10 | 0.175812 | 0.069408 | 0.944665 | |
| 679 | 10 | 1.890415 | 0.719649 | 0.471741 | |
| 680 | 10 | 0.156996 | 0.092545 | 0.926265 | |
| 681 | 20 | -0.288359 | -0.088364 | 0.929587 | |
| 682 | 10 | -2.838857 | -1.419934 | 0.155627 | |
| 683 | 20 | 1.075439 | 0.315897 | 0.752081 | |
| 684 | 10 | -0.276359 | -0.194110 | 0.846090 | |
| 685 | 20 | 0.126180 | 0.050586 | 0.959655 | |
| 686 | 10 | -2.663045 | -0.803883 | 0.421464 | |
| 687 | 0 | -4.209083 | -1.406110 | 0.159692 | |
| 688 | 10 | 0.433355 | 0.434498 | 0.663927 | |
| 690 | 10 | 0.728530 | 0.422947 | 0.672334 | |
| 692 | 10 | 0.866710 | 0.614790 | 0.538694 | |
| 694 | 10 | 1.300065 | 0.753349 | 0.451240 | |
| 695 | 10 | 1.300065 | 0.753349 | 0.451240 | |
| 696 | 10 | 0.866710 | 0.614790 | 0.538694 | |
| 697 | 10 | 0.433355 | 0.434498 | 0.663927 | |
| 698 | 10 | 1.300065 | 0.753349 | 0.451240 | |
| 699 | 10 | 1.004889 | 1.006181 | 0.314328 | |
| 700 | 0 | 2.009779 | 1.423689 | 0.154537 | |
| 701 | 10 | 1.004889 | 1.006181 | 0.314328 | |
| 702 | 10 | 0.433355 | 0.434498 | 0.663927 | |
| 703 | 10 | 0.433355 | 0.434498 | 0.663927 | |
| 704 | 10 | 0.433355 | 0.434498 | 0.663927 | |
| 705 | 10 | 0.433355 | 0.434498 | 0.663927 | |
| 706 | 10 | 0.728530 | 0.422947 | 0.672334 | |
| 707 | 10 | -1.419428 | -1.003009 | 0.315857 | |
| 709 | 10 | -1.796335 | -0.497716 | 0.618684 | |
| 710 | 0 | 1.368940 | 0.687773 | 0.491596 | |
| 711 | 10 | -4.377649 | -1.323675 | 0.185611 | |
| 712 | 10 | -4.377649 | -1.323675 | 0.185611 | |
| 713 | 10 | -1.953331 | -0.617471 | 0.536924 | |
| 714 | 0 | -0.320419 | -0.319473 | 0.749368 | |
| 715 | 0 | 14.476135 | 3.197614 | 0.001386 | LL |
| 716 | 0 | 3.014668 | 1.744556 | 0.081062 | |
| 717 | 0 | 6.713807 | 2.385841 | 0.017040 | LL |
| 718 | 10 | -0.986074 | -0.568260 | 0.569859 | |
| 719 | 30 | -3.095972 | -1.171373 | 0.241449 | |
| 720 | 0 | 16.398651 | 4.270117 | 0.000020 | LL |
| 721 | 10 | -4.220653 | -1.132064 | 0.257608 | |
| 722 | 10 | -4.220653 | -1.132064 | 0.257608 | |
| 723 | 10 | -0.119364 | -0.051197 | 0.959168 | |
| 724 | 10 | -2.977037 | -1.332163 | 0.182807 | |
| 725 | 20 | 0.767019 | 0.346128 | 0.729246 | |
| 726 | 0 | 6.437019 | 1.800654 | 0.071757 | |
| 727 | 10 | -3.391575 | -1.200840 | 0.229813 | |
| 728 | 10 | -2.248506 | -0.795133 | 0.426536 | |
| 729 | 10 | -2.248506 | -0.795133 | 0.426536 | |
| 730 | 10 | -0.829078 | -0.336903 | 0.736190 | |
| 731 | 10 | 0.728530 | 0.422947 | 0.672334 | |
| 733 | 20 | -0.320419 | -0.319473 | 0.749368 | |

| | | | | | |
|---|---|---|---|---|---|
| 734 | 10 | -0.709714 | -0.708869 | 0.478406 | |
| 735 | 20 | 0.452599 | 0.321742 | 0.747648 | |
| 736 | 30 | 0.452599 | 0.321742 | 0.747648 | |
| 737 | 30 | 11.139721 | 3.238572 | 0.001201 | HH |
| 738 | 10 | 5.099711 | 1.129558 | 0.258662 | |
| 739 | 0 | 10.456577 | 2.562209 | 0.010401 | LL |
| 740 | 20 | -2.202812 | -0.344693 | 0.730325 | |
| 741 | 10 | 0.433355 | 0.434498 | 0.663927 | |
| 742 | 10 | 3.893726 | 0.650684 | 0.515250 | |
| 743 | 10 | 3.027016 | 0.520283 | 0.602867 | |
| 744 | 10 | 6.789031 | 1.032456 | 0.301859 | |
| 745 | 0 | 8.767218 | 2.363485 | 0.018104 | LL |
| 746 | 0 | 7.718696 | 2.587345 | 0.009672 | LL |
| 747 | 0 | 17.403540 | 4.390198 | 0.000011 | LL |
| 748 | 30 | -6.253574 | -1.343396 | 0.179144 | |
| 750 | 10 | 3.900194 | 1.308900 | 0.190568 | |
| 751 | 0 | 6.713807 | 2.385841 | 0.017040 | LL |
| 752 | 0 | 11.097416 | 2.891004 | 0.003840 | LL |
| 753 | 10 | 1.752236 | 0.624841 | 0.532075 | |
| 754 | 30 | -0.092446 | -0.023257 | 0.981445 | |
| 755 | 10 | -1.086621 | -0.311967 | 0.755066 | |
| 756 | 10 | 6.336860 | 1.017698 | 0.308821 | |
| 757 | 10 | 7.442297 | 1.323800 | 0.185569 | |
| 758 | 0 | 0.364051 | 0.212243 | 0.831918 | |
| 759 | 0 | 0.494946 | 0.132779 | 0.894369 | |
| 760 | 10 | 0.784978 | 0.208230 | 0.835049 | |
| 761 | 20 | 0.126180 | 0.050586 | 0.959655 | |
| 762 | 0 | 3.014668 | 1.744556 | 0.081062 | |
| 763 | 10 | 1.890415 | 0.719649 | 0.471741 | |
| 764 | 0 | 5.344867 | 2.679334 | 0.007377 | LL |
| 765 | 0 | 4.339977 | 2.510712 | 0.012049 | LL |
| 766 | 20 | 1.075439 | 0.315897 | 0.752081 | |
| 767 | 0 | 39.613370 | 9.694649 | 0.000000 | LL |
| 769 | 10 | 0.885526 | 0.365073 | 0.715057 | |
| 770 | 10 | 0.728530 | 0.422947 | 0.672334 | |
| 771 | 10 | -4.220653 | -1.132064 | 0.257608 | |
| 772 | 10 | -4.220653 | -1.132064 | 0.257608 | |
| 773 | 10 | 5.966421 | 1.263755 | 0.206318 | |
| 774 | 10 | 2.443134 | 1.414153 | 0.157317 | |
| 775 | 10 | 0.433355 | 0.434498 | 0.663927 | |
| 776 | 0 | 34.952974 | 9.097011 | 0.000000 | LL |
| 777 | 10 | 0.156996 | 0.092545 | 0.926265 | |
| 778 | 10 | -1.815151 | -0.604615 | 0.545435 | |
| 779 | 0 | 3.335088 | 2.361551 | 0.018199 | LL |
| 780 | 0 | 31.981937 | 8.064249 | 0.000000 | LL |
| 781 | 0 | 31.981937 | 8.064249 | 0.000000 | LL |
| 782 | 10 | 0.175812 | 0.069408 | 0.944665 | |
| 783 | 0 | 0.174526 | 0.048137 | 0.961607 | |
| 784 | 0 | 39.613370 | 9.694649 | 0.000000 | LL |
| 785 | 0 | 39.613370 | 9.694649 | 0.000000 | LL |
| 786 | 30 | -8.742870 | -2.647017 | 0.008121 | HL |
| 787 | 0 | 9.451688 | 2.386170 | 0.017025 | LL |
| 788 | 10 | -0.847894 | -0.598559 | 0.549467 | |
| 789 | 20 | -1.287677 | -0.454108 | 0.649751 | |
| 790 | 10 | 0.175812 | 0.069408 | 0.944665 | |
| 792 | 0 | 31.981937 | 8.064249 | 0.000000 | LL |
| 793 | 30 | 11.139721 | 3.238572 | 0.001201 | HH |
| 794 | 0 | 16.995858 | 5.693366 | 0.000000 | LL |
| 795 | 0 | 8.344537 | 1.423198 | 0.154679 | |
| 796 | 10 | 1.871599 | 1.083751 | 0.278475 | |
| 797 | 0 | -1.281677 | -0.639935 | 0.522215 | |
| 798 | 10 | 4.057190 | 1.181800 | 0.237285 | |

| | | | | | |
|---|---|---|---|---|---|
| 799 | 10 | 6.399776 | 1.327561 | 0.184323 | |
| 800 | 0 | 9.990265 | 1.725695 | 0.084402 | |
| 801 | 0 | 6.698809 | 1.128967 | 0.258912 | |
| 802 | 0 | -9.830737 | -3.120808 | 0.001804 | LH |
| 803 | 10 | 5.985237 | 1.173336 | 0.240661 | |
| 804 | 10 | 1.890415 | 0.719649 | 0.471741 | |
| 805 | 10 | -0.967258 | -0.364086 | 0.715794 | |
| 806 | 10 | 0.508619 | 0.128703 | 0.897593 | |
| 807 | 0 | 10.456577 | 2.562209 | 0.010401 | LL |
| 808 | 0 | 11.359205 | 1.862666 | 0.062509 | |
| 809 | 10 | 2.304954 | 1.156627 | 0.247425 | |
| 810 | 20 | -2.084694 | -0.434818 | 0.663694 | |
| 811 | 30 | -6.253574 | -1.343396 | 0.179144 | |
| 812 | 10 | -1.105437 | -0.389426 | 0.696961 | |
| 813 | 10 | 1.004889 | 1.006181 | 0.314328 | |
| 814 | 0 | 34.952974 | 9.097011 | 0.000000 | LL |
| 815 | 20 | 1.301739 | 0.367122 | 0.713528 | |
| 816 | 30 | 5.266522 | 1.374101 | 0.169410 | |
| 817 | 10 | 1.890415 | 0.719649 | 0.471741 | |
| 818 | 10 | 1.004889 | 1.006181 | 0.314328 | |
| 819 | 10 | -0.709714 | -0.708869 | 0.478406 | |
| 820 | 0 | 26.316651 | 7.350123 | 0.000000 | LL |
| 821 | 0 | 28.646849 | 7.713879 | 0.000000 | LL |
| 822 | 20 | -1.099438 | -0.548650 | 0.583245 | |
| 824 | 10 | 0.433355 | 0.434498 | 0.663927 | |
| 825 | 10 | 0.313991 | 0.131081 | 0.895711 | |
| 826 | 10 | -0.257543 | -0.102911 | 0.918033 | |
| 827 | 0 | -10.835626 | -3.624696 | 0.000289 | LH |
| 828 | 10 | -3.548571 | -1.588356 | 0.112206 | |
| 829 | 10 | 2.600129 | 1.067050 | 0.285949 | |
| 830 | 10 | -1.419428 | -1.003009 | 0.315857 | |
| 831 | 20 | -1.940515 | -0.457154 | 0.647560 | |
| 832 | 0 | 2.097042 | 0.669686 | 0.503058 | |
| 833 | 10 | 3.605019 | 1.369891 | 0.170721 | |
| 834 | 0 | 11.374204 | 3.617847 | 0.000297 | LL |
| 835 | 0 | 12.379093 | 3.756227 | 0.000172 | LL |
| 836 | 10 | 2.028595 | 0.833058 | 0.404812 | |
| 837 | 30 | 1.069868 | 0.302395 | 0.762351 | |
| 838 | 10 | -1.519976 | -0.457355 | 0.647416 | |
| 839 | 20 | 0.943259 | 0.318909 | 0.749795 | |
| 840 | 20 | 0.855139 | 0.327034 | 0.743642 | |
| 841 | 0 | 28.646849 | 7.713879 | 0.000000 | LL |
| 842 | 0 | 1.048521 | 0.472313 | 0.636704 | |
| 843 | 0 | 4.339977 | 2.510712 | 0.012049 | LL |
| 844 | 0 | 4.339977 | 2.510712 | 0.012049 | LL |
| 845 | 0 | 13.514877 | 2.797829 | 0.005145 | LL |
| 846 | 0 | 10.310684 | 1.805700 | 0.070965 | |
| 847 | 0 | 11.417835 | 3.076871 | 0.002092 | LL |
| 848 | 30 | 4.875554 | 2.187798 | 0.028684 | HH |
| 849 | 0 | -2.606986 | -1.303789 | 0.192306 | |
| 850 | 0 | 4.704028 | 1.928409 | 0.053804 | |
| 851 | 30 | -7.354257 | -2.049229 | 0.040440 | HL |
| 852 | 0 | 17.126753 | 3.782232 | 0.000155 | LL |
| 853 | 30 | 9.010578 | 2.348115 | 0.018869 | HH |
| 854 | 0 | -15.729179 | -3.959919 | 0.000075 | LH |
| 855 | 0 | -13.442612 | -3.748815 | 0.000178 | LH |
| 856 | 0 | 8.723586 | 2.775515 | 0.005511 | LL |
| 857 | 30 | -3.445533 | -0.737977 | 0.460528 | |
| 858 | 20 | 1.075439 | 0.315897 | 0.752081 | |
| 859 | 20 | 2.439236 | 0.660375 | 0.509013 | |
| 860 | 10 | -0.829078 | -0.336903 | 0.736190 | |
| 861 | 0 | -8.185009 | -2.737261 | 0.006195 | LH |

| | | | | | |
|---|---|---|---|---|---|
| 862 | 20 | 0.270360 | 0.192779 | 0.847132 | |
| 863 | 20 | 0.496659 | 0.288903 | 0.772656 | |
| 864 | 20 | 1.081439 | 0.386757 | 0.698936 | |
| 865 | 20 | -0.238299 | -0.087638 | 0.930165 | |
| 866 | 20 | -0.785018 | -0.294974 | 0.768014 | |
| 867 | 30 | -0.514230 | -0.208001 | 0.835228 | |
| 868 | 10 | -0.119364 | -0.051197 | 0.959168 | |
| 869 | 20 | -0.138180 | -0.137185 | 0.890884 | |
| 870 | 10 | 0.747346 | 0.286155 | 0.774759 | |
| 871 | 20 | 1.471979 | 0.329429 | 0.741832 | |
| 872 | 0 | -0.320419 | -0.319473 | 0.749368 | |
| 873 | 10 | 3.900194 | 1.308900 | 0.190568 | |
| 874 | 20 | -1.564036 | -0.493758 | 0.621477 | |
| 875 | 10 | -1.105437 | -0.389426 | 0.696961 | |
| 876 | 10 | -4.220653 | -1.132064 | 0.257608 | |
| 877 | 20 | -1.890455 | -0.487782 | 0.625704 | |
| 878 | 10 | -2.110327 | -0.797580 | 0.425114 | |
| 879 | 10 | 2.757125 | 0.926197 | 0.354344 | |
| 880 | 0 | 34.952974 | 9.097011 | 0.000000 | LL |
| 881 | 0 | 34.952974 | 9.097011 | 0.000000 | LL |
| 882 | 0 | 34.952974 | 9.097011 | 0.000000 | LL |
| 883 | 10 | 2.474298 | 0.406081 | 0.684683 | |
| 884 | 10 | 3.755547 | 0.620222 | 0.535112 | |
| 885 | 0 | 28.646849 | 7.713879 | 0.000000 | LL |
| 886 | 20 | 0.176240 | 0.090343 | 0.928015 | |
| 887 | 10 | -0.810262 | -0.254218 | 0.799327 | |
| 888 | 20 | -0.326419 | -0.144011 | 0.885492 | |
| 889 | 30 | -0.545045 | -0.169935 | 0.865061 | |
| 890 | 10 | 3.504471 | 0.887351 | 0.374890 | |
| 891 | 30 | 12.302034 | 3.437893 | 0.000586 | HH |
| 892 | 20 | 0.943259 | 0.318909 | 0.749795 | |
| 893 | 20 | 0.943259 | 0.318909 | 0.749795 | |
| 894 | 10 | 0.433355 | 0.434498 | 0.663927 | |
| 895 | 0 | 9.044005 | 3.031062 | 0.002437 | LL |
| 896 | 10 | 1.161885 | 0.584057 | 0.559182 | |
| 897 | 10 | 5.909973 | 1.795077 | 0.072641 | |
| 898 | 10 | 5.909973 | 1.795077 | 0.072641 | |
| 899 | 10 | -2.838857 | -1.419934 | 0.155627 | |
| 900 | 20 | -0.094120 | -0.065147 | 0.948057 | |
| 901 | 20 | 0.038060 | 0.019369 | 0.984547 | |
| 902 | 30 | 1.584098 | 0.603482 | 0.546188 | |
| 907 | 10 | -1.419428 | -1.003009 | 0.315857 | |
| 909 | 10 | -1.695788 | -0.847365 | 0.396792 | |
| 910 | 30 | 0.678899 | 0.394255 | 0.693393 | |
| 911 | 10 | 0.156996 | 0.092545 | 0.926265 | |
| 912 | 10 | 0.295175 | 0.210340 | 0.833402 | |
| 915 | 10 | 0.866710 | 0.614790 | 0.538694 | |
| 917 | 10 | -1.419428 | -1.003009 | 0.315857 | |
| 918 | 10 | 0.433355 | 0.434498 | 0.663927 | |
| 919 | 10 | -3.548571 | -1.588356 | 0.112206 | |
| 920 | 10 | -3.391575 | -1.200840 | 0.229813 | |
| 921 | 10 | 3.403923 | 0.694757 | 0.487208 | |
| 922 | 10 | 6.060501 | 0.952042 | 0.341076 | |
| 923 | 10 | 4.685172 | 0.973254 | 0.330427 | |
| 924 | 30 | -5.225114 | -1.657201 | 0.097479 | |
| 925 | 10 | 5.080895 | 1.247190 | 0.212328 | |
| 927 | 10 | 0.433355 | 0.434498 | 0.663927 | |
| 929 | 10 | -0.709714 | -0.708869 | 0.478406 | |
| 930 | 10 | -1.419428 | -1.003009 | 0.315857 | |
| 933 | 0 | -1.645728 | -1.645126 | 0.099944 | |
| 934 | 10 | 0.433355 | 0.434498 | 0.663927 | |
| 937 | 10 | -1.419428 | -1.003009 | 0.315857 | |

| | | | | | |
|---|---|---|---|---|---|
| 938 | 0 | -0.320419 | -0.319473 | 0.749368 | |
| 941 | 10 | 0.728530 | 0.422947 | 0.672334 | |
| 944 | 10 | 0.156996 | 0.092545 | 0.926265 | |
| 946 | 10 | 1.438244 | 1.019239 | 0.308089 | |
| 947 | 10 | 1.752236 | 0.624841 | 0.532075 | |
| 950 | 10 | 11.920494 | 3.331385 | 0.000864 | LL |
| 951 | 10 | -2.129143 | -1.229064 | 0.219048 | |
| 952 | 10 | -3.548571 | -1.588356 | 0.112206 | |
| 953 | 10 | -2.838857 | -1.419934 | 0.155627 | |
| 954 | 20 | 2.351116 | 0.686355 | 0.492489 | |
| 955 | 10 | -8.654750 | -2.412267 | 0.015854 | LH |
| 957 | 10 | 1.004889 | 1.006181 | 0.314328 | |
| 958 | 10 | -0.276359 | -0.194110 | 0.846090 | |
| 960 | 10 | 0.175812 | 0.069408 | 0.944665 | |
| 962 | 10 | 2.443134 | 1.414153 | 0.157317 | |
| 968 | 10 | 0.866710 | 0.614790 | 0.538694 | |
| 969 | 10 | 3.448023 | 1.729196 | 0.083774 | |
| 970 | 20 | 0.434600 | 0.120849 | 0.903811 | |
| 971 | 10 | 1.004889 | 1.006181 | 0.314328 | |
