# Supplementary material for: Impact of environmental variables on Dubas bug infestation rate: A case study from the Sultanate of Oman
Source: PLoS One. 2017 May 30;12(5):e0178109. doi: 10.1371/journal.pone.0178109 (PMC5448759; doi:10.1371/journal.pone.0178109)
Supplement: S3 File — (PDF) [file pone.0178109.s003.pdf]

ID	GiZScore	GiPValue	Gi_Bin	Lat_Y	Long_X	Region	DEM
1	-0.6688	0.5036	0	22.86826	57.75036	IZKI	488
2	0.2503	0.8023	0	22.91831	57.76747	IZKI	528
3	0.3686	0.7124	0	22.93436	57.78356	IZKI	559
4	0.3680	0.7128	0	23.10534	57.29136	AL HAMRA	655
5	1.1694	0.2422	0	23.44414	58.12739	BID BID	196
6	0.2243	0.8225	0	23.03436	57.13364	BAHLA	582
7	0.1260	0.8997	0	22.99806	57.77762	IZKI	608
8	0.6004	0.5482	0	23.06587	57.26240	AL HAMRA	631
9	2.6308	0.0085	3	23.39373	58.14393	BID BID	243
10	2.9007	0.0037	3	23.37734	58.15710	BID BID	266
11	-6.9630	0.0000	-3	25.66752	56.26197	DABA	13
12	0.3760	0.7069	0	23.09629	57.30123	AL HAMRA	659
13	-0.2185	0.8271	0	22.79913	57.65398	MANAH	430
14	0.9023	0.3669	0	23.18367	57.63386	AL RUSTAQ	914
15	-1.3425	0.1794	0	22.82616	57.54900	MANAH	442
16	2.0673	0.0387	2	23.18699	57.61801	AL AWABI	814
17	0.9248	0.3551	0	23.15150	57.85097	SAMIL	586
18	0.8229	0.4105	0	23.15011	57.85025	SAMIL	616
19	3.3636	0.0008	3	23.23188	58.07483	SAMIL	424
20	1.4943	0.1351	0	23.22192	57.55275	AL AWABI	657
21	2.4645	0.0137	2	23.30828	57.52727	AL RUSTAQ	500
22	-0.5267	0.5984	0	22.74464	57.62736	MANAH	404
23	-0.4269	0.6694	0	22.71519	57.55794	MANAH	397
24	-2.1150	0.0344	-2	23.88434	56.41709	YANQAL	699
25	-1.8836	0.0596	-1	23.87365	56.40970	YANQAL	713
26	-0.7805	0.4351	0	22.78910	57.58478	MANAH	428
27	-1.8554	0.0635	-1	23.86103	56.37529	YANQAL	806
28	-1.0154	0.3099	0	22.94384	57.53168	NIZWA	535
29	2.0690	0.0385	2	23.30126	57.98446	SAMIL	381
30	2.4546	0.0141	2	23.31695	58.01809	SAMIL	365
31	2.2061	0.0274	2	22.85302	57.96226	IZKI	520
32	-0.0089	0.9929	0	22.98990	57.67315	NIZWA	684
33	3.2735	0.0011	3	23.31431	58.07358	SAMIL	308
34	1.8177	0.0691	1	23.28880	57.92186	SAMIL	432
35	2.9250	0.0034	3	23.28436	58.04045	SAMIL	362
36	3.0450	0.0023	3	23.29807	58.05973	SAMIL	316
37	0.8227	0.4107	0	23.41274	57.11821	AL RUSTAQ	652
38	1.0478	0.2947	0	23.42275	57.15238	AL RUSTAQ	591
39	-2.1436	0.0321	-2	23.93233	56.34522	YANQAL	907
40	-0.0411	0.9672	0	22.93336	57.75117	IZKI	581
41	0.0655	0.9478	0	22.93386	57.76711	IZKI	542
42	0.2503	0.8023	0	22.91828	57.76686	IZKI	529
43	3.2452	0.0012	3	23.22750	58.05189	SAMIL	461
44	0.3543	0.7231	0	23.06975	57.12775	BAHLA	599
45	0.3037	0.7613	0	23.09935	57.31183	AL HAMRA	669
46	0.1406	0.8882	0	22.93483	57.76764	IZKI	547
47	0.3069	0.7589	0	22.98450	57.78406	IZKI	584
48	-1.1412	0.2538	0	22.94912	57.53598	NIZWA	538
49	-1.1081	0.2678	0	22.95762	57.54336	NIZWA	544
50	0.6852	0.4932	0	23.13933	57.31240	AL HAMRA	913
51	0.9991	0.3177	0	23.15153	57.20078	AL HAMRA	750
52	-2.0580	0.0396	-2	23.83814	56.36473	YANQAL	800
53	2.2105	0.0271	2	23.35061	58.01719	SAMIL	367
54	2.2105	0.0271	2	23.35364	58.02094	SAMIL	366
55	4.9755	0.0000	3	23.08861	58.34249	BID BID	676
56	4.9755	0.0000	3	23.09860	58.35201	BID BID	655
57	-1.3128	0.1893	0	22.85540	57.46589	NIZWA	523
58	-2.7606	0.0058	-3	24.03167	56.17116	AL BURAYMI	629
59	6.3439	0.0000	3	23.17324	58.81460	QURAYYAT	182
60	6.5858	0.0000	3	23.13450	58.81778	QURAYYAT	545
61	-1.3128	0.1893	0	22.85090	57.46690	NIZWA	517

62	5.8953	0.0000	3	23.20562	58.97456	QURAYYAT	18
63	2.4538	0.0141	2	23.35679	58.03692	SAMIL	343
64	0.3601	0.7188	0	23.05409	57.45808	NIZWA	630
65	-0.0429	0.9658	0	23.02132	57.53471	NIZWA	600
66	4.0132	0.0001	3	23.31825	58.21781	BID BID	349
67	1.7118	0.0869	1	23.16786	57.90092	SAMIL	543
68	1.4676	0.1422	0	23.01722	57.98431	SAMIL	731
69	2.3267	0.0200	2	23.00163	58.01743	SAMIL	640
70	2.4308	0.0151	2	23.01781	58.01670	SAMIL	672
71	1.4282	0.1532	0	23.01794	57.96817	SAMIL	804
72	1.7845	0.0743	1	22.96455	57.91210	IZKI	648
73	1.5231	0.1277	0	22.95111	57.91814	IZKI	616
74	1.4907	0.1360	0	22.94952	57.89637	IZKI	640
75	2.1185	0.0341	2	22.89239	57.92952	IZKI	558
76	-2.6485	0.0081	-3	24.30025	56.11761	MAHADAH	623
77	-2.6485	0.0081	-3	24.30106	56.11739	MAHADAH	629
78	-0.6529	0.5138	0	22.79370	57.60293	MANAH	430
79	0.0993	0.9209	0	23.08230	56.81948	IBRI	488
80	-2.6485	0.0081	-3	24.30164	56.11728	MAHADAH	628
81	-1.5866	0.1126	0	24.36683	56.03478	MAHADAH	504
82	-0.9689	0.3326	0	22.93433	57.52365	NIZWA	530
83	0.5939	0.5526	0	23.16862	56.90863	IBRI	580
84	-0.7527	0.4516	0	22.90028	57.53344	NIZWA	510
85	0.6653	0.5059	0	23.21078	56.96160	IBRI	647
86	0.2874	0.7738	0	23.21538	57.01128	IBRI	688
87	0.6229	0.5334	0	23.08497	57.85108	SAMIL	663
88	-0.2341	0.8149	0	23.10083	57.81750	SAMIL	689
89	1.7775	0.0755	1	23.37204	57.97347	SAMIL	427
90	1.6205	0.1051	0	23.32584	57.95021	SAMIL	433
91	0.6479	0.5170	0	23.11893	57.28070	AL HAMRA	660
92	-3.0026	0.0027	-3	24.19927	56.35822	AL BURAYMI	522
93	0.8229	0.4105	0	23.15142	57.85011	SAMIL	555
94	-1.4759	0.1400	0	23.93425	56.42017	YANQAL	630
95	-2.1436	0.0321	-2	23.93678	56.35055	YANQAL	874
96	3.4752	0.0005	3	23.36156	58.10579	BID BID	272
97	2.7948	0.0052	3	23.41022	58.10162	BID BID	243
98	3.7977	0.0001	3	23.33438	58.12394	BID BID	288
99	-0.2432	0.8078	0	23.39994	57.77345	IZKI	293
100	0.5204	0.6028	0	23.23055	57.04418	IBRI	715
101	-2.2054	0.0274	-2	23.85879	56.40494	YANQAL	736
102	4.0144	0.0001	3	23.20872	58.08279	BID BID	463
103	0.1093	0.9130	0	23.21350	57.02454	IBRI	692
104	4.0144	0.0001	3	23.20983	58.08254	BID BID	464
105	0.4405	0.6596	0	23.25285	57.04491	IBRI	727
106	3.2927	0.0010	3	23.23442	58.06825	BID BID	542
107	4.1634	0.0000	3	23.25126	58.10387	BID BID	381
108	4.3694	0.0000	3	23.22160	58.13254	BID BID	446
109	0.4885	0.6252	0	23.13874	56.89599	IBRI	551
110	-7.6658	0.0000	-3	25.63267	56.25608	MADHA	10
111	-8.7396	0.0000	-3	25.27300	56.31971	MADHA	99
112	-8.7396	0.0000	-3	25.28550	56.31058	MADHA	112
113	-8.7396	0.0000	-3	25.27577	56.25364	MADHA	330
114	0.1043	0.9170	0	23.10676	57.07033	IBRI	919
115	-0.5503	0.5821	0	22.78383	57.80100	IZKI	439
116	2.6646	0.0077	3	22.88423	58.01392	IZKI	532
117	-1.0298	0.3031	0	26.06731	56.10064	BUKHA	271
118	-0.7048	0.4809	0	26.10875	56.13055	BUKHA	16
119	-0.7048	0.4809	0	26.11087	56.13079	BUKHA	15
120	3.5261	0.0004	3	26.21691	56.18775	BUKHA	11
121	2.2934	0.0218	2	26.15904	56.17297	BUKHA	13
122	1.0730	0.2832	0	26.13988	56.15829	BUKHA	8
123	1.7615	0.0782	1	23.43614	58.10599	BID BID	215

124	1.7110	0.0871	1	23.43492	58.11127	BID BID	209
125	2.5440	0.0110	2	23.42508	58.09668	BID BID	232
126	-7.9224	0.0000	-3	25.62110	56.24076	DABA	14
127	-7.9091	0.0000	-3	25.60933	56.24330	DABA	18
128	-3.2275	0.0012	-3	24.20608	56.23166	AL BURAYMI	508
129	-3.1814	0.0015	-3	23.61703	55.96778	AS SUNAYNAH	267
130	-3.3973	0.0007	-3	23.59090	55.96081	AS SUNAYNAH	260
131	-1.6915	0.0907	-1	24.46428	56.06720	MAHADAH	616
132	-1.8079	0.0706	-1	24.49859	55.97164	MAHADAH	465
133	-0.0201	0.9840	0	23.05992	57.00410	IBRI	658
134	-2.7606	0.0058	-3	24.03723	56.16043	AL BURAYMI	608
135	-2.9913	0.0028	-3	24.03287	56.21308	AL BURAYMI	750
136	0.1708	0.8644	0	23.07136	56.96689	IBRI	587
137	0.7765	0.4374	0	23.47356	58.10150	BID BID	167
138	2.7796	0.0054	3	23.41076	58.09237	BID BID	253
139	3.1560	0.0016	3	26.19172	56.25272	KHASAB	10
140	3.1560	0.0016	3	26.18887	56.24468	KHASAB	13
141	3.1560	0.0016	3	26.19016	56.24851	KHASAB	20
142	3.1560	0.0016	3	26.18815	56.22248	KHASAB	25
143	3.1560	0.0016	3	26.19499	56.24585	KHASAB	11
144	0.5873	0.5570	0	23.13294	57.08079	IBRI	950
145	-1.6915	0.0907	-1	24.45629	56.05706	MAHADAH	585
146	0.7373	0.4610	0	23.14765	57.08786	IBRI	958
147	2.1049	0.0353	2	23.41733	58.13492	BID BID	205
148	0.4726	0.6365	0	23.18364	57.40164	AL RUSTAQ	1220
149	-2.4015	0.0163	-2	24.75111	56.16753	MAHADAH	352
150	-2.3643	0.0181	-2	24.71655	56.18271	MAHADAH	370
151	-2.3643	0.0181	-2	24.71322	56.16569	MAHADAH	430
152	-2.3531	0.0186	-2	24.69254	56.16548	MAHADAH	438
153	-2.0243	0.0429	-2	24.61444	56.06327	MAHADAH	585
154	0.4854	0.6274	0	23.36437	57.30877	AL RUSTAQ	508
155	0.9822	0.3260	0	23.29050	57.32959	AL RUSTAQ	650
156	1.1848	0.2361	0	23.24864	57.31768	AL RUSTAQ	760
157	1.5169	0.1293	0	23.25723	57.33705	AL RUSTAQ	711
158	1.6144	0.1064	0	23.22164	57.31769	AL RUSTAQ	891
159	1.6664	0.0956	1	23.22289	57.33276	AL RUSTAQ	812
160	0.4749	0.6348	0	23.38781	57.31592	AL RUSTAQ	452
161	-8.7396	0.0000	-3	25.26773	56.29384	MADHA	182
162	-2.6808	0.0073	-3	24.13659	56.21451	AL BURAYMI	663
163	3.1193	0.0018	3	23.43320	58.55417	QURAYYAT	101
164	2.6395	0.0083	3	23.50122	58.66697	AS SEEB	93
165	5.8418	0.0000	3	22.71734	58.51177	IBRA	467
166	0.5002	0.6169	0	23.45810	57.08600	AL RUSTAQ	695
167	-0.0232	0.9815	0	23.39152	57.83953	NAKHAL	326
168	-0.9775	0.3283	0	23.15662	57.74036	NAKHAL	1131
169	-0.7389	0.4599	0	23.45453	57.81029	VADI AL MAAWI	234
170	1.2311	0.2183	0	23.21791	57.44244	AL RUSTAQ	688
171	4.7460	0.0000	3	23.15601	58.31250	BID BID	748
172	4.6861	0.0000	3	23.18125	58.31877	\MA WAAT TAIY	791
173	4.9783	0.0000	3	23.11566	58.47609	\MA WAAT TAIY	485
174	5.1195	0.0000	3	23.12349	58.46162	\MA WAAT TAIY	499
175	4.7335	0.0000	3	23.08581	58.60153	\MA WAAT TAIY	699
176	5.4940	0.0000	3	23.13749	58.40092	\MA WAAT TAIY	579
177	4.7775	0.0000	3	23.08417	58.56733	\MA WAAT TAIY	395
178	4.3385	0.0000	3	23.11667	58.58419	\MA WAAT TAIY	646
179	4.6605	0.0000	3	23.11756	58.54179	\MA WAAT TAIY	434
180	4.6977	0.0000	3	23.11697	58.53433	\MA WAAT TAIY	424
181	4.5181	0.0000	3	23.10206	58.56920	\MA WAAT TAIY	410
182	4.9887	0.0000	3	23.11393	58.51843	IBRA	449
183	5.3967	0.0000	3	22.96822	58.39167	IBRA	925
184	5.9573	0.0000	3	22.96722	58.55075	\MA WAAT TAIY	533
185	7.2604	0.0000	3	22.94658	58.83052	\MA WAAT TAIY	393

186	7.4843	0.0000	3	22.91493	58.88255	AMA WAAT TAIY	461
187	5.0278	0.0000	3	23.04545	58.51468	AMA WAAT TAIY	503
188	6.0120	0.0000	3	23.04317	58.68676	AMA WAAT TAIY	300
189	6.0971	0.0000	3	23.04614	58.66244	AMA WAAT TAIY	313
190	6.1809	0.0000	3	23.04811	58.64263	AMA WAAT TAIY	327
191	7.1603	0.0000	3	22.98144	58.79211	AMA WAAT TAIY	325
192	6.9989	0.0000	3	22.99159	58.78068	AMA WAAT TAIY	323
193	6.4147	0.0000	3	23.02034	58.74039	AMA WAAT TAIY	269
194	4.9755	0.0000	3	23.10890	58.35611	BID BID	647
195	2.6268	0.0086	3	22.89784	58.02599	AL MUDAYBI	536
196	2.9008	0.0037	3	22.92321	58.05579	AL MUDAYBI	561
197	3.5428	0.0004	3	22.93237	58.07752	AL MUDAYBI	578
198	4.6262	0.0000	3	22.98375	58.28478	AL MUDAYBI	770
199	4.8245	0.0000	3	22.96719	58.28486	AL MUDAYBI	825
200	4.5967	0.0000	3	22.88383	58.21769	AL MUDAYBI	615
201	2.2356	0.0254	2	23.31248	58.01047	SAMIL	360
202	2.2105	0.0271	2	23.31030	58.00111	SAMIL	373
203	2.5999	0.0093	3	23.30944	58.02548	SAMIL	341
204	0.5528	0.5804	0	23.35602	57.63840	NAKHAL	366
205	1.4241	0.1544	0	23.18781	57.57332	AL AWABI	749
206	1.4943	0.1351	0	23.18781	57.56552	AL AWABI	735
207	1.0066	0.3141	0	23.42015	57.12853	AL RUSTAQ	613
208	0.8227	0.4107	0	23.41167	57.11678	AL RUSTAQ	654
209	0.8979	0.3693	0	23.41574	57.12146	AL RUSTAQ	629
210	1.0066	0.3141	0	23.42066	57.13149	AL RUSTAQ	613
211	0.9532	0.3405	0	23.42364	57.16529	AL RUSTAQ	586
212	0.6111	0.5411	0	23.40847	57.17187	AL RUSTAQ	574
213	0.2875	0.7738	0	23.42721	57.29790	AL RUSTAQ	445
214	0.3721	0.7098	0	23.05079	57.12802	BAHLA	586
215	0.2068	0.8362	0	23.04141	57.13174	BAHLA	594
216	-0.3103	0.7563	0	22.98820	57.16192	BAHLA	577
217	-2.5150	0.0119	-2	24.65334	56.11267	MAHADAH	558
218	2.6174	0.0089	3	22.64668	59.26968	SUR	498
219	2.7622	0.0057	3	22.64718	59.24756	SUR	593
220	3.0352	0.0024	3	22.65713	59.22029	SUR	842
221	3.9946	0.0001	3	22.42884	58.79885	BIDIYAH	300
222	2.0837	0.0372	2	23.48049	58.32814	BAWSHAR	141
223	6.7128	0.0000	3	23.06001	58.91080	QURAYYAT	228
224	7.1873	0.0000	3	23.04924	58.92720	QURAYYAT	249
225	7.0321	0.0000	3	23.04062	58.93493	QURAYYAT	280
226	7.2386	0.0000	3	22.88552	58.76998	AL QABIL	583
227	0.3973	0.6912	0	23.22870	57.06254	IBRI	732
228	0.4732	0.6361	0	23.23356	57.05028	IBRI	784
229	0.8499	0.3954	0	23.28446	56.89397	IBRI	668
230	0.8357	0.4033	0	23.28232	56.92149	IBRI	694
231	0.6727	0.5012	0	23.40228	56.99304	IBRI	938
232	-0.7696	0.4415	0	23.56822	56.63472	YANQAL	647
233	-1.2369	0.2161	0	23.63492	56.63486	YANQAL	703
234	1.0118	0.3117	0	23.38962	56.92150	IBRI	806
235	0.7866	0.4315	0	23.40856	56.88854	IBRI	789
236	0.7232	0.4696	0	23.37291	56.88266	IBRI	735
237	1.0253	0.3052	0	23.42600	56.82932	IBRI	644
238	1.2762	0.2019	0	23.55025	56.78358	IBRI	710
239	0.8354	0.4035	0	23.63552	56.80622	IBRI	737
240	-0.1148	0.9086	0	23.68888	56.75441	SAHAM	712
241	-0.2885	0.7729	0	23.70482	56.74983	SAHAM	676
242	-1.4492	0.1473	0	23.50108	56.48422	DANK	589
243	-2.6326	0.0085	-3	24.08942	56.11686	AL BURAYMI	568
244	-2.6241	0.0087	-3	24.12876	56.20106	AL BURAYMI	694
245	-2.6410	0.0083	-3	24.09261	56.25476	AL BURAYMI	826
246	-3.3656	0.0008	-3	24.19535	56.26367	AL BURAYMI	528
247	0.2786	0.7806	0	23.09029	57.29232	AL HAMRA	645

248	1.4027	0.1607	0	23.43392	58.13369	BID BID	188
249	1.2474	0.2123	0	23.43736	58.13417	BID BID	196
250	-2.4719	0.0134	-2	22.38378	57.51814	ADAM	287
251	-0.2035	0.8387	0	23.00615	57.77905	IZKI	605
252	2.2435	0.0249	2	23.41749	58.14856	BID BID	219
253	2.8890	0.0039	3	23.38002	58.15081	BID BID	259
254	-1.0298	0.3031	0	25.94139	56.41913	DABA	12
255	0.5754	0.5650	0	23.11092	57.28838	AL HAMRA	656
256	-0.8120	0.4168	0	22.79513	57.58555	MANAH	423
257	-0.9689	0.3326	0	22.93693	57.52688	NIZWA	524
258	1.9172	0.0552	1	23.18209	57.60835	AL AWABI	860
259	3.8369	0.0001	3	23.22803	58.07989	SAMIL	422
260	1.5645	0.1177	0	23.21634	57.55516	AL AWABI	685
261	2.2385	0.0252	2	23.31102	57.53345	AL RUSTAQ	495
262	-3.1076	0.0019	-3	23.61703	55.95033	AS SUNAYNAH	260
263	-1.9718	0.0486	-2	23.88379	56.41472	YANQAL	703
264	-2.2054	0.0274	-2	23.85904	56.40737	YANQAL	729
265	-1.8554	0.0635	-1	23.86817	56.36772	YANQAL	831
266	-0.9689	0.3326	0	22.92748	57.52751	NIZWA	513
267	-0.0985	0.9216	0	22.98969	57.67053	NIZWA	693
268	3.3451	0.0008	3	23.31881	58.08175	SAMIL	300
269	2.0755	0.0379	2	23.29008	57.97486	SAMIL	393
270	2.7809	0.0054	3	23.27793	58.03367	SAMIL	375
271	2.7087	0.0068	3	23.30452	58.03365	SAMIL	336
272	0.9314	0.3516	0	23.42431	57.12356	AL RUSTAQ	620
273	1.0478	0.2947	0	23.42239	57.15113	AL RUSTAQ	595
274	-2.3424	0.0192	-2	23.94280	56.35012	YANQAL	851
275	0.5195	0.6034	0	22.09864	59.27752	KAMIL WA AL W	118
276	0.3543	0.7231	0	23.07188	57.12657	BAHLA	607
277	0.5754	0.5650	0	23.10809	57.28988	AL HAMRA	651
278	-1.0885	0.2764	0	22.96536	57.55231	NIZWA	542
279	-1.0484	0.2945	0	22.93472	57.53414	NIZWA	514
280	0.8428	0.3993	0	23.15011	57.31714	AL HAMRA	1052
281	-2.2054	0.0274	-2	23.85492	56.40554	YANQAL	731
282	2.2105	0.0271	2	23.35233	58.01940	SAMIL	364
283	5.0657	0.0000	3	23.11828	58.31900	BID BID	689
284	4.9755	0.0000	3	23.10686	58.35470	BID BID	656
285	-0.7527	0.4516	0	22.86318	57.52693	NIZWA	469
286	-1.3362	0.1815	0	22.80125	57.60078	MANAH	424
287	-2.9261	0.0034	-3	24.04630	56.17622	AL BURAYMI	624
288	6.5879	0.0000	3	23.14806	58.83122	QURAYYAT	182
289	6.0490	0.0000	3	23.17384	58.92145	QURAYYAT	53
290	2.6240	0.0087	3	23.35707	58.04815	SAMIL	323
291	0.2869	0.7742	0	23.05447	57.46037	NIZWA	632
292	-0.0118	0.9906	0	23.01979	57.53703	NIZWA	590
293	4.0884	0.0000	3	23.30097	58.21767	BID BID	368
294	1.3869	0.1655	0	22.97644	57.91733	IZKI	663
295	1.1919	0.2333	0	22.95047	57.88494	IZKI	658
296	0.2676	0.7890	0	23.09118	56.82719	IBRI	487
297	-0.9826	0.3258	0	22.95039	57.53138	NIZWA	547
298	0.5939	0.5526	0	23.17059	56.91219	IBRI	582
299	0.6653	0.5059	0	23.20128	56.95150	IBRI	680
300	0.2874	0.7738	0	23.21780	57.01728	IBRI	735
301	1.1962	0.2316	0	23.21686	57.86733	SAMIL	751
302	1.9874	0.0469	2	23.36728	57.96747	SAMIL	444
303	1.6466	0.0996	1	23.32546	57.95233	SAMIL	428
304	0.6479	0.5170	0	23.11507	57.28442	AL HAMRA	661
305	-2.7231	0.0065	-3	24.08474	56.01606	AL BURAYMI	442
306	-1.8836	0.0596	-1	23.99014	56.48299	YANQAL	457
307	-2.1436	0.0321	-2	23.93335	56.34841	YANQAL	897
308	3.1489	0.0016	3	23.38176	58.10000	BID BID	247
309	3.4752	0.0005	3	23.36763	58.10781	BID BID	264

310	3.7977	0.0001	3	23.33176	58.12296	BID BID	297
311	0.1237	0.9016	0	23.38215	57.82488	IZKI	329
312	-0.2679	0.7888	0	22.78353	57.85019	IZKI	472
313	0.2874	0.7738	0	23.21419	57.01444	IBRI	685
314	-2.0870	0.0369	-2	23.84665	56.41430	YANQAL	748
315	3.3869	0.0007	3	23.22849	58.06808	BID BID	443
316	3.9073	0.0001	3	23.21761	58.07747	BID BID	452
317	0.3647	0.7153	0	23.24934	57.04562	IBRI	722
318	4.1634	0.0000	3	23.24989	58.10216	BID BID	382
319	4.3587	0.0000	3	23.22153	58.13640	BID BID	450
320	0.4885	0.6252	0	23.13435	56.89536	IBRI	550
321	-7.6658	0.0000	-3	25.63353	56.26814	MADHA	9
322	-8.7396	0.0000	-3	25.27746	56.31852	MADHA	100
323	-8.7396	0.0000	-3	25.28119	56.31514	MADHA	108
324	-8.7396	0.0000	-3	25.28196	56.32579	MADHA	75
325	0.1215	0.9033	0	23.10255	57.05098	IBRI	1260
326	1.9729	0.0485	2	26.14693	56.16333	BUKHA	15
327	0.8164	0.4143	0	26.13497	56.15142	BUKHA	9
328	1.8007	0.0718	1	23.43000	58.11766	BID BID	198
329	1.6342	0.1022	0	23.44182	58.09593	BID BID	232
330	1.6342	0.1022	0	23.44269	58.09721	BID BID	240
331	-7.9224	0.0000	-3	25.61932	56.25418	DABA	10
332	-8.4531	0.0000	-3	25.60476	56.25058	DABA	20
333	-1.6880	0.0914	-1	24.22177	55.95981	AL BURAYMI	406
334	-3.0429	0.0023	-3	23.61332	55.94639	AS SUNAYNAH	262
335	-0.2249	0.8221	0	23.05718	56.99232	IBRI	625
336	-2.6485	0.0081	-3	24.03473	56.15101	AL BURAYMI	584
337	-3.0457	0.0023	-3	24.03210	56.21660	AL BURAYMI	748
338	0.0896	0.9286	0	23.06434	56.96951	IBRI	584
339	0.9343	0.3501	0	23.46414	58.10440	BID BID	168
340	2.7052	0.0068	3	23.40749	58.12479	BID BID	216
341	3.1560	0.0016	3	26.18526	56.25658	KHASAB	14
342	3.1560	0.0016	3	26.18766	56.25073	KHASAB	14
343	0.6623	0.5078	0	23.13290	57.08960	IBRI	941
344	-1.6915	0.0907	-1	24.45006	56.05708	MAHADAH	576
345	0.6623	0.5078	0	23.14001	57.08697	IBRI	947
346	-1.8501	0.0643	-1	24.36731	56.08361	MAHADAH	688
347	2.1049	0.0353	2	23.41689	58.13378	BID BID	203
348	-2.4759	0.0133	-2	24.71885	56.18488	MAHADAH	358
349	-2.2068	0.0273	-2	24.70287	56.18234	MAHADAH	406
350	-2.3531	0.0186	-2	24.69236	56.16988	MAHADAH	421
351	0.4854	0.6274	0	23.36312	57.31027	AL RUSTAQ	522
352	0.9822	0.3260	0	23.28843	57.32987	AL RUSTAQ	658
353	1.5952	0.1107	0	23.22268	57.32048	AL RUSTAQ	852
354	1.6213	0.1050	0	23.22135	57.33478	AL RUSTAQ	809
355	0.4433	0.6575	0	23.38540	57.30952	AL RUSTAQ	466
356	-8.7396	0.0000	-3	25.28378	56.26013	MADHA	321
357	-2.5599	0.0105	-2	24.13391	56.21689	AL BURAYMI	668
358	5.8440	0.0000	3	23.26978	58.74817	QURAYYAT	175
359	0.6976	0.4854	0	23.49253	57.00643	AL RUSTAQ	778
360	0.0832	0.9337	0	23.39631	57.84164	NAKHAL	313
361	-0.5256	0.5992	0	23.44684	57.80927	VADI AL MAAWI	251
362	1.3043	0.1921	0	23.21739	57.40125	AL RUSTAQ	836
363	1.1289	0.2589	0	23.21648	57.44249	AL RUSTAQ	688
364	4.6366	0.0000	3	23.15292	58.31434	BID BID	731
365	4.9452	0.0000	3	23.18381	58.35042	AMA WAAT TAIY	1093
366	5.0858	0.0000	3	23.13372	58.38478	AMA WAAT TAIY	597
367	5.1195	0.0000	3	23.10216	58.45474	AMA WAAT TAIY	556
368	5.4101	0.0000	3	23.14312	58.42390	AMA WAAT TAIY	553
369	5.3371	0.0000	3	23.13992	58.41463	AMA WAAT TAIY	562
370	4.6795	0.0000	3	23.10657	58.56035	AMA WAAT TAIY	415
371	5.1802	0.0000	3	23.11505	58.50924	AMA WAAT TAIY	459

372	4.8010	0.0000	3	23.09787	58.57038	IBRA	410
373	5.6395	0.0000	3	22.96803	58.51758	\MA WAAT TAIY	659
374	6.2320	0.0000	3	22.98400	58.56692	\MA WAAT TAIY	550
375	7.2604	0.0000	3	22.94586	58.82686	\MA WAAT TAIY	385
376	7.4843	0.0000	3	22.90122	58.87703	\MA WAAT TAIY	475
377	5.4859	0.0000	3	23.06711	58.60156	\MA WAAT TAIY	354
378	4.8408	0.0000	3	23.07486	58.57022	\MA WAAT TAIY	390
379	6.1716	0.0000	3	23.04546	58.69019	\MA WAAT TAIY	298
380	6.1873	0.0000	3	23.05285	58.61690	\MA WAAT TAIY	342
381	6.1873	0.0000	3	23.04711	58.62536	\MA WAAT TAIY	332
382	6.9989	0.0000	3	22.99153	58.78925	\MA WAAT TAIY	320
383	7.0816	0.0000	3	22.98513	58.79099	\MA WAAT TAIY	325
384	6.4133	0.0000	3	23.01901	58.74410	\MA WAAT TAIY	269
385	4.9856	0.0000	3	23.11758	58.36706	BID BID	676
386	2.8556	0.0043	3	22.90156	58.05008	AL MUDAYBI	564
387	2.9494	0.0032	3	22.91797	58.05103	AL MUDAYBI	546
388	3.2155	0.0013	3	22.91828	58.06797	AL MUDAYBI	593
389	3.0948	0.0020	3	22.91667	58.06753	AL MUDAYBI	584
390	3.3935	0.0007	3	22.84717	58.17332	AL MUDAYBI	583
391	3.2506	0.0012	3	22.82771	58.16549	AL MUDAYBI	578
392	2.5283	0.0115	2	22.80014	58.13367	AL MUDAYBI	535
393	2.7569	0.0058	3	23.31362	58.04188	SAMIL	327
394	1.8746	0.0608	1	23.16683	57.91781	SAMIL	563
395	2.6121	0.0090	3	23.31015	58.03609	SAMIL	332
396	2.9012	0.0037	3	23.30180	58.04254	SAMIL	329
397	0.2981	0.7656	0	23.46711	57.66764	NAKHAL	221
398	0.5528	0.5804	0	23.35409	57.63278	NAKHAL	370
399	1.3711	0.1703	0	23.18602	57.63087	AL AWABI	845
400	-0.8066	0.4199	0	23.20164	57.67869	AL AWABI	773
401	0.9374	0.3485	0	23.44877	57.11461	AL RUSTAQ	659
402	0.8979	0.3693	0	23.41488	57.12035	AL RUSTAQ	640
403	0.8979	0.3693	0	23.41383	57.11961	AL RUSTAQ	633
404	1.1564	0.2475	0	23.42190	57.13973	AL RUSTAQ	603
405	0.3972	0.6912	0	23.37594	57.18677	AL RUSTAQ	565
406	0.5928	0.5533	0	23.40404	57.17705	AL RUSTAQ	559
407	1.0432	0.2968	0	23.42321	57.43244	AL RUSTAQ	310
408	0.2068	0.8362	0	23.04190	57.13512	BAHLA	591
409	-0.3256	0.7448	0	22.99109	57.14095	BAHLA	562
410	-0.3873	0.6985	0	22.98763	57.16010	BAHLA	582
411	-2.5150	0.0119	-2	24.64780	56.11148	MAHADAH	564
412	2.6255	0.0087	3	22.55330	59.32155	SUR	251
413	2.6174	0.0089	3	22.64692	59.26179	SUR	543
414	2.3032	0.0213	2	22.64256	59.28732	SUR	455
415	3.6539	0.0003	3	22.60402	59.08393	'ADI BANI KHALI	637
416	3.1646	0.0016	3	22.56700	59.10139	'ADI BANI KHALI	602
417	3.1664	0.0015	3	22.55033	59.11797	'ADI BANI KHALI	563
418	1.1252	0.2605	0	22.12970	59.17469	_AN BANI BU HA	159
419	3.7625	0.0002	3	22.44769	58.86222	BIDIYAH	291
420	4.0851	0.0000	3	22.44212	58.81708	BIDIYAH	305
421	2.2395	0.0251	2	23.47623	58.32523	BAWSHAR	146
422	4.0175	0.0001	3	23.25017	58.55056	AL AMRAT	470
423	6.0131	0.0000	3	23.23414	58.70114	QURAYYAT	349
424	6.6613	0.0000	3	22.73316	58.68956	AL QABIL	412
425	7.2426	0.0000	3	22.87849	58.75679	AL QABIL	572
426	7.1642	0.0000	3	22.89265	58.74060	AL QABIL	613
427	6.1070	0.0000	3	22.72359	58.65496	AL QABIL	406
428	0.5490	0.5830	0	23.22942	57.06583	IBRI	743
429	0.8991	0.3686	0	23.27864	56.89423	IBRI	647
430	0.6743	0.5001	0	23.27890	56.93120	IBRI	698
431	0.6727	0.5012	0	23.39704	56.98638	IBRI	927
432	0.7318	0.4643	0	23.39468	56.91585	IBRI	810
433	0.6743	0.5001	0	23.40250	56.88818	IBRI	773

434	0.5870	0.5572	0	23.40025	56.86123	IBRI	702
435	1.0166	0.3093	0	23.42112	56.83830	IBRI	641
436	0.5065	0.6125	0	23.65684	56.80217	IBRI	829
437	0.3739	0.7085	0	23.66732	56.77672	SAHAM	801
438	-0.2885	0.7729	0	23.70565	56.74768	SAHAM	693
439	-1.9695	0.0489	-2	24.14938	56.01064	AL BURAYMI	430
440	-2.5848	0.0097	-3	24.09378	56.24591	AL BURAYMI	797
441	-3.5028	0.0005	-3	24.20111	56.26333	AL BURAYMI	519
442	-1.1738	0.2405	0	24.40017	56.03347	MAHADAH	528
443	2.7681	0.0056	3	22.58981	59.28762	SUR	520
444	2.4571	0.0140	2	22.51280	59.33669	SUR	196
445	1.8119	0.0700	1	22.69924	59.37001	SUR	10
446	3.7646	0.0002	3	22.81744	59.25136	SUR	137
447	3.9928	0.0001	3	22.83397	59.23456	SUR	170
448	1.1125	0.2659	0	23.63446	57.12042	SAHAM	309
449	1.2102	0.2262	0	23.65533	57.16111	SAHAM	260
450	0.5331	0.5940	0	23.48631	57.44464	AL RUSTAQ	256
451	1.3190	0.1872	0	23.56409	57.33531	AL RUSTAQ	212
452	1.2197	0.2226	0	23.52408	57.33092	AL RUSTAQ	270
453	1.5605	0.1186	0	23.52001	57.22486	AL RUSTAQ	449
454	1.2801	0.2005	0	23.47782	57.19328	AL RUSTAQ	593
455	1.4037	0.1604	0	23.48690	57.18100	AL RUSTAQ	560
456	0.1670	0.8674	0	23.42767	57.30301	AL RUSTAQ	447
457	-0.2681	0.7886	0	23.55238	57.44561	AL RUSTAQ	202
458	1.0188	0.3083	0	23.50374	57.18852	AL RUSTAQ	512
459	0.8411	0.4003	0	23.54501	57.21767	AL RUSTAQ	399
460	1.3142	0.1888	0	23.31763	57.58597	AL AWABI	427
461	1.3406	0.1801	0	23.31806	57.58278	AL AWABI	433
462	2.6200	0.0088	3	23.43369	56.74022	IBRI	540
463	-1.4897	0.1363	0	23.60128	56.53988	YANQAL	563
464	-1.7455	0.0809	-1	23.69692	56.50884	YANQAL	720
465	-1.5042	0.1325	0	23.68541	56.55744	YANQAL	737
466	-2.7606	0.0058	-3	23.93446	56.19395	DANK	619
467	-2.8713	0.0041	-3	23.92548	56.23199	DANK	698
468	-2.9261	0.0034	-3	23.93626	56.24772	DANK	707
469	3.4891	0.0005	3	23.40058	58.51764	AL AMRAT	141
470	3.1959	0.0014	3	23.43394	58.55164	AL AMRAT	126
471	3.0970	0.0020	3	23.43419	58.56349	AL AMRAT	102
472	5.3481	0.0000	3	22.86681	58.43333	IBRA	588
473	-2.7606	0.0058	-3	24.01825	56.16744	AL BURAYMI	895
474	6.5879	0.0000	3	23.14487	58.82781	QURAYYAT	187
475	-0.1612	0.8719	0	23.62887	58.18431	AS SEEB	29
476	1.3896	0.1646	0	23.58053	58.41979	AS SEEB	13
477	5.8066	0.0000	3	23.19555	58.98598	QURAYYAT	15
478	2.1077	0.0351	2	23.50888	58.61890	QURAYYAT	26
479	3.0449	0.0023	3	23.45016	58.48505	QURAYYAT	158
480	5.8691	0.0000	3	23.21717	58.68461	QURAYYAT	266
481	5.9250	0.0000	3	23.25562	58.78172	QURAYYAT	138
482	6.4528	0.0000	3	23.05549	58.98118	QURAYYAT	78
483	6.5383	0.0000	3	23.04478	58.98089	QURAYYAT	99
484	6.1350	0.0000	3	23.16781	58.91800	QURAYYAT	53
485	-8.7396	0.0000	-3	25.27206	56.31796	MADHA	103
486	-8.7396	0.0000	-3	25.28041	56.31843	MADHA	97
487	-2.6678	0.0076	-3	24.70144	56.29120	SHINAS	263
488	1.3241	0.1855	0	23.83453	57.10047	AL KHABURAH	90
489	1.1862	0.2355	0	23.81728	57.11722	AL KHABURAH	97
490	-2.3017	0.0214	-2	24.62879	56.28347	SHINAS	423
491	1.6756	0.0938	1	23.68141	57.07208	AL KHABURAH	354
492	2.0104	0.0444	2	23.73941	57.04629	AL KHABURAH	285
493	1.8975	0.0578	1	23.72268	57.03709	AL KHABURAH	326
494	1.6858	0.0918	1	23.76979	57.01659	AL KHABURAH	212
495	1.6904	0.0909	1	23.75045	56.97374	AL KHABURAH	310

496	-2.3717	0.0177	-2	23.92013	56.69510	AL KHABURAH	404
497	-2.1160	0.0343	-2	23.83436	56.63100	AL KHABURAH	634
498	-3.2502	0.0012	-3	24.04768	56.78298	SAHAM	105
499	-2.8485	0.0044	-3	23.99937	56.68393	SAHAM	259
500	-2.7524	0.0059	-3	23.98599	56.64807	SAHAM	364
501	-1.7500	0.0801	-1	23.84408	56.78254	AL KHABURAH	314
502	-1.7285	0.0839	-1	23.75378	56.68371	AL KHABURAH	648
503	-2.2908	0.0220	-2	23.88939	56.73353	AL KHABURAH	372
504	-2.7449	0.0061	-3	23.91817	56.71557	AL KHABURAH	360
505	-2.8028	0.0051	-3	23.91934	56.75577	AL KHABURAH	276
506	-2.0710	0.0384	-2	22.65053	57.33453	BAHLA	372
507	-0.2035	0.8387	0	23.00139	57.78381	IZKI	600
508	-1.0458	0.2957	0	22.92572	57.68222	NIZWA	571
509	-0.0429	0.9658	0	23.02179	57.53269	NIZWA	590
510	1.9228	0.0545	1	23.30581	57.99274	SAMIL	378
511	0.0972	0.9225	0	23.11744	57.83416	SAMIL	608
512	0.0972	0.9225	0	23.11699	57.83412	SAMIL	618
513	0.2587	0.7958	0	23.05142	57.45875	NIZWA	634
514	-0.9800	0.3271	0	22.96355	57.54773	NIZWA	536
515	4.1634	0.0000	3	23.25173	58.10255	DID DID	378
516	2.7796	0.0054	3	23.40451	58.12209	DID DID	220
517	3.5811	0.0003	3	23.34406	58.11314	DID DID	270
518	1.4027	0.1607	0	23.43486	58.13411	DID DID	186
519	1.1694	0.2422	0	23.45786	58.09999	DID DID	212
520	2.7291	0.0064	3	23.41140	58.08960	DID DID	255
521	1.7110	0.0871	1	23.45042	58.21758	DID DID	197
522	2.6243	0.0087	3	23.30436	58.02759	SAMIL	349
523	0.8555	0.3923	0	23.46839	58.10207	DID DID	168
524	4.3802	0.0000	3	23.21667	58.14225	DID DID	458
525	3.9073	0.0001	3	23.21658	58.07741	DID DID	450
526	0.8229	0.4105	0	23.15064	57.85086	SAMIL	619
527	1.4676	0.1422	0	23.01821	57.98483	SAMIL	732
528	2.2152	0.0267	2	23.01760	58.00032	SAMIL	777
529	2.3267	0.0200	2	23.00144	58.01737	SAMIL	640
530	3.2104	0.0013	3	23.08417	58.08362	SAMIL	924
531	3.1881	0.0014	3	23.38893	58.10314	DID DID	239
532	-2.7268	0.0064	-3	22.61778	57.31717	BAHLA	356
533	0.6628	0.5075	0	23.10171	57.16085	BAHLA	670
534	0.2156	0.8293	0	23.08310	57.13132	BAHLA	607
535	3.8683	0.0001	3	23.24162	58.23351	DID DID	499
536	3.4752	0.0005	3	23.36513	58.10662	DID DID	267
537	-1.0235	0.3061	0	22.91722	57.25072	BAHLA	515
538	0.4236	0.6718	0	23.08512	57.29732	AL HAMRA	646
539	0.3511	0.7255	0	23.10125	57.29298	AL HAMRA	656
540	5.7058	0.0000	3	22.98342	58.53422	AMA WAATTAIY	542
541	0.3120	0.7550	0	22.55047	58.00031	AL MUDAYBI	366
542	7.4052	0.0000	3	22.90360	58.88208	AMA WAATTAIY	484
543	7.4274	0.0000	3	22.94863	58.82305	AMA WAATTAIY	386
544	2.8692	0.0041	3	22.86096	58.05294	AL MUDAYBI	551
545	2.4706	0.0135	2	22.53735	59.38350	SUR	116
546	2.7588	0.0058	3	22.64524	59.25177	SUR	594
547	6.0894	0.0000	3	22.72722	58.52992	IBRA	463
548	7.1603	0.0000	3	22.97750	58.79777	AMA WAATTAIY	324
549	0.4988	0.6179	0	22.68792	58.02521	AL MUDAYBI	429
550	0.6301	0.5286	0	22.61454	58.04168	AL MUDAYBI	409
551	0.4832	0.6290	0	22.57750	58.01812	AL MUDAYBI	392
552	0.6052	0.5450	0	22.55142	58.01781	AL MUDAYBI	366
553	1.9393	0.0525	1	23.23055	56.50605	IBRI	364
554	-1.4897	0.1363	0	23.60456	56.53596	YNQAL	567
555	2.1625	0.0306	2	23.28367	56.53483	IBRI	390
556	0.2710	0.7864	0	23.07200	56.95766	IBRI	570
557	-0.1580	0.8744	0	23.05935	56.98263	IBRI	612

558	0.2518	0.8012	0	23.11521	56.93560	IBRI	589
559	0.0629	0.9498	0	23.07639	56.84430	IBRI	494
560	0.6653	0.5059	0	23.20904	56.95953	IBRI	633
561	-2.3480	0.0189	-2	24.05293	56.56963	SOHR	283
562	1.7839	0.0744	1	23.29972	57.54608	AL RUSTAQ	494
563	6.1771	0.0000	3	22.75843	58.50357	IBRA	489
564	5.3720	0.0000	3	22.90022	58.41814	IBRA	653
565	5.4254	0.0000	3	22.88442	58.41806	IBRA	807
566	3.1193	0.0018	3	23.43568	58.55828	AL AMRAT	88
567	6.5115	0.0000	3	23.15936	58.82996	QURAYYAT	158
568	5.8066	0.0000	3	23.17523	58.98838	QURAYYAT	12
569	5.8440	0.0000	3	23.27584	58.75329	QURAYYAT	161
570	-3.1209	0.0018	-3	24.01814	56.25069	AL BURAYMI	934
571	-2.4015	0.0163	-2	24.71918	56.18074	MAHADAH	374
572	3.1560	0.0016	3	26.18992	56.25519	KHASAB	15
573	-0.0702	0.9441	0	23.52144	57.48896	AL RUSTAQ	216
574	0.2354	0.8139	0	23.57437	57.44401	AL RUSTAQ	184
575	1.3950	0.1630	0	23.54144	57.33863	AL RUSTAQ	236
576	1.3888	0.1649	0	23.51133	57.22600	AL RUSTAQ	477
577	1.3554	0.1753	0	23.48419	57.18275	AL RUSTAQ	560
578	0.5605	0.5751	0	23.43291	57.32324	AL RUSTAQ	429
579	0.1024	0.9184	0	23.56513	57.44251	AL RUSTAQ	194
580	1.0188	0.3083	0	23.50627	57.18986	AL RUSTAQ	500
581	1.0881	0.2765	0	23.55525	57.22927	AL RUSTAQ	351
582	-2.1802	0.0292	-2	24.38422	56.30136	MAHADAH	555
583	-3.5028	0.0005	-3	24.20176	56.26574	AL BURAYMI	511
584	-1.3765	0.1687	0	24.40047	55.96794	MAHADAH	427
585	-1.4166	0.1566	0	24.36808	56.03450	MAHADAH	503
586	-1.1022	0.2704	0	24.38358	56.03444	MAHADAH	509
587	-1.6915	0.0907	-1	24.43788	56.06090	MAHADAH	603
588	-1.6915	0.0907	-1	24.48383	56.06736	MAHADAH	612
589	-2.0243	0.0429	-2	24.62861	56.07401	MAHADAH	609
590	-2.5463	0.0109	-2	24.68442	56.16717	MAHADAH	445
591	-2.4015	0.0163	-2	24.72126	56.18209	MAHADAH	368
592	-2.6410	0.0083	-3	24.09510	56.25376	AL BURAYMI	818
593	-2.6485	0.0081	-3	24.30139	56.11806	MAHADAH	627
594	-1.9695	0.0489	-2	24.31694	56.11794	MAHADAH	653
595	3.0151	0.0026	3	23.46476	58.58615	QURAYYAT	74
596	2.2923	0.0219	2	23.49127	58.61420	QURAYYAT	40
597	3.4891	0.0005	3	23.40059	58.51764	QURAYYAT	141
598	4.0497	0.0001	3	23.23452	58.55166	QURAYYAT	922
599	6.9577	0.0000	3	23.04507	58.94110	QURAYYAT	212
600	3.0223	0.0025	3	23.46301	58.47894	QURAYYAT	153
601	6.5879	0.0000	3	23.14944	58.83272	QURAYYAT	166
602	6.8465	0.0000	3	23.09078	58.86509	QURAYYAT	116
603	6.5015	0.0000	3	23.08895	58.85741	QURAYYAT	126
604	3.4675	0.0005	3	23.26683	58.58478	QURAYYAT	410
605	5.5132	0.0000	3	23.32189	58.74044	QURAYYAT	199
606	5.9250	0.0000	3	23.25089	58.78389	QURAYYAT	141
607	-2.0950	0.0362	-2	22.54404	57.52211	DABA	311
608	0.2503	0.8023	0	23.06406	57.12735	DABA	591
609	0.6705	0.5025	0	23.19047	57.38795	DABA	977
610	-2.5129	0.0120	-2	23.91744	56.70161	AL KHABURAH	475
611	1.2517	0.2107	0	23.39177	57.41763	AL RUSTAQ	359
612	1.6797	0.0930	1	23.80122	56.98372	AL KHABURAH	218
613	1.4448	0.1485	0	23.67148	56.92316	AL KHABURAH	413
614	1.8414	0.0656	1	23.30233	57.54904	AL RUSTAQ	482
615	2.4645	0.0137	2	23.31026	57.52900	AL RUSTAQ	497
616	-0.5536	0.5798	0	23.18082	57.64961	AL RUSTAQ	880
617	0.9060	0.3649	0	23.46058	57.31124	AL RUSTAQ	394
618	0.9060	0.3649	0	23.46366	57.31110	AL RUSTAQ	395
619	1.3125	0.1894	0	23.54770	57.33815	AL RUSTAQ	240

620	1.4540	0.1460	0	23.55517	57.33689	AL RUSTAQ	205
621	1.2197	0.2226	0	23.52888	57.32960	AL RUSTAQ	263
622	-2.3116	0.0208	-2	24.45020	56.28484	LIWA	515
623	1.2527	0.2103	0	23.57584	57.21259	AL RUSTAQ	331
624	0.6247	0.5322	0	23.45799	57.03696	AL RUSTAQ	847
625	-3.1951	0.0014	-3	24.35103	56.36767	LIWA	325
626	0.7633	0.4453	0	23.49013	57.11234	AL RUSTAQ	662
627	-3.5587	0.0004	-3	24.23003	56.35219	SUR	365
628	-3.0564	0.0022	-3	24.19065	56.31706	SUR	537
629	0.4749	0.6348	0	23.38738	57.31855	AL RUSTAQ	443
630	0.3210	0.7482	0	23.36961	57.30551	AL RUSTAQ	527
631	0.8970	0.3697	0	23.31256	57.31967	AL RUSTAQ	619
632	1.5501	0.1211	0	23.22372	57.32253	AL RUSTAQ	823
633	1.6664	0.0956	1	23.22467	57.33362	AL RUSTAQ	819
634	0.8913	0.3727	0	23.54483	57.21524	AL RUSTAQ	390
635	1.0680	0.2855	0	23.50824	57.19045	AL RUSTAQ	485
636	-0.1726	0.8630	0	23.78442	56.86775	SAHAM	310
637	-2.0285	0.0425	-2	24.00424	56.51147	SUR	397
638	4.3421	0.0000	3	23.21489	58.14018	BID BID	450
639	2.3941	0.0167	2	23.40195	58.16172	BID BID	255
640	0.3145	0.7532	0	23.03852	57.12632	BAHLA	585
641	-0.5575	0.5772	0	22.97857	57.13823	BAHLA	549
642	2.4064	0.0161	2	23.40749	58.14159	BID BID	218
643	2.7440	0.0061	3	23.41095	58.09419	BID BID	254
644	2.5440	0.0110	2	23.42365	58.09555	BID BID	239
645	1.0913	0.2752	0	23.45752	58.10348	BID BID	196
646	1.7242	0.0847	1	23.43232	58.11706	BID BID	193
647	0.9343	0.3501	0	23.46182	58.10154	BID BID	183
648	0.3037	0.7613	0	23.08504	57.32288	AL HAMRA	662
649	1.9532	0.0508	1	23.42076	58.13365	BID BID	207
650	0.4471	0.6548	0	23.06700	57.26764	AL HAMRA	633
651	0.2314	0.8170	0	23.08423	57.32826	AL HAMRA	658
652	2.8291	0.0047	3	23.30681	58.04636	SAMIL	324
653	2.9250	0.0034	3	23.28214	58.03906	SAMIL	365
654	2.7087	0.0068	3	23.30276	58.03262	SAMIL	342
655	-0.0331	0.9736	0	23.10094	57.83379	SAMIL	642
656	0.3936	0.6939	0	23.04205	57.12096	BAHLA	581
657	0.8229	0.4105	0	23.15156	57.85022	SAMIL	555
658	0.7013	0.4831	0	23.18352	57.86690	SAMIL	524
659	1.1962	0.2316	0	23.21703	57.86786	SAMIL	724
660	1.7118	0.0869	1	23.16800	57.90111	SAMIL	543
661	-0.0683	0.9456	0	23.49694	57.83385	IZKI	181
662	2.0690	0.0385	2	23.30590	57.98855	SAMIL	380
663	0.7013	0.4831	0	23.16804	57.86721	SAMIL	543
664	2.2105	0.0271	2	23.35083	58.01822	SAMIL	362
665	3.5256	0.0004	3	23.36977	58.10900	BID BID	262
666	4.0144	0.0001	3	23.20108	58.08336	BID BID	479
667	3.3161	0.0009	3	23.22768	58.06741	BID BID	445
668	3.8735	0.0001	3	23.23538	58.07876	BID BID	411
669	3.8683	0.0001	3	23.24647	58.23993	BID BID	482
670	1.0021	0.3163	0	23.09884	57.24638	AL HAMRA	654
671	3.7256	0.0002	3	23.33378	58.11761	BID BID	285
672	0.2503	0.8023	0	23.05890	57.12525	BAHLA	595
673	0.3577	0.7206	0	23.05660	57.12477	BAHLA	596
674	3.2735	0.0011	3	23.31680	58.07956	SAMIL	299
675	0.3760	0.7069	0	23.10943	57.29982	AL HAMRA	657
676	0.3037	0.7613	0	23.08475	57.30064	AL HAMRA	649
677	2.0636	0.0391	2	23.34770	58.01365	SAMIL	367
678	0.3881	0.6979	0	23.05238	57.46541	NIZWA	632
679	-0.0429	0.9658	0	23.02059	57.53509	NIZWA	599
680	-0.0089	0.9929	0	22.98806	57.67309	NIZWA	788
681	0.3652	0.7149	0	23.09146	57.14275	BAHLA	623

682	0.3069	0.7589	0	22.98358	57.78392	IZKI	584
683	-0.9775	0.3283	0	22.96801	57.55755	NIZWA	541
684	-2.7711	0.0056	-3	22.36753	57.51806	ADAM	278
685	-0.4645	0.6423	0	22.98374	57.15027	BAHLA	560
686	-0.1881	0.8508	0	22.99851	57.13964	BAHLA	560
687	5.0254	0.0000	3	23.09388	58.34633	AL MUDAYBI	671
688	0.2273	0.8202	0	22.72076	57.99738	AL MUDAYBI	450
689	0.3748	0.7078	0	22.78392	58.00017	AL MUDAYBI	473
690	0.5004	0.6168	0	22.61357	57.99864	AL MUDAYBI	410
691	-0.5912	0.5544	0	22.50131	58.01761	AL MUDAYBI	348
692	0.6162	0.5378	0	22.50011	58.11700	AL MUDAYBI	374
693	-2.9444	0.0032	-3	22.30063	58.06937	AL MUDAYBI	304
694	0.3729	0.7092	0	22.52247	58.12477	AL MUDAYBI	386
695	0.1297	0.8968	0	22.53378	58.11703	AL MUDAYBI	385
696	0.5939	0.5526	0	22.58308	58.12829	AL MUDAYBI	412
697	0.8596	0.3900	0	22.61078	58.13579	AL MUDAYBI	427
698	0.4911	0.6234	0	22.56432	58.12204	AL MUDAYBI	407
699	0.8254	0.4091	0	22.54503	58.17757	AL MUDAYBI	394
700	0.9466	0.3439	0	22.57139	58.19386	AL MUDAYBI	412
701	1.2598	0.2077	0	22.60033	58.18361	AL MUDAYBI	423
702	-1.9846	0.0472	-2	22.38344	58.21683	AL MUDAYBI	323
703	-2.5936	0.0095	-3	22.37300	58.01514	AL MUDAYBI	314
704	-1.9846	0.0472	-2	22.37634	58.21692	AL MUDAYBI	320
705	0.9723	0.3309	0	22.67319	57.99344	AL MUDAYBI	426
706	0.4814	0.6303	0	22.61325	58.00162	AL MUDAYBI	407
707	2.4717	0.0134	2	22.80014	58.13347	AL MUDAYBI	537
708	2.3061	0.0211	2	22.70616	58.17651	AL MUDAYBI	481
709	0.1708	0.8644	0	23.07024	56.96684	IBRI	587
710	0.1489	0.8817	0	23.11580	56.87783	IBRI	527
711	0.1043	0.9170	0	23.10807	57.07340	IBRI	916
712	0.6623	0.5078	0	23.12641	57.08746	IBRI	937
713	-0.0336	0.9732	0	23.05789	56.99660	IBRI	645
714	1.7465	0.0807	1	23.49272	56.82978	IBRI	651
715	-1.9119	0.0559	-1	23.86139	56.40493	YANQL	733
716	-1.7967	0.0724	-1	23.93132	56.39995	YANQL	705
717	-2.4833	0.0130	-2	23.94477	56.34155	YANQL	869
718	0.7492	0.4538	0	23.40133	57.00209	IBRI	950
719	0.5939	0.5526	0	23.16985	56.90982	IBRI	587
720	-1.9987	0.0456	-2	23.83645	56.41027	YANQL	752
721	0.4155	0.6778	0	23.22377	57.03722	IBRI	706
722	0.5912	0.5544	0	23.24793	57.02558	IBRI	761
723	0.0629	0.9498	0	23.07538	56.83961	IBRI	496
724	0.9767	0.3287	0	23.28092	56.89874	IBRI	661
725	0.6743	0.5001	0	23.28632	56.92994	IBRI	703
726	-2.1150	0.0344	-2	23.88424	56.41828	YANQL	697
727	0.6743	0.5001	0	23.37989	56.87896	IBRI	716
728	0.8578	0.3910	0	23.38319	56.91933	IBRI	785
729	0.9349	0.3499	0	23.38516	56.92031	IBRI	790
730	1.0535	0.2921	0	23.37572	56.96644	IBRI	880
731	0.7747	0.4385	0	23.48436	57.00106	IBRI	803
732	1.1203	0.2626	0	23.50209	56.89121	IBRI	822
733	1.7599	0.0784	1	23.46499	56.79309	IBRI	603
734	2.4393	0.0147	2	23.43015	56.75464	IBRI	547
735	-2.7606	0.0058	-3	23.93372	56.18469	DANK	847
736	4.1726	0.0000	3	23.23475	58.51719	AL AMRAT	524
737	3.1560	0.0016	3	26.19016	56.23897	KHASAB	14
738	2.8890	0.0039	3	23.37774	58.15498	BID BID	258
739	3.5050	0.0005	3	23.22946	58.07340	BID BID	441
740	1.5706	0.1163	0	23.42778	58.13359	BID BID	206
741	0.5479	0.5837	0	23.49266	58.04511	BID BID	225
742	1.6342	0.1022	0	23.44192	58.09764	BID BID	230
743	1.2887	0.1975	0	23.45175	58.11998	BID BID	176

744	2.6308	0.0085	3	23.41009	58.12452	BID BID	218
745	-2.1150	0.0344	-2	23.88452	56.41585	YANQAL	702
746	-2.2032	0.0276	-2	23.82169	56.40909	YANQAL	777
747	0.7465	0.4553	0	23.07381	57.26491	AL HAMRA	630
748	0.3760	0.7069	0	23.10270	57.31193	AL HAMRA	668
749	-7.4159	0.0000	-3	25.63353	56.23481	DABA	173
750	-1.8554	0.0635	-1	23.92831	56.39195	IBRI	738
751	-2.5982	0.0094	-3	23.94306	56.33760	YANQAL	890
752	-1.9119	0.0559	-1	23.85161	56.36803	YANQAL	925
753	0.4933	0.6218	0	23.13934	56.92596	IBRI	585
754	0.2710	0.7864	0	23.07176	56.95575	IBRI	565
755	0.1268	0.8991	0	23.05111	56.95282	IBRI	577
756	2.0291	0.0424	2	23.41789	58.13206	BID BID	214
757	2.4813	0.0131	2	23.40561	58.14193	BID BID	223
758	-1.3965	0.1626	0	23.56040	56.52949	YANQAL	536
759	0.3145	0.7532	0	23.04534	57.12254	BAHLA	591
760	0.3936	0.6939	0	23.04559	57.11968	BAHLA	593
761	-0.4645	0.6423	0	22.98397	57.15142	BAHLA	553
762	-1.0694	0.2849	0	22.93478	57.33378	BAHLA	569
763	-0.0429	0.9658	0	23.02145	57.53577	NIZWA	598
764	-1.3131	0.1891	0	22.91756	57.30139	BAHLA	542
765	-0.5571	0.5775	0	22.93392	57.28453	BAHLA	527
766	-0.9314	0.3516	0	22.96860	57.55555	NIZWA	547
767	-8.7396	0.0000	-3	25.26917	56.27835	MADHA	0
768	1.7169	0.0860	1	23.14015	56.75863	IBRI	441
769	-0.0112	0.9911	0	23.08567	56.85527	IBRI	506
770	3.8683	0.0001	3	23.23995	58.23441	BID BID	499
771	0.6198	0.5354	0	23.25131	57.03450	IBRI	778
772	0.5204	0.6028	0	23.23043	57.03889	IBRI	718
773	0.3850	0.7002	0	23.08339	57.27924	AL HAMRA	639
774	-1.2520	0.2106	0	22.81731	57.75075	IZKI	446
775	0.0098	0.9922	0	23.37667	57.74161	IZKI	346
776	-8.7396	0.0000	-3	25.26577	56.30993	MADHA	120
777	-0.0089	0.9929	0	22.99115	57.67374	NIZWA	683
778	0.1484	0.8820	0	22.90108	57.76683	IZKI	511
779	-4.6856	0.0000	-3	25.70190	56.27156	DABA	8
780	-7.4159	0.0000	-3	25.63469	56.25139	DABA	16
781	-7.1725	0.0000	-3	25.64310	56.26397	DABA	18
782	0.2587	0.7958	0	23.04934	57.47784	NIZWA	627
783	0.4186	0.6755	0	23.05125	57.13369	BAHLA	595
784	-8.7396	0.0000	-3	25.26863	56.28205	MADHA	0
785	-8.7396	0.0000	-3	25.26440	56.29534	MADHA	160
786	-2.0574	0.0396	-2	23.81766	56.39691	YANQAL	788
787	-2.0285	0.0425	-2	23.87748	56.41280	YANQAL	712
788	-1.9138	0.0556	-1	23.98597	56.49095	YANQAL	470
789	-2.5982	0.0094	-3	23.94415	56.33870	YANQAL	888
790	0.0094	0.9925	0	23.04191	57.45879	NIZWA	610
791	0.3807	0.7034	0	24.23822	55.79237	AL BURAYMI	293
792	-7.4159	0.0000	-3	25.63403	56.25108	DABA	13
793	3.1560	0.0016	3	26.18938	56.24147	KHASAB	19
794	-6.9630	0.0000	-3	25.66646	56.25043	DABA	13
795	2.6308	0.0085	3	23.40008	58.13779	BID BID	241
796	-1.0694	0.2849	0	22.93461	57.33367	BAHLA	570
797	3.9891	0.0001	3	23.26243	58.23025	BID BID	451
798	0.7120	0.4765	0	23.48820	58.10532	BID BID	154
799	0.2506	0.8021	0	23.08392	57.28381	AL HAMRA	643
800	1.1694	0.2422	0	23.45256	58.11155	BID BID	195
801	1.2111	0.2259	0	23.45061	58.11736	BID BID	181
802	2.1448	0.0320	2	23.29614	57.97556	SAMIL	399
803	2.8890	0.0039	3	23.38162	58.14727	BID BID	247
804	-0.0429	0.9658	0	23.02076	57.53566	NIZWA	598
805	-0.5419	0.5879	0	22.98461	57.13863	BAHLA	555

806	0.4293	0.6677	0	23.07154	57.12929	BAHLA	602
807	3.8004	0.0001	3	23.22504	58.07768	BID BID	430
808	2.4813	0.0131	2	23.40298	58.13891	BID BID	227
809	-1.7854	0.0742	-1	24.36711	56.06808	MAHADAH	681
810	0.3511	0.7255	0	23.08405	57.29630	AL HAMRA	646
811	0.3760	0.7069	0	23.10482	57.30747	AL HAMRA	654
812	0.2968	0.7666	0	22.91783	57.76733	IZKI	530
813	-2.6727	0.0075	-3	24.36828	56.30100	MAHADAH	750
814	-8.7396	0.0000	-3	25.26660	56.31658	MADHA	113
815	-0.8532	0.3936	0	22.97042	57.54886	NIZWA	544
816	-1.0484	0.2945	0	22.93403	57.53406	NIZWA	515
817	-0.0118	0.9906	0	23.02019	57.53823	NIZWA	601
818	-0.4754	0.6345	0	22.73650	57.61560	MANAH	388
819	-0.2494	0.8031	0	22.76803	57.76803	IZKI	457
820	-6.9630	0.0000	-3	25.65725	56.26367	DABA	16
821	-7.9091	0.0000	-3	25.61469	56.25552	DABA	18
822	-0.5259	0.5990	0	22.95139	57.30044	BAHLA	538
823	-0.4503	0.6525	0	23.00017	57.30133	BAHLA	583
824	-1.8866	0.0592	-1	24.55137	56.15500	MAHADAH	760
825	-0.0331	0.9736	0	23.10147	57.83381	SAMIL	640
826	-0.0519	0.9586	0	23.39014	57.82942	IZKI	307
827	5.0954	0.0000	3	23.12222	58.36229	AL MDAYBI	621
828	4.6757	0.0000	3	23.16167	58.31136	BID BID	740
829	2.1935	0.0283	2	23.30447	57.53155	AL RUSTAQ	494
830	0.4433	0.6575	0	23.49341	57.45483	AL RUSTAQ	262
831	-2.0285	0.0425	-2	23.86895	56.40910	YANQAL	728
832	0.3696	0.7117	0	23.39036	57.31413	AL RUSTAQ	450
833	0.4399	0.6600	0	23.36798	57.30734	AL RUSTAQ	507
834	1.5181	0.1290	0	23.21073	57.33719	AL RUSTAQ	1132
835	1.6598	0.0970	1	23.21908	57.31668	AL RUSTAQ	957
836	1.0543	0.2917	0	23.28415	57.32780	AL RUSTAQ	662
837	0.2710	0.7864	0	23.07053	56.96018	IBRI	569
838	0.0446	0.9644	0	23.05325	56.94782	IBRI	569
839	0.7373	0.4610	0	23.14729	57.08104	IBRI	962
840	-0.0018	0.9985	0	23.10119	57.06797	IBRI	961
841	-7.9091	0.0000	-3	25.61686	56.25006	DABA	15
842	-0.3712	0.7105	0	22.88461	57.76728	IZKI	499
843	-1.3688	0.1711	0	22.81817	57.75100	IZKI	451
844	-1.3688	0.1711	0	22.81700	57.75033	IZKI	448
845	0.1778	0.8589	0	23.08250	57.28208	AL HAMRA	642
846	2.6308	0.0085	3	23.39790	58.13994	BID BID	244
847	2.9744	0.0029	3	23.37671	58.15881	BID BID	272
848	1.5467	0.1219	0	23.18610	57.62625	AL AWABI	825
849	1.4943	0.1351	0	23.21845	57.55471	AL AWABI	672
850	2.3613	0.0182	2	23.30894	57.53024	AL RUSTAQ	504
851	-1.9718	0.0486	-2	23.88444	56.41938	YANQAL	694
852	-1.8247	0.0680	-1	23.86313	56.38962	YANQAL	770
853	3.1299	0.0017	3	23.31703	58.06831	SAMIL	299
854	2.8530	0.0043	3	23.28004	58.03634	SAMIL	372
855	1.0404	0.2981	0	23.43369	57.12727	AL RUSTAQ	641
856	-2.2005	0.0278	-2	23.94119	56.35036	YANQAL	845
857	0.6479	0.5170	0	23.11966	57.28446	AL HAMRA	668
858	-0.8212	0.4115	0	22.97354	57.55395	NIZWA	542
859	2.2105	0.0271	2	23.35050	58.01808	SAMIL	363
860	5.1653	0.0000	3	23.10755	58.30604	BID BID	709
861	5.1255	0.0000	3	23.11211	58.30912	AL MDAYBI	710
862	-0.5775	0.5636	0	22.88179	57.52885	NIZWA	477
863	5.8066	0.0000	3	23.18929	58.98217	QURAYYAT	14
864	2.8186	0.0048	3	23.36050	58.05072	SAMIL	310
865	-0.0935	0.9255	0	23.03829	57.45174	NIZWA	616
866	-0.0118	0.9906	0	23.02042	57.53748	NIZWA	600
867	2.1139	0.0345	2	22.98497	58.01725	SAMIL	675

868	0.1858	0.8526	0	23.08344	56.81719	IBRI	476
869	3.5065	0.0005	3	23.08419	58.10056	SAMIL	798
870	0.6740	0.5003	0	23.16728	56.90164	IBRI	572
871	2.2105	0.0271	2	23.30823	57.99949	SAMIL	373
872	-1.7870	0.0739	-1	24.21216	55.97160	AL BURAYMI	414
873	-1.6823	0.0925	-1	23.94016	56.39288	YANQAL	689
874	-2.1436	0.0321	-2	23.93530	56.34955	YANQAL	875
875	-0.4357	0.6630	0	23.42499	57.81114	IZKI	265
876	0.4732	0.6361	0	23.23519	57.04537	IBRI	717
877	-1.9119	0.0559	-1	23.85136	56.36761	YANQAL	943
878	4.2717	0.0000	3	23.21753	58.13397	BID BID	624
879	0.4885	0.6252	0	23.13464	56.90039	IBRI	549
880	-8.7396	0.0000	-3	25.28169	56.32339	MADHA	86
881	-8.7396	0.0000	-3	25.27984	56.32140	MADHA	86
882	-8.7396	0.0000	-3	25.26439	56.30745	MADHA	129
883	1.2887	0.1975	0	23.44781	58.11973	BID BID	186
884	1.1833	0.2367	0	23.44541	58.12529	BID BID	187
885	-7.9091	0.0000	-3	25.61689	56.25011	DABA	15
886	-3.2275	0.0012	-3	24.20655	56.23756	AL BURAYMI	514
887	0.0951	0.9242	0	23.04683	56.94413	IBRI	573
888	-3.0457	0.0023	-3	24.03439	56.21689	AL BURAYMI	793
889	0.2087	0.8347	0	23.06515	56.94324	IBRI	559
890	0.8555	0.3923	0	23.48013	58.08637	BID BID	201
891	3.1560	0.0016	3	26.19610	56.21940	KHASAB	17
892	0.6623	0.5078	0	23.14350	57.08689	IBRI	953
893	0.6623	0.5078	0	23.14558	57.08797	IBRI	954
894	-1.8866	0.0592	-1	24.55064	56.10094	AL BURAYMI	640
895	-2.3841	0.0171	-2	24.70144	56.16747	MAHADAH	479
896	1.0749	0.2824	0	23.30054	57.31341	AL RUSTAQ	651
897	1.6213	0.1050	0	23.22895	57.33003	AL RUSTAQ	806
898	1.5181	0.1290	0	23.21781	57.33344	AL RUSTAQ	880
899	0.5573	0.5773	0	23.55288	57.47745	AL RUSTAQ	200
900	0.4194	0.6749	0	23.45291	57.06256	AL RUSTAQ	747
901	-0.0232	0.9815	0	23.38461	57.83319	NAKHAL	331
902	-0.4955	0.6202	0	23.42685	57.81598	VADI AL MAAWI	262
903	-0.7215	0.4706	0	23.67897	58.13694	AlMaabailah	10
904	0.5259	0.5990	0	23.56999	58.12154	Old AlKhoudh	92
905	0.6480	0.5170	0	23.54225	58.18989	AlJafnain	78
906	1.6762	0.0937	1	23.53911	58.39925	AlHammam	93
907	1.9668	0.0492	2	23.48700	58.34746	Old Bosher	136
908	2.2300	0.0257	2	23.52885	58.50102	adenat AlNahdha	84
909	3.4142	0.0006	3	23.40708	58.51533	AlHajir	143
910	3.4243	0.0006	3	23.26019	58.59108	Bii	354
911	2.3551	0.0185	2	23.50692	58.66122	Yiti	20
912	3.0835	0.0020	3	23.46853	58.67122	AlHilo/Muscat	46
913	5.5029	0.0000	3	23.43614	58.76942	AlSeefah/Muscat	14
914	5.6993	0.0000	3	23.27050	58.91337	Daghmar/Quraya	12
915	6.6386	0.0000	3	23.03425	58.99178	di AlArbiyeen/Qur	101
916	6.4137	0.0000	3	23.07550	59.05028	Dhabab/Qurayat	12
917	3.6517	0.0003	3	22.80764	59.24750	Tiwi/Sur	17
918	2.2974	0.0216	2	22.54858	59.48400	Bilad Sur	14
919	5.1195	0.0000	3	23.12272	58.46286	dah/Dama wa Att	499
920	7.1864	0.0000	3	22.96125	58.81017	ni Said/Dama wa	362
921	0.7765	0.4374	0	23.46967	58.10386	Fanja	163
922	2.1805	0.0292	2	23.41565	58.12932	/Interior of Bidbic	217
923	3.4417	0.0006	3	23.37264	58.10689	Suroor/Samail	257
924	0.7042	0.4813	0	23.14006	57.84561	di Bani Rawaha/A	572
925	4.0774	0.0000	3	23.23356	58.08106	AlFatih	422
926	0.0813	0.9352	0	22.02429	59.31360	Bani Bo Ali/Hadra	97
927	2.2974	0.0216	2	22.54058	59.46614	Bani Bo Hassan/F	20
928	2.2931	0.0218	2	22.21553	59.20647	AlDreez/AlKamil	175
929	3.1937	0.0014	3	22.50761	59.12470	Sabit	395

930	3.6539	0.0003	3	22.59669	59.08558	di Bani Khalid/Mu	610
931	5.7128	0.0000	3	22.59404	58.82853	di Bani Khalid/AlH	411
932	3.4009	0.0007	3	23.02169	58.21719	lJarda/AlMudhail	669
933	4.5967	0.0000	3	22.88525	58.22347	Samad/AlRawdah	613
934	-2.1283	0.0333	-2	22.37053	58.05928	Barzaman	306
935	4.9640	0.0000	3	22.84189	58.39250	Ibra/AlQaa	568
936	4.4557	0.0000	3	22.57866	58.68451	AlQabil	355
937	3.8606	0.0001	3	22.43644	58.78045	Bidiyeh	302
938	-0.9775	0.3283	0	23.13976	57.73595	Wakan/Nakhal	1362
939	0.2244	0.8225	0	23.51061	57.91903	adi AlMaawal/AlA	154
940	0.2273	0.8202	0	23.68274	57.80946	lHafri/Barka Coas	22
941	0.5864	0.5576	0	23.49336	57.00875	AlHayal/Ibri	763
942	2.8275	0.0047	3	23.31200	56.61753	AlDiriez	425
943	-2.0059	0.0449	-2	23.40242	56.28156	Ibri/AlMazim	294
944	0.6869	0.4921	0	23.48626	57.30645	lMadinah/Rustaq	344
945	1.7196	0.0855	1	23.56572	57.56389	Gama	140
946	1.3626	0.1730	0	23.84183	57.09944	naborah/Bani Rab	89
947	-1.9987	0.0456	-2	23.83638	56.36104	Wadi AlHareem	806
948	-2.0874	0.0369	-2	23.63230	56.48832	anqul/AlBuweedra	597
949	-3.3281	0.0009	-3	24.15581	56.88522	AlHaweel	10
950	-8.4531	0.0000	-3	25.60620	56.25627	ba/Oman AlArab	15
951	-1.0298	0.3031	0	26.07364	56.10739	Tibaat	16
952	0.2831	0.7771	0	26.12186	56.13964	Bakha/Fadhgha	17
953	2.2934	0.0218	2	26.16395	56.17503	AlJadii	16
954	3.1560	0.0016	3	26.19319	56.24144	Khasab/AlHajer	14
955	3.1560	0.0016	3	26.19287	56.22054	Khasab/Kada	15
956	0.7187	0.4723	0	23.73714	57.66125	Abu Abali	16
957	-0.4100	0.6818	0	22.71776	57.55005	Manah/Aizz	392
958	-2.4719	0.0134	-2	22.38153	57.52144	am/Gamaa Albus	291
959	-0.9319	0.3514	0	21.80692	57.91725	Adam/Brimah	154
960	0.3321	0.7398	0	23.05228	57.46031	Tanuf	626
961	0.0137	0.9890	0	23.04598	57.67472	Masserit AlRawajl	1049
962	-1.2640	0.2062	0	22.82019	57.75317	Qalaat AlAwamir	461
963	-6.4532	0.0000	-3	24.80469	56.44728	AlAqur	15
964	-3.0912	0.0020	-3	24.39872	56.70775	ohar/AlKhashaba	7
965	-4.2296	0.0000	-3	24.24831	56.78261	Sohar/AlAweenat	25
966	-2.2068	0.0273	-2	24.55086	56.56514	Liwa/AlZahiyah	13
967	-2.1918	0.0284	-2	24.73451	55.94840	hadhah AlRawdh	426
968	-1.7370	0.0824	-1	24.54719	56.10608	lahadhah AlJuwe	642
969	-3.1814	0.0015	-3	23.61858	55.96711	lNasinah AlRihan	268
970	0.6852	0.4932	0	23.13810	57.31007	Misfaat' AlAbrieen	911
971	-1.4539	0.1460	0	23.51683	56.54022	Dhank/Fida	498
972	-1.5551	0.1199	0	23.56227	56.26650	hank/Wadi Dhan	358
